# The measurement of China’s provincial physical capital stock based on the improved method and indicators

**DOI:** 10.1371/journal.pone.0307946

**Published:** 2024-08-13

**Authors:** Qi Wang, Ziyu Ma

**Affiliations:** School of Finance, Tongling University, Tongling, Anhui, China; National University of Sciences and Technology, PAKISTAN

## Abstract

Physical capital stock is the basic indicator of macroeconomic empirical studies and also an important manifestation of macroeconomic activity capacity. When measuring China’s provincial physical capital stock by the perpetual inventory method, the treatment for the depreciation rate and the initial physical capital stock in the existing literature has some defects in reflecting the economic performance. The purpose of this paper is to build the most reliable and reasonable statistical series of China’s provincial physical capital stock. We establish a measurement formula in form of the variable depreciation rate and improve the treatment for the depreciation rate and the initial physical capital stock. For the depreciation rate, we measure the benchmark depreciation rate of physical capital and determine the variable depreciation rate according to the economic growth rate, which makes the variable depreciation rate reflect the economic growth. For the initial physical capital stock, we measure the initial physical capital stock according to the capital output ratio, which makes the initial physical capital stock reflect the economic aggregate. Based on these improvements, we measure China’s provincial physical capital stock from 1952 to 2022 by the perpetual inventory method. Through analyzing the measurement results, we have a more distinct insight into the temporal and spatial characteristics of China’s provincial physical capital stock: The growth rate of physical capital stock at the provincial level has slowed in spite of remaining high; The eastern region is much higher than the central and western regions. According to these two characteristics, it is suggested that policymakers need to improve market mechanisms and adjust investment policies to promote a benign profile of economic growth and a regional optimization of capital formation.

## 1. Introduction

Physical capital stock is the basic indicator of macroeconomic empirical studies. Empirical studies on many areas such as economic growth, business cycle and total factor productivity all need physical capital stock. Only by accurately measuring physical capital stock data can macroeconomic empirical studies be carried out reasonably. Undergoing the development for more than 70 years, China has become the world’s second largest economy. How to explain the reasons for China’s economic growth, how to find the sources for China’s economic growth and how to evaluate the impetuses for China’s economic growth are inseparable from physical capital stock. However, there are still no official statistics issued by China’s National Bureau of Statistics for physical capital stock at the national and provincial levels.

As socialism with Chinese characteristics has entered a new era, China’s economy has transitioned from a stage of rapid growth to a stage of high-quality development. At this stage, the biggest challenge or feature for China’s economy is the medium-low growth rate. Data from China’s Bureau of Statistics and the World Bank shows that China’s GDP growth has dropped from 7.4% in 2014 to 5.2% in 2023, with an average of 6.0% which lowers than an average of 10.3% in the previous decade. Other challenges for China’s economy include but are not limited to the ongoing impact of the COVID-19 pandemic, the tight financial credit, the unbalanced financial situation and the serious local government debt problem. To maintain continuous economic growth and alleviate other challenges, China’s central government has revamped economic outlines. In the field of traditional industries, it is leading to eliminate outdated capacity and adjust industrial structure. In the field of emerging industries, it is promoting to apply new technologies and increase research investment. The Communist Party of China and China’s central government have set forth a grand blueprint for realizing China’s rejuvenation. Given that physical capital is an essential factor of economic growth, realistic challenges and future goals put forward higher requirements for exerting physical capital to support economic growth. How to understand physical capital from the perspective of sustainable development and make good use of physical capital in the stage of high-quality development, we need to measure physical capital stock reasonably. With a clear insight of China’s physical capital stock at the national and provincial levels, we can rationally judge whether and how relevant policies need to be adjusted to promote high-quality development of the national economy and coordinated development of the regional economy.

Previous studies have measured China’s provincial physical capital stock by the perpetual inventory method (PIM) [1–5] and of which Zhang et al. (2004) [1] and Shan (2008) [2] are typical ones. The former covers the period from 1952 to 2000, while the latter covers the period from 1952 to 2006. The two studies have been providing important data for China’s macroeconomic empirical research. In these studies, although the measurement indicators are gradually optimized, there are still some defects. Due to the measurement methods and data sources, these defects mainly focus on the selection and identification for the depreciation rate and the initial physical capital stock. First, the determination for the depreciation rate in the existing literature cannot fully reflect the economic growth. A majority of the studies adopt the constant depreciation rate which ignores the economic changes. A minority of the studies adopt the variable depreciation rates which remote from economic reality, lack statistical support or rely on model construction. The reason for the defects on the depreciation rate is that neither a feasible measurement methodology with the variable depreciation rate is constructed, nor an appropriate variable depreciation rate series is selected. Second, the measurement for the initial physical capital stock in the existing literature cannot fully reflect the economic performance, which is generally manifested in the following aspects: (1) The growth rate method relies on the growth rate of the investment flow; (2) The steady state method relies on the steady state condition; (3) The parameter method relies on the production function; (4) The integral method of investment flow relies on the growth rate of the investment flow and ignores the depreciation of physical capital. The reason for the defects on the initial physical capital stock is that the relationship between is ignored.

Physical capital is one of the important driving forces for high-quality economic development. How to reflect high-quality economic development from the perspective of physical capital, we need to improve and renovate the measurement method for physical capital stock. Modifying these defects on the depreciation rate and the initial physical capital stock in the existing literature will constitute one of the main contents of this paper. The purpose of this paper is to construct a feasible measurement methodology with the variable depreciation rate and further build the most reliable and reasonable statistical series of China’s provincial physical capital stock. In view of the defects in the treatment of the depreciation rate and the initial physical capital stock, we establish a measurement formula in form of the variable depreciation rate and improve the treatment of the depreciation rate and the initial physical capital stock. For the variable depreciation rate, we determine it according to the economic growth rate, which makes it reflect the economic growth. For the initial physical capital stock, we measure it according to the capital output ratio, which makes it reflect the economic aggregate. Based on these improvements, we measure China’s provincial physical capital stock from 1952 to 2022 by the PIM (excluding Hongkong, Macao and Taiwan). According to the measurement results, we have a more distinct understanding of the temporal and spatial characteristics of China’s provincial physical capital stock.

The marginal contribution of our work to the measurement of China’s provincial physical capital stock is as follows: First, it establishes a measurement formula in form of the variable depreciation rate. When measuring China’s provincial physical capital stock with the variable depreciation rate methodology, previous studies do not establish a measurement formula in form of the variable depreciation rate, but mistake a measurement formula in form of the constant depreciation rate as one in form of the variable depreciation rate, which results in the disconnection between theory and practice. For this defect, we establish a measurement formula in form of the variable depreciation rate, which provides a theoretical framework for measuring China’s provincial physical capital stock. Second, it measures the variable depreciation rate reflecting economic growth and the initial physical capital stock reflecting economic aggregate. After establishing a measurement formula in form of the variable depreciation rate, we can adopt the variable depreciation rate to overcome the defect that the constant depreciation rate ignores the economic changes. When measuring China’s provincial physical capital stock with the variable depreciation rate indicator, previous studies determine the variable depreciation rate by simple selection or regression fitting and fail to find that from economic reality. For this defect, we measure the variable depreciation rate reflecting economic growth and the initial physical capital stock reflecting economic aggregate, which provides a methodological extension for measuring China’s provincial physical capital stock. Third, it further expands the time span of China’s provincial physical capital stock. We measure China’s provincial physical capital stock from 1952 to 2022 and provide important data support for discussing China’s economic growth in different stages. To guarantee the consistency of statistical indicators and data sources as much as possible, we use the same statistical indicators and data sources in most years and only supplement missing indicators and data in a few years.

The rest of the paper proceeds as follows. Section 2 provides a literature review. Section 3 elaborates the measurement method. Section 4 describes the indicators and data. Section 5 presents the measurement results and analyzes the temporal and spatial characteristics. Section 6 concludes the study.

## 2. Literature review

The measurement of physical capital stock is the basic work for carrying out macroeconomic empirical research. The measurement methods of physical capital stock include the direct survey method, the fixed assets balance sheet method and the PIM. When measuring physical capital stock, the direct survey method and the fixed assets balance sheet method are laborious, while the PIM is convenient. Very few countries (Japan, South Korea, Netherlands) have adopted the direct survey method and some countries (Central and Eastern European countries, CIS countries) once adopted the fixed assets balance sheet method. By contrast, most countries (OECD countries, China, Singapore, etc.) have been adopting the PIM. As the PIM was successfully proposed [6] and continuously improved [7–10], it has become a universal measurement method for physical capital stock in the world [11–13].

The studies on measuring China’s physical capital stock by the PIM began in the 1990s [14]. From the perspective of methodological levels, these studies can be divided into two categories: the traditional method and the untraditional method [12, 15, 16]. The measurement formula of the traditional method is expressed as follows:

$$K_{t}=(1-D)K_{t-1}+I_{t}$$ (1)

In Eq (1), *K* denotes the physical capital stock, *D* denotes the depreciation rate, *I* denotes the investment flow and *t* marks the period. In terms of that whether the depreciation rate is variable, the studies by the traditional method can be divided into two subcategories: the constant depreciation rate method and the variable depreciation rate method. *D* of the former is the constant and *D* of the latter is the variable. The theoretical premise of the constant depreciation rate method is the assumption that the age-efficiency (the relative efficiency) of a capital good matches the Geometrically Decreasing pattern [17]. The constant depreciation rate method is easier to operate because of the low requirement of the depreciation rate, which makes this method the primary choice in the existing literature [12, 18–20]. However, in theory, the accuracy of measurement results obtained by this method is low [21]. In order to reflect the time fluctuation or the regional difference of the depreciation rate more reasonably and capture the accumulation of physical capital more accurately, some studies attempt to use the variable depreciation rate method [4, 5, 22–25]. The variable depreciation rate method is more complicated to operate because of the higher requirement of the depreciation rate. In theory, the accuracy of measurement results obtained by this method is higher. However, the existing literature has not derived a measurement formula in form of the variable depreciation rate, but mistake a measurement formula in form of the constant depreciation rate as one in form of the variable depreciation rate. In addition to these two methods, some studies turn to the untraditional method. The measurement formula of that is expressed as follows:

$$K_{t}=\sum_{\tau =0}^{T}h_{\tau }∙F_{\tau }∙I_{t-\tau }$$ (2)

In Eq (2), *T* and *τ* denote the service life and the service age of a capital good respectively, *h* and *F* denote the age-efficiency function and the retirement function of a capital good respectively. Compared with the traditional method, the untraditional method has three characteristics: (1) It introduces more age-efficiency functions (relative efficiency functions) and retirement functions; (2) It considers more age-efficiency profiles and retirement profiles; (3) It excludes the impact of depreciation rate (or the depreciation) on measurement results. It is worth noting that for a cohort of capital goods, the combined age-efficiency and retirement profile often resembles the Geometrically Decreasing pattern. In this case, we are recommended to use the constant depreciation rate method instead of the untraditional method because the former is empirically supported and easily implemented [12, 26]. Of the three methods, the untraditional method is the most complex to operate. In theory, the accuracy of measurement results obtained by this method is the highest. Generally speaking, the accuracy of results obtained by a method is directly proportional to the complexity of this method. However, this does not mean that the most accurate measurement results can be obtained by the untraditional method in practice. The measurement of physical capital stock involves both methods and indicators. If indicators are not appropriate enough, even if we use the most complex method, it is difficult to get accurate results. If we aim at the subjects that require high accuracy of physical capital stock, such as capital return share or capital return rate, we have to use a complex method and adopt accurate indicators to measure physical capital stock meticulously. If we focus on the subjects that require low accuracy of physical capital stock, such as economic growth or policy assessment, we just need to use a simple method and adopt appropriate indicators to measure physical capital stock quickly. Considering that most studies in economics have no specific requirements for the accuracy of physical capital stock, the constant depreciation rate method remains the mainstream and is still widely used [27, 28]. The variable depreciation rate method yields more accurate measurement results because of more reasonable indicators. In contrast, the untraditional method is prone to more errors because it involves more indicators. From the perspective of regional levels, these studies can be divided into two categories: the national level measurements and the provincial level measurements. The national level measurements have emerged in the 1990s [14, 29, 30]. To get a fuller picture of China’s economic growth, the measurements of physical capital stock in the subregions have been put on agenda. The provincial level measurements have emerged in the 2000s [1, 2, 31]. Both of them have been providing abundant data for analyzing China’s economic growth [27, 28, 32]. These studies provide important support for promoting measurement methods and modifying measurement indicators and develop helpful references for explaining China’s economic growth and evaluating China’s economic potential.

However, there are still some controversies on measuring China’s provincial physical capital stock by the PIM despite of many studies in the existing literature. As can be seen in Table 1, although the existing literature has reached a consensus on the investment flow and the price index, it still remains some controversies on the depreciation rate and the initial physical capital stock.

**Table 1:** Comparison of representative studies on China’s provincial physical capital stock.

Authors	Investment Flow	Price Index	Depreciation Rate	Initial Physical Capital Stock
Zhang et al. (2004) [1]	GFCF	IPIIFA PIIFA	9.6%	GRM
Liu and Hu (2006) [31]	GFCF	IPIIFA	9.6%	GRM
Shan (2008) [2]	GFCF	IPIIFA PIIFA	10.96%	GRM
Huang and Gong (2010) [33]	GFCF	IPIIFA	5% (1952–1977) 9.6% (1978–2007)	SSM
Sun et al. (2010) [34]	GFCF	IPIIFA	6%	GRM
Ye (2010b) [3]	GFCF	GDP deflator PIIFA	7.73% for construction 18.22% for equipment	CORM
Xie and Pan (2011) [22]	GFCF	IPIIFA PIIFA	variable (1952–1984) depreciation (1985–2009)	GRM
Fan et al. (2011) [23]	TIFA	RPI PIIFA	5% (1952–1977) variable (1978–2007)	GRM
Jing (2013) [4]	GFCF	IPIIFA PIIFA	3% (1952–1977) 5% (1978–1992) depreciation (1993–2010)	GRM
Jia and Zhang (2014) [24]	GFCF	IPIIFA PIIFA	variable	PM
Chen (2020) [5]	GFCF	PIIFA	variable	GRM IMIF

The gross fixed capital formation (GFCF) is better than the total investment in fixed assets in the whole country (TIFA) and other indicators for its relatively complete data and quite comprehensive content comprehensive content which make it become a popular selection as the investment flow. The price indices for investment in the fixed assets (PIIFA) is the first consideration as the price index and the implied price indices for investment in the fixed assets (IPIIFA) can be substituted for the PIIFA when the latter is unavailable [1, 2, 4].

For the depreciation rate, there are two kinds of selections in the existing literature: the constant depreciation rate and the variable depreciation rate. A majority of the studies adopt the constant depreciation rate for convenience. Although the adoption of the constant depreciation rate is accessible, it ignores the economic changes. A minority of the studies adopt the variable depreciation rate to remedy the defect that the constant depreciation rate cannot reflect the economic changes. The adoption of the variable depreciation rate mainly involves three approaches: reference to official regulations [4, 22], subjective setting [23] and econometric estimation [5, 24, 25]. However, there are limitations in these three approaches. To be specific: Reference to official regulations remotes from economic reality because it just adopts the lower basic depreciation rate of fixed assets of state-owned enterprises, while subjective setting lacks statistical support. Although the annual difference of the depreciation rate is realized to some extent by the above approaches, the regional difference of that is still unsolved. Econometric estimation makes the depreciation rate variable across years and regions. Nevertheless, this approach relies on the model construction, which tends to suffer from the problems of goodness of fit and set of variables. Since the depreciation rate has a significant impact on measurement results of physical capital stock [18, 35], the existing literature has a great dispute on it. Only by constructing a variable depreciation rate series which reflects the economic changes can we measure China’s provincial physical capital stock more objectively.

For the initial physical capital stock, a big part of the studies measure it by the GRM [1, 2, 4], while a small part of the studies measure it by the capital output ratio method (CORM) [3], the steady state method (SSM) [33], the parameter method (PM) [24] or the integral method of investment flow (IMIF) [5]. However, there are still flaws in the above methods. The GRM is the transformation of the SSM or the IMIF and contains the flaws of these two methods, that is, the SSM relies on a strict steady state condition, while the IMIF requires a steady growth rate of the investment flow and ignores the depreciation of physical capital. The PM relies on a well-behaved production function. Although Ye (2010b) [3] adopts the CORM, he fails to show how to determine the capital output ratio. Since the initial physical capital stock has a small impact on measurement results of physical capital stock [36], the existing literature has a little dispute on it. Although we can ignore the impact of physical capital stock in the initial period on those in the later periods, only by constructing the initial physical capital stock which reflects the economic aggregate can we analyze the early changes of China’s provincial physical capital stock more accurately.

To summarize, in terms of measuring China’s physical capital stock by the PIM, there is still some space for improvement on the depreciation rate and the initial physical capital stock even though previous studies have tried helpful attempts. In order to better measure China’s provincial physical capital stock and objectively reflect the economic performance, we establish a measurement formula in form of the variable depreciation rate and improve the treatment for the depreciation rate and the initial physical capital stock. For the depreciation rate, we measure the benchmark depreciation rate of physical capital and determine the variable depreciation rate according to the economic growth rate, which makes the variable depreciation rate reflect the economic growth. For the initial physical capital stock, we measure the initial physical capital stock according to the capital output ratio, which makes the initial physical capital stock reflect the economic aggregate. On the basis of these work, we build the most reliable and reasonable statistical series of China’s provincial physical capital stock.

## 3. Method

The fundamental principle of the PIM is that the current physical capital stock is equal to the weighted sum of the past investment flow at constant prices. According to the fundamental principle, the measurement formula for the PIM is expressed as follows:

$$K_{t}=\sum_{\tau =0}^{\infty }e_{\tau }I_{t-\tau }$$ (3)

In Eq (3), *K* denotes the physical capital stock, *e* denotes the weight describing the relative efficiency of a capital good which means the marginal output efficiency of an old capital good relative to a new one, *I* denotes the investment flow at constant prices, *τ* denotes the service age and *t* marks the period. Let *L* be the service life of a capital good, the relative efficiency *e*_*τ*_ satisfies:

$$e_{0}=1,\;e_{L}=0,\;e_{\tau }-e_{\tau -1}\leq 0,\;\tau =1,2,3\ldots ,L$$ (4)

That is, the relative efficiency of a capital good is 1 at the initial time while is 0 at the retirement time and the relative efficiency of a capital good is nonincreasing. Below we establish a measurement formula in form of the variable depreciation rate on the basis of analyzing the replacement and the depreciation.

### 3.1. Replacement requirement, replacement rate and replacement distribution

Let *m*_*τ*_ be the mortality rate of a capital good at age *τ* which denotes the efficiency loss of a capital good at age *τ* or the proportion of that to be replaced at age *τ*. The mortality rate *m*_*τ*_ satisfies:

$$m_{\tau }=e_{\tau -1}-e_{\tau }=-(e_{\tau }-e_{\tau -1})\geq 0,\;\tau =1,2,3\ldots ,L$$ (5)

$$\sum_{\tau =1}^{L}m_{\tau }=-\sum_{\tau =1}^{L}\left(e_{\tau }-e_{\tau -1}\right)=e_{0}=1$$ (6)

The sequence of the mortality rate {*m*_*τ*_} is referred to as the mortality distribution [17].

Let *R*_*t*_ be the replacement requirement of a capital good in period *t* which denotes the investment to keep the stock of the capital good unchanged in period *t*. Using the mortality distribution {*m*_*τ*_}, the replacement requirement *R*_*t*_ can be expressed in terms of the past investment flow. According to Eqs (3), (4) and (5), the replacement requirement *R*_*t*_ satisfies:

$$K_{t}-K_{t-1}=I_{t}-\sum_{\tau =1}^{\infty }m_{\tau }I_{t-\tau }=I_{t}-R_{t}$$ (7)

$$R_{t}=\sum_{\tau =1}^{\infty }m_{\tau }I_{t-\tau }$$ (8)

Let *r*_*τ*_ be the replacement rate of a capital good at age *τ* which denotes the proportion of an initial investment replaced in period *τ* after the time of acquisition to ensure that the relative efficiency of the capital good at age *τ* is equal to that at initial time. By the convolution equation or the renewal equation [37], the replacement rate *r*_*τ*_ satisfies:

$$r_{\tau }=m_{1}r_{\tau -1}+m_{2}r_{\tau -2}+\cdots +m_{\tau }r_{0},\;\tau =0,1,2,3\cdots$$ (9)

The sequence of the replacement rate {*r*_*τ*_} is referred to as the replacement distribution. Using the replacement distribution {*r*_*τ*_}, the replacement requirement *R*_*t*_ can be expressed in terms of the past changes in the stock of a capital good. According to Eqs (3) and (9), the replacement requirement *R*_*t*_ is expressed as follows:

$$R_{t}=\sum_{\tau =1}^{\infty }r_{\tau }(K_{t-\tau }-K_{t-\tau -1})$$ (10)

Eqs (8) and (10) are equivalent.

### 3.2. Rental price and depreciation

The price-quantity duality system of the durable goods shows that there is a one-to-one correspondence between the stock and the price of a capital good [38]. Let $P_{t}^{I}$ be the price of a capital good which denotes the market price of that in period *t* under the condition of perfect competition. Provided that capital goods at different periods are fully fungible and that the rental price of an old capital good is proportional to the rental price of a new one, the price $P_{t}^{I}$ is equal to the weighted sum of the future rental prices:

$$P_{t}^{I}=\sum_{\tau =0}^{\infty }e_{\tau }\prod_{s=1}^{\tau +1}\frac{1}{1+q_{t+s}}P_{t+\tau +1}^{K}$$ (11)

In Eq (11), $P_{t+\tau +1}^{K}$ denotes the rental price of a capital good at period *t*+*τ*+1, *q*_*t*+*s*_ denotes the return rate of that at period *t*+*s*, *e*_*τ*_ denotes the weight (the relative efficiency) and $\prod_{s=1}^{\tau +1}\frac{1}{1+q_{t+s}}$ denotes the discount factor of $P_{t+\tau +1}^{K}$ at period *t*.

Let $P_{t}^{D}$ be the depreciation of a capital good which denotes the compensation value needed to keep the stock of that intact at period *t*. Taking first difference of Eq (11), the depreciation $P_{t}^{D}$ can be expressed in terms of the mortality distribution {*m*_*τ*_} and the future renewal prices. According to Eqs (5) and (11), the depreciation $P_{t}^{D}$ satisfies:

$$P_{t}^{D}=P_{t}^{I}-(1+q_{t})P_{t-1}^{I}+P_{t}^{K}=\sum_{\tau =1}^{\infty }m_{\tau }\prod_{s=1}^{\tau }\frac{1}{1+q_{t+s}}P_{t+\tau }^{K}$$ (12)

We can also express the depreciation $P_{t}^{D}$ as the same result in terms of the replacement distribution {*r*_*τ*_} and present and future changes in the price of a capital good.

### 3.3. Constant replacement rate and constant depreciation rate

There are three main patterns of the relative efficiency of a capital good: (1) “One-horse-shay” pattern, that is, the relative efficiency is a constant over the service life and zero thereafter; (2) “Linearly Decreasing” pattern, that is, the relative efficiency decreases linearly by a fixed value over the service life; (3) Geometrically Decreasing pattern, that is, the relative efficiency decreases geometrically by a fixed proportion over the service life. The relative efficiency, the mortality distribution and the replacement distribution of a capital good in three patterns are presented in Table 2.

**Table 2:** Relative efficiency, mortality distribution and replacement distribution in three patterns.

Patterns	Relative Efficiency	Mortality Distribution	Replacement Distribution
One-horse-shay	$e_{\tau }=\left\{ \begin{array}{l} c,\tau =0,1,\cdots ,L-1\\ 0,\tau =L,L+1,\cdots \end{array}\right.$	$m_{\tau }=\left\{ \begin{array}{c} 0,\tau \neq L\\ 1,\tau =L \end{array}\right.$	$r_{\tau }=\left\{ \begin{array}{c} 0,\tau \neq L,2L,\cdots \\ 1,\tau =L,2L,\cdots \end{array}\right.$
Linearly Decreasing	$e_{\tau }=\left\{ \begin{array}{c} 1-\frac{\tau }{L},\tau =0,1,\cdots ,L-1\\ 0,\tau =L,L+1,\cdots \end{array}\right.$	$m_{\tau }=\left\{ \begin{array}{l} \frac{1}{L},\tau =0,1,\cdots ,L\\ 0,\tau =L+1,L+2,\cdots \end{array}\right.$	$r_{\tau }=\frac{{\left(1+\frac{1}{L}\right)}^{\tau -1}}{L},\tau =0,1,\cdots ,L$
Geometrically Decreasing	$e_{\tau }={\left(1-r\right)}^{\tau },\tau =0,1,\cdots ,L$	$m_{\tau }=r{\left(1-r\right)}^{\tau -1},\tau =0,1,\cdots ,L$	$r_{\tau }=r,\tau =0,1,\cdots ,L$

Suppose that the relative efficiency of a capital good matches the Geometrically Decreasing pattern. According to Eqs (3) and (8), the replacement requirement *R*_*t*_ is expressed as follows:

$$R_{t}=\sum_{\tau =1}^{\infty }m_{\tau }I_{t-\tau }=r\sum_{\tau =1}^{\infty }{{\left(1-r\right)}^{\tau -1}I}_{t-\tau }=r\sum_{\tau =1}^{\infty }e_{\tau -1}I_{t-\tau }=rK_{t-1}$$ (13)

The proportional constant *r* is the replacement rate. We can also express the replacement requirement *R*_*t*_ as the same term with Eqs (3) and (9). According to Eq (12), the depreciation $P_{t}^{D}$ is proportional to the price $P_{t}^{I}$:

$$P_{t}^{D}=\sum_{\tau =1}^{\infty }m_{\tau }\prod_{s=1}^{\tau }\frac{1}{1+q_{t+s}}P_{t+\tau }^{K}=rP_{t}^{I}=\delta P_{t}^{I}$$ (14)

The proportional constant *r* or *δ* is the depreciation rate (see Jorgenson et al. (1987) [17] or Li et al. (1993) [39] for details). The replacement rate *r* and the depreciation rate *δ* are equal to the same constant only when the relative efficiency of a capital good matches the Geometrically Decreasing pattern. In this case, the relationship between the relative efficiency and the depreciation rate of a capital good is expressed as follows:

$$e_{\tau }={\left(1-\delta \right)}^{\tau },\tau =0,1,\cdots ,L$$ (15)

According to Eqs (7), (13) and (14), the measurement formula for the PIM can be expressed as follows:

$$K_{t}=(1-r)K_{t-1}+I_{t}=(1-\delta )K_{t-1}+I_{t}$$ (16)

Note that the depreciation rate *δ* is constant in Eq (16). We refer to Eq (16) as the measurement formula in form of the constant depreciation rate. The measurement formula in this form widely exists in previous studies.

### 3.4. Variable replacement rate and variable depreciation rate

Eq (16) can be used as the measurement formula for physical capital stock only when the depreciation rate remains unchanged. However, the relative efficiencies of capital goods are not all completely coincident with the Geometrically Decreasing pattern [40–42] and the depreciation rates of those are hard to keep constant in economic reality. If the depreciation rate of a capital good is variable, then Eq (16) cannot be used as the measurement formula for physical capital stock. Measuring physical capital stock with Eq (16) will cause confusion between theoretical analysis and practical operation. It does not provide a logical explanation for why the depreciation rate is not variable in theory and does not offer an argument for why the depreciation rate is variable in practice. The key problem here is how to find a measurement formula in form of the variable depreciation rate to make theoretical analysis and practical operation consistent on measuring physical capital stock.

The first crucial work of this paper is how to solve this problem. According to the above elaboration, we find that whether the replacement rate of a capital good is variable or not depends on the relative efficiency of that. The relative efficiency is related to the use intensity. The greater is the use intensity, the greater are the mortality and the replacement rate. The lower is the use intensity, the lower are the mortality rate and the replacement rate. In economic operation, different economic environment will lead to different production states. When the economic growth momentum is good, enterprises maintain higher production load to increase output, which makes the use intensity of a capital good bigger. When the economic growth momentum is bad, enterprises maintain lower production load to reduce cost, which makes the use intensity of a capital good smaller. In order to reflect different use intensities in economic operation of a capital good, we suppose that the relative efficiency of a capital good matches the quasi-Geometrically Decreasing pattern instead of the Geometrically Decreasing pattern, that is, the relative efficiency of a capital good decreases geometrically by a variable proportion over the service life. If the relative efficiency of a capital good matches this pattern, then the relative efficiency and the mortality rate of that satisfies:

$$e_{\tau }=(1-u_{\tau -1})e_{\tau -1}$$ (17)

$$m_{\tau }=u_{\tau -1}e_{\tau -1}$$ (18)

In Eqs (17) and (18), *u* denotes the variable geometrically decreasing proportion.

Let *u*_*t*−1_ be the geometrically decreasing proportion of the relative efficiency at period *t*−1. According to Eqs (3), (8) and (17), the replacement requirement *R*_*t*_ satisfies:

$$\frac{R_{t}}{K_{t-1}}=\frac{\sum_{\tau =1}^{\infty }m_{\tau }I_{t-\tau }}{\sum_{\tau =1}^{\infty }e_{\tau -1}I_{t-\tau }}=\frac{\sum_{\tau =1}^{\infty }{ue_{\tau -1}I}_{t-\tau }}{\sum_{\tau =1}^{\infty }e_{\tau -1}I_{t-\tau }}=u_{t-1}$$ (19)

Let $r_{t}^{\prime }$ be the replacement rate of *K*_*t*_ to *K*_*t*−1_ which denotes the proportion of a capital good which needs to be replaced to keep *K*_*t*_ and *K*_*t*−1_ equal. The replacement rate $r_{t}^{\prime }$ satisfies:

$$r_{t}^{\prime }=\frac{R_{t}}{K_{t-1}}$$ (20)

According to Eqs (19) and (20), the geometrically decreasing proportion *u*_*t*−1_ and the replacement rate $r_{t}^{\prime }$ satisfy:

$$u_{t-1}=r_{t}^{\prime }$$ (21)

According to Eqs (14) and (21), the geometrically decreasing proportion *u*_*t*−1_, the replacement rate $r_{t}^{\prime }$ and the depreciation rate satisfy:

$$u_{t-1}=r_{t}^{\prime }=\delta_{t-1}$$ (22)

The replacement rate $r_{t}^{\prime }$ and the depreciation rate *δ*_*t*−1_ are equal to the same variable when the relative efficiency of a capital good matches the quasi-Geometrically Decreasing pattern. According to Eqs (7), (20) and (22), the measurement formula for the PIM can be expressed as follows:

$$K_{t}=(1-\delta_{t-1})K_{t-1}+I_{t}$$ (23)

Note that the depreciation rate *δ* is variable in Eq (23). We refer to Eq (23) as the measurement formula in form of the variable depreciation rate. Eq (23) can be used as the measurement formula for physical capital stock on condition that the depreciation rate is variable.

From the above analysis, we have made theoretical analysis and practical operation consistent on measuring physical capital stock by explaining the relationship among the relative efficiency, the replacement rate and the depreciation rate of a capital good and establishing a measurement formula in form of the variable depreciation rate on condition that the relative efficiency of a capital good matches the quasi-Geometrically Decreasing pattern and have solved the confusion caused by measuring physical capital stock with Eq (16). At the same time, Eq (23) lays the foundation for the next two crucial work which we will carry out in the following sections.

However, when applying this methodology in practice, we are faced with the challenge of how to determine the variable depreciation rate. How to find a variable depreciation rate series is an important part of successfully implementing the measurement formula in form of the variable depreciation rate. On account of that statistical agencies do not provide credible or available data on the variable depreciation rate, we must measure that. As we mentioned in Section 2, although a minority of studies adopt the variable depreciation rate to realize the annual and the regional differences of the depreciation rate to some extent, there are still defects in them. Drawing on previous practices on the deprecation rate, we develop a measurement method for the variable deprecation rate. The logic and steps for this method will be explained in details in Section 4.3.

According to Eq (23), we present the measurement formula for China’s provincial physical capital stock which is as follows:

$$K_{it}=(1-\delta_{it-1})K_{it-1}+I_{it}=(1-\delta_{it-1})K_{it-1}+\frac{I_{it}^{c}}{P_{it}}$$ (24)

Where *K* denotes the physical capital stock at constant prices, *δ* denotes the depreciation rate (variable), *I* denotes the investment flow at constant prices, *I*^*c*^ denotes the investment flow at current prices, *P* denotes the price index, *i* and *t* mark the province and the period. Since this measurement formula takes the difference of the depreciation rate across periods and regions into consideration, it captures the changes in provincial physical capital stock more objectively.

## 4. Indicators and data

From Eq (24), the application of the PIM involves four core indicators: (1) the investment flow *I*; (2) the price index *P*; (3) the depreciation rate *δ*; (4) the initial physical capital stock *K*_0_. In this section, we will discuss how to select or determine the most appropriate indicators for measuring China’s provincial physical capital stock. When it comes to the data, we take the consistency of statistical caliber as a general selection criterion.

### 4.1. Investment flow

The identification for the investment flow mainly involves four indicators: the accumulation [29, 30, 43], the total investment in fixed assets in the whole country (TIFA) [44–46], the newly increased investment in the fixed assets (NIIFA) [47, 48] and the gross fixed capital formation (GFCF) [1, 2, 49].

The accumulation refers to the national income used for enlarged reproduction, nonproductive construction and material reserves during the reference period. Although the use of the accumulation as the investment flow does not need to consider the impact of the depreciation, there are two problems. First, China’s National Bureau of Statistics has stopped publishing the accumulation data since 1995 and the accumulation data from 1994 can only be obtained by measurement [43] which reduces the accuracy of physical capital stock. Second, the accumulation includes the inventory investment which should not be counted in physical capital stock [12, 13, 48].

The TIFA refers to the volume of activities in construction and purchase of the fixed assets of the whole country and related fees during the reference period. Although the TIFA is complete for data and convenient for use [45], it cannot accurately reflect the changes of the investment flow [50]. On the one hand, the TIFA includes the expenditure on the purchase of land, old machinery and old houses, which overestimates the change of physical capital stock. On the other hand, the TIFA only counts the investment projects above a certain scale, which underestimates the changes of physical capital stock. That is, the TIFA only counts the investment projects above 50,000 RMB before 1997, those above 500,000 RMB since 1997 and those above 5 million RMB since 2011.

The NIIFA refers to the value of the fixed assets for production or use which have been built or purchased during the reference period. Although the use of the NIIFA considers the production transformation of the investment, there are two problems: First, the data of the NIIFA is largely missing at the national and provincial levels; Second, the use of the NIIFA is prone to a lower physical capital stock [18, 30].

The GFCF refers to the value of acquisitions less disposals of the fixed assets during the reference period. The NIIFA is the basis for accounting the GFCF. Nonetheless, there are four main differences between the two: (1) The TIFA includes the purchases of land, old equipment and old buildings, but the GFCF does not include these purchases; (2) The TIFA does not include the projects below a certain scale, but the GFCF include these projects; (3) The TIFA does not include the expenditures on mineral exploration, computer software and other intellectual property right products, but the GFCF include these expenditures; (4) TIFA does not include the value added of commercial housing sales of real estate developers, but the GFCF include this part.

Compared with other indicators, the merits of the GFCG on data integrity and content comprehensiveness have made it become a popular selection as the investment flow when measuring physical capital stock. However, although the GFCF objectively reflects the changes of the investment flow, it does not include the subdivision of construction and installment (CI), equipment and instruments (EI) and other items (OI). There are two viewpoints on whether to reflect the subdivision of the GFCF. The first one indicates that it does not matter and we can directly use the GFCF [1, 2, 4]. The second one holds that it does matter and we should divide the GFCF into CI, EI and OI [3, 50, 51]. Although the second viewpoint considers the heterogeneity of the GFCF, the data related to CI, EI and OI (flow data and price data) is seriously missing: At the provincial level, there are all missing from 1952 to 1990 and different degrees of deficiency from 1991 to 2022; At the national level, there are all missing from 1952 to 1980 and different degrees of deficiency from 1981 to 2022.

According to the above explanations, here we clarify the selection criteria for the investment flow: When determining the investment flow, we give preference to the GFCF without the subdivisions in view of the insufficient data on CI, EI and OI. If the data is missing for certain years, we fit or substitute the GFCF with other indicators. To be specific: *China Compendium of Statistics 1949–2008* presents the data of the GFCF of most provinces from 1952 to 2008 and *China Statistical Yearbook* presents the data of the GFCF of all provinces from 2009 to 2017, we directly use it. For the data of the GFCF of Jiangxi province which is missing from 1952 to 1977, we regress the GFCF of Jiangxi province over the capital construction investment of Jiangxi province from 1978 to 2000 and fit the missing data of the GFCF by the regression coefficient and the data of the capital construction investment of Jiangxi province from 1952 to 1977 [1]. For the data of the GFCF of Chongqing municipality which is missing from 1952 to 1995, we regress the GFCF of Chongqing municipality over the GFCF of Sichuan province from 1996 to 2010 and fit the missing data of the GFCF by the regression coefficient and the data of the GFCF of Sichuan province from 1952 to 1995. For the data of the GFCF of Hainan province which is missing from 1952 to 1977, we derive it by the index of GFCF of Guangdong province given that the former used to be a part of the latter. The data of the GFCF of Tibet autonomous region is missing from 1952 to 1991 and we substitute it with the TIFA. *China Statistical Yearbook* does not document the data of the GFCF at the provincial level from 2018 to 2022 and we substitute it with the TIFA. The data is detailed in Appendix A.1 in S1 Appendix.

### 4.2. Price index

The identification for the price index mainly involves three indicators: the price indices for investment in the fixed assets (PIIFA), the implied price indices for investment in the fixed assets (IPIIFA) and the retail price index (RPI).

The PIIFA are the relative figures reflecting the trend and degrees in prices of investment goods and projects in fixed assets during a given period. The PIIFA is the first consideration as the price index when measuring physical capital stock.

The IPIIFA are the relative figures derived by the relationship among the index of GFCG (IGFCF), the GFCG at current price and the IPIIFA to reflect the trend and degrees in prices of investment goods and projects in fixed assets during a given period. The IPIIFA can be substituted for the PIIFA when the latter is unavailable which has become a standard operation to measure the physical capital stock [1, 2, 4, 16, 52]. There are two methods to measure the IPIIFA in view of the data in statistics. One is the base year comparison method whose calculation formula is as follows:

$${IPIIFA}_{t\;year}=\frac{{GFCF}_{t\;year}(at\;current\;price)}{{GFCF}_{base\;year}(at\;current\;price)∙{IGFCF}_{t\;year}}$$ (25)

Where the *IPIIFA*_*t year*_ and *IGFCF*_*t*, *year*_ are expressed in base year so that the *IPIIFA*_*base year*_ and *IGFCF*_*base year*_ are equal to 1. Another is the preceding year comparison method whose calculation formula is as follows:

$${IPIIFA}_{t\;year}=\frac{{GFCF}_{t\;year}(at\;current\;price)}{{GFCF}_{t-1\;year}(at\;current\;price)∙{IGFCF}_{t\;year}}$$ (26)

Where the *IPIIFA*_*t year*_ and *IGFCF*_*t year*_ are expressed in preceding year so that the *IPIIFA*_*t*−1 *year*_ and *IGFCF*_*t*−1 *year*_ are equal to 1.

The RPI are the relative figures reflecting the trend and degree of changes in prices of commodities during a given period. Given that there are few cases where the data of the PIIFA and the IPIIFA are both missing, a little use of the RPI as the price index has little impact on measurement results of physical capital stock and the RPI can be substituted for the PIIFA or the IPIIFA when the latter is unavailable [1].

According to the above explanations, here we clarify the selection criteria for the price index: When determining the price index, we give preference to the PIIFA. If the data of the PIIFA is missing, we substitute it with the IPIIFA. If the series of the IPIIFA is unavailable, we substitute it with the RPI. If the data of RPI is missing, we substitute it with the PIIFA in a certain adjacent province. To be specific: *China Compendium of Statistics 1949–2008* presents the data of the PIIFA of most provinces from 1991 to 2008 and *China Statistical Yearbook* presents the data of the PIIFA of most provinces from 2009 to 2019, we directly use it. For the case that the data of the PIIFA is missing, we obtain the IPIIFA of most provinces from 1952 to 1990 according to Eqs (25) or (26) where the GFCF is presented by the *China Compendium of Statistics 1949–2008* and the IGFCF is presented by *Data of Gross Domestic Product of China*. The series of the IPIIFA of Guangdong province is unavailable from 1952 to 1977, that of Tianjin municipality is unavailable from 1952 to 1988 and that of Tibet autonomous region is unavailable from 1990 to 1992 and 2005 to 2019. For these three cases, we substitute them with the RPI of the corresponding provinces at the same period. The data of the RPI of Hainan province is missing from 1952 to 1990 and we substitute it with the PIIFA of Guangdong province. The data of Tibet autonomous region is missing from 1952 to 1989 and we substitute it with the PIIFA of Qinghai province. *China Statistical Yearbook* does not document the data of the PIIFA at the provincial level from 2020 to 2022 and the IPIIFA of all provinces is unavailable from 2020 to 2022 and we substitute the IPIIFA with the RPI at the same period. The data is detailed in Appendix A.2 in S1 Appendix.

### 4.3. Depreciation rate

The determination for the depreciation rate involves multiple factors and there are seven main approaches:

1. Use the depreciation rate or the depreciation in official statistics. For example, use the basic depreciation rate of fixed assets of state-owned enterprises in China Statistical Yearbook [18, 48] or the depreciation of fixed assets in China Compendium of Statistics [4, 53]. However, there are at least three defects in this approach. First, these two indicators belong to book depreciation which has nothing to do with the relative efficiency of physical capital so that they do not meet the requirements of the PIM [12, 54]. Second, these two indicators adopt the straight-line depreciation method so that they cannot be used to measure the productive physical capital stock. Third, the values of these two indicators are relatively lower [1, 3].
2. Circumvent the depreciation. For example, use the accumulation under the Material Product System (MPS) instead of discussing the issues of the depreciation [14, 30, 43]. Although this approach avoids the impact of the depreciation, it generates the problems of the accumulation as we mentioned in Section 4.1.
3. Use the national income accounting to calculate the depreciation rate or the depreciation. For example, use the national income identity “Depreciation = GDP—National Income + subsidy—Indirect taxes” to calculate the depreciation [55] or the relationship between the depreciation and the physical capital stock in the input-output table to calculate the depreciation rate [56]. However, the depreciation calculated by the former is one in accounting instead of one in economics and that calculated by the latter relies on limited input-output tables.
4. Set the depreciation rate. For example, set the depreciation rate as 5% [57, 58], 6% [36, 59], 10% [60] and so on. However, this approach is quite subjective and lacks adequate explanations.
5. Ignore the depreciation [49]. Neither this treatment meets the requirements of the PIM, nor it meets the characteristics of physical capital.
6. Use the parameter method or econometric method to calculate the depreciation rate. For example, deduce the production function without the variable of physical capital and estimate the depreciation rate [61, 62]. The key of this approach is the production function. If the production function performs poorly, then the parameters including the depreciation rate and the capital output ratio will lose validity.
7. Use the relative efficiency formula to calculate the depreciation rate. If the relative efficiency of a capital good matches the Geometrically Decreasing pattern, then it satisfies Eq (15). Given the relative efficiency *e*_*τ*_ and the service age *τ* of a capital good, the depreciation rate *δ* of that can be calculated by Eq (15). According to the usual approach, the salvage value rate of a capital good can be used to replace the relative efficiency of that at the retirement time [1, 2, 63]. The statutory or expected salvage value rate of fixed assets in China is 3%-5%, which means that the relative efficiency of a capital good at the retirement time is 3%-5%. The depreciation rate of a capital good can be calculated after determining the service life of that. According to China’s statistics, the investment in fixed assets can be divided into construction and installment (CI), equipment and instruments (EI) and other items (OI). When using this method to calculate the depreciation rate, OI can be ignored given that they are dependent on EI and OI [2]. The weighted depreciation rate of physical capital can be calculated after determining the service lives and relative proportion of EI and OI. This method has become a popular choice in the studies on measuring physical capital stock because of its simpleness in principle and convenience in operation.

However, the direct use of this method suffers from two pitfalls. First, different service lives set by this method result in different depreciation rates. According to the relative efficiency formula, there is an inversely proportional relationship between the service life and the depreciation rate. The service lives of CI and EI in previous studies are usually set to 40 and 16 years [1], 38 and 16 years [2], 38 and 20 years [63], or 55 and 16 years [64]. Although these practices provide helpful references, they lack convincing explanations. Only when the service life is reasonable can the depreciation rate be objective. Second, the constant depreciation rate derived by this method cannot reflect the wear of fixed assets and the changes of economic activities. Fixed assets are closely related to economic activities that fixed assets are the material carrier and technical support of economic activities. The depreciation of fixed assets is an important component from the perspective of GDP accounting and an important material basis from the perspective of economic growth. On the one hand, the greater is the depreciation rate of fixed assets, the greater is the wear of fixed assets, the greater is the value of fixed assets transferred to new products, thus the faster is the economic growth rate. On the other hand, the faster is the economic growth rate, the faster is the demand growth rate, the more enterprises increase production (increase the physical wear of fixed assets) or update technology (increase the mental wear of fixed assets) in order to boost profits, thus the greater is the depreciation rate of fixed assets. To some extent, the variations of the depreciation rate of fixed assets are proportional to the fluctuation of economic growth rate. By this method, even though the service life is set reasonably, the resulting constant depreciation rate does not reflect the use of physical capital and the growth of economic activity. Only when the depreciation rate is variable can measurement results of physical capital stock be reasonable.

The second crucial work of this paper is how to solve these two problems. Here we clarify the measurement method of the depreciation rate: When determining the depreciation rate, we use the relative efficiency formula to derive the variable depreciation rate according to the dynamic relationship between the depreciation rate of physical capital and the growth rate of economic activity. The determination criteria for the variable depreciation rate is specifically manifested as the follows: (1) Set the statutory or expected salvage value rate of fixed assets; (2) Set the service lives of CI and EI in 1985 by referring to *Service Life Classification Table for Fixed Assets of State-owned Enterprise* issued by China’s State Council in 1985 (As a matter of fact, up to now, it provides the only statistics on the service lives of different fixed assets and this is why we build the benchmark depreciation rate series of physical capital based on the content in 1985); (3) Calculate the depreciation rates of CI and EI in 1985; (4) Calculate the benchmark depreciation rate of physical capital at the national level weighted by the proportions of CI and EI in the TIFA form 1952 to 2022; (5) Determine the variable depreciation rate of physical capital at the national and provincial levels by adjusting the benchmark depreciation rate of physical capital according to the economic growth rate.

The reason why we adjust the benchmark depreciation rate of physical capital according to the economic growth rate is based on two considerations. One consideration is that why we adjust the benchmark depreciation rate of physical capital. Supposing that the relative efficiency of fixed assets matches the Geometrically Decreasing pattern, we calculate the constant depreciation rate of CI and EI according to Eq (15). By the weighted approach, we derive the benchmark depreciation rate of physical capital. As discussed before, this depreciation rate cannot truly reflect the wear of fixed assets and the consumption of economic activities. If the relative efficiency of fixed assets matches the quasi-Geometrically Decreasing pattern, then we can derive the variable depreciation rate of fixed assets in each period according to Eq (22) and this depreciation rate to some extent can truly reflect the changes of economic operation. Unfortunately, even though the service life is reasonable, it is difficult to determine the relative efficiency distribution and the variable depreciation rate distribution of fixed assets in all periods and therefore it is impossible to determine them in each period. In order not only to reflect the changing economic operation, but also to apply the measurement formula in form of the variable depreciation rate, we need to adjust the benchmark depreciation rate of physical capital. Another consideration is that how we adjust the benchmark depreciation rate of physical capital. We select the economic growth rate which can best reflect the economic changes to adjust the benchmark depreciation rate of physical capital. As discussed before, to some extent, the variations of the depreciation rate of physical capital reflect the fluctuation of economic growth rate. Previous studies have also discovered the positive relationship between the depreciation rate of physical capital and the economic growth rate [25, 65]. Therefore, we believe that there is a positive relationship between the depreciation rate of physical capital and the economic growth rate. The variable depreciation rate of physical capital adjusted by the economic growth rate can dynamically reflect the economic growth situation. To be specific:

We set the statutory or expected salvage value rate of fixed assets to 4% according to *Regulations on Depreciation of Fixed Assets of State-owned Enterprises* issued by China’s State Council in 1985 and previous studies [1, 2]. The service life of fixed assets is expressed by the service lives of CI and EI. CI is composed of houses and buildings. We calculate the average service life of those according to *Service Life Classification Table for Fixed Assets of State-owned Enterprise* and the result is 37.5 years. Based on this result, we believe that the service life of CI is 38 years. EI is composed of general equipment and special equipment. For general equipment, we calculate the average service life of that according to *Service Life Classification Table for Fixed Assets of State-owned Enterprise* and the result is 16.9 years. For special equipment, there are several steps to be explained: (1) Divide special equipment into 13 categories by industry according to *Service Life Classification Table for Fixed Assets of State-owned Enterprise* and *China Statistical Yearbook (1986)*; (2) Calculate the average service life of each category of special equipment according to *Service Life Classification Table for Fixed Assets of State-owned Enterprise*; (3) Calculate the proportions of the industries where 13 categories of special equipment are mainly used in the total output value consisting of industry, construction, transportation and commerce respectively according to *China Statistical Yearbook (1986)*; (4) Calculate the average service life of 13 categories of special equipment weighted by the above proportions and the result is 15.7 years. After deriving the service lives of special equipment and general equipment, we calculate the average service life of those and the result is 16.3 years. Based on this result, we believe that the service life of EI is 16 years. The depreciation rates of CI and EI in 1985 can be calculated respectively by Eq (15) and the results are 8.12% and 18.22%. *China Statistical Yearbook* only presents the data of CI and EI in the capital construction investment at the national level from 1952 to 1980 and the data of CI and EI in the TIFA at the national level from 1981 to 2022. There is no better data in all the statistics. In this case, we regard the proportions of CI and EI in the capital construction investment from 1952 to 1980 as those in the TIFA from 1952 to 1980 [1]. After calculating the proportions of CI and EI in the TIFA from 1952 to 2022, we can derive the benchmark depreciation rate series of physical capital at the national level weighted by the proportions of CI and EI.

The benchmark depreciation rate series of physical capital at the national level from 1952 to 2022 is based on the use intensity and the service life of fixed assets at the national level in 1985, which can only reflect the wear of fixed assets at the national level in 1985. In order to reflect the depreciation of fixed assets at the national and provincial level from 1952 to 2022, we adjust the benchmark depreciation rate of physical capital according to the economic growth rate. Given the relationship between the depreciation rate of physical capital and the economic growth rate, we correlate the variations in the former with the fluctuations in the latter. Taking the deviation between the economic growth rate of different provinces in different years and the national economic growth rate in 1985 as the adjustment coefficient, the calculation formula for the variable depreciation rate of physical capital of each province is as follows:

$$\delta_{it}={\bar{\delta }}_{t}(g_{it}-g_{1985}+1)$$ (27)

Where *δ* denotes the depreciation rate of physical capital, $\bar{\delta }$ denotes the benchmark depreciation rate of physical capital, *g* denotes GDP growth rate, *g*_1985_ denotes GDP growth rate of the whole country in 1985, *i* and *t* mark the region and the period. According to the benchmark depreciation rate of physical capital and GDP index from 1952 to 2022, we derive the variable depreciation rate of physical capital at the national and provincial levels from 1952 to 2022, which to some extent reflects economic reality. The results are detailed in Appendix A.3 in S1 Appendix.

### 4.4. Initial physical capital stock

The measurement for the initial physical capital stock mainly involves four methods: the steady state method (SSM), the summation method (SM), the parameter method (PM) and the capital output ratio method (CORM). The principle of the SSM is that the growth rate of physical capital stock is equal to the growth rate of the investment under steady state condition. The calculation formula for the SSM is as follows:

$$K_{0}=I_{0}\frac{1+\theta }{\delta +\theta }$$ (28)

Where *K*_0_ denotes the initial physical capital stock, *I*_0_ denotes the initial investment flow, *θ* denotes the growth rate of the investment flow and *δ* denotes the depreciation rate of physical capital. Although the SSM is feasible and operational, it relies on the steady state condition which is too strict for the economic performance.

The SM includes two forms: the integral method of investment flow (IMIF) and the convergence method of investment flow (CMIF). The principle of the IMIF is that the initial physical capital stock can be expressed as the sum of the past investment flow. The calculation formula for the IMIF is as follows:

$$K_{0}=\int_{-\infty }^{0}I_{t}d_{t}=\frac{I_{0}}{\theta }$$ (29)

Where *I*_*t*_ = *I*_0_*e*^*θt*^, *I* denotes the investment flow and *t* marks the period. *I*_0_ and *θ* can be estimated by linear regression which is:

$$lnI_{t}=b+\theta t$$ (30)

Where the constant term *b* stands for *lnI*_0_ and *I*_0_ is equal to *e*^*b*^. Although this form of integral method is applicable for measurement [5, 57, 66, 67], there are two problems. First, the growth rate of the investment flow *θ* is not necessarily steady; Second, the investment flow *I* does not exclude the depreciation of physical capital. The former easily leads to unrobust results and the latter easily leads to higher results.

The principle of the CMIF is that the initial physical capital stock can be expressed as the sum of the past investment flow excluding depreciation [12]. The calculation formula for the CMIF is as follows:

$$K_{0}\approx [I_{0}+(1-\delta )I_{-1}+{\left(1-\delta \right)}^{2}I_{-2}+\ldots ]=I_{0}\frac{1+\theta }{\delta +\theta }$$ (31)

Although this form of integral method is also applicable for measurement, there is a flaw just like the IMIF that the growth rate of the investment flow *θ* is not necessarily steady.

The SSM, the IMIF and the CMIF can be transformed into the growth rate method (GRM) under certain conditions. The calculation formula for the GRM is as follows:

$$K_{0}=\frac{I_{0}}{\delta +\theta }$$ (32)

This can be described as the simple SSM from the perspective of the SSM, the IMIF with the depreciation rate from the perspective of the IMIF or the CMIF without the current period from the perspective of the CMIF. The SSM is widely used to measure physical capital stock because of its conciseness.

The principle of the PM is that the elasticity of output with respect to capital can be estimated econometrically based on the production function without the variable of physical capital stock and the initial physical capital stock can be calculated by the elasticity of output with respect to capital [61, 62]. The objective function of the PM is as follows:

$$F(g_{Y_{t}},I_{t-1},L_{t-1},Y_{t-1},g_{L_{t}})=0$$ (33)

Where *Y* denotes the output, *g*_*Y*_ denotes the growth rate of *Y*, *I* denotes the investment, *L* denotes the labor and *g*_*L*_ denotes the growth rate of *L*. Although the PM is feasible and operational, it relies on the production function which brings about the problem as we mentioned in Section 4.3.

The principle of the CORM is that the initial physical capital stock can be calculated by a given capital output ratio [14, 49, 55]. Although the CORM is simple, it raises subjective biases which seriously affect the measurement accuracy.

The third crucial work of this paper is how to overcome or avoid these defects. Here we clarify the measurement method of the initial physical capital stock: When measuring the initial physical capital stock, we adopt a new CORM given that the capital output ratio is relatively steady in the short run when the economic conditions are similar. The calculation formula for this CORM is as follows:

$$K_{t}\approx \frac{Y_{t}I_{t+1}}{Y_{t+1}-(1-\delta_{t})Y_{t}}$$ (34)

Where *K* denotes the physical capital stock, *δ* denotes the depreciation rate. To make *K*_*t*_ is meaningful, *Y*_*t*+1_−(1−*δ*_*t*_)*Y*_*t*_>0 is essential. The initial physical capital stock measured by the capital output ratio can reflect the economic aggregate. To be specific:

Suppose that the capital output ratios in 1952, 1953 and 1954 are approximately equal for the similar economic environment in three years. Let *t* be 1952, 1953 and 1954 respectively. *Y* and *I* are expressed as GDP and the investment flow at 1978 prices respectively, while *δ* is derived by Eq (27). First, we derive *K*_1952_, *K*_1953_ and *K*_1954_ by Eq (34). Second, we derive $K_{1952}^{\prime }$ given *K*_1953_ and $K_{1952}^{\prime \prime }$ given *K*_1954_ by Eq (23). Third, we exclude the abnormal values which contradict with $Y_{t+1}-(1-\delta_{t})Y_{t}>0$ and regard the average of *K*_1952_, $K_{1952}^{\prime }$ and $K_{1952}^{\prime \prime }$ as the initial physical capital stock. Following the above steps, we take 1952 as the base year and derive the initial physical capital stock which can reflect the economic performance. *China Statistical Yearbook* presents the data of GDP and GDP index and we use it to calculate GDP at 1978 prices. The selection criteria for the investment flow and the price index have been discussed in Section 4.1 and Section 4.2 by which we can get the investment flow at 1978 prices. The results are detailed in Appendix A.4 in S1 Appendix.

## 5. Measurement results and temporal-spatial characteristics

According to Eq (24), we derive China’s provincial physical capital stock from 1952 to 2022 at 1978 prices (excluding Hongkong, Macao and Taiwan) after determining the investment flow, the investment price index, the depreciation rate and the initial physical capital stock. As space is limited, we just present China’s provincial physical capital stock in representative years. Table 3 shows these measurement results and all measurement results are detailed in Appendix A.5 in S1 Appendix.

**Table 3:** China’s provincial physical capital stock in representative years (in 100 million RMB).

Province	1952	1960	1970	1978	1980	1990	2000	2010	2015	2020	2022
Beijing	10.0	48.5	56.3	101.6	142.7	956.3	3746.3	11692.7	19210.8	25276.7	26675.4
Tianjin	17.6	35.0	38.1	110.7	131.5	353.7	935.4	4535.1	10457.0	14689.6	16154.7
Hebei	45.7	96.7	115.2	256.3	305.1	697.7	2506.0	10025.8	19301.4	35521.3	43130.0
Shanxi	23.4	94.3	89.8	160.2	172.3	422.6	898.1	4292.6	8545.6	10005.7	10736.9
Neimenggu	31.0	55.1	53.3	83.4	97.9	293.4	967.2	9477.6	21003.2	24987.7	26792.7
Liaoning	144.1	466.8	339.5	304.4	321.8	817.3	1798.6	8123.1	14889.0	14442.8	14117.1
Jinlin	33.0	56.4	60.8	100.2	117.6	317.4	882.5	5824.1	11533.6	16217.8	17989.9
Heilongjiang	30.2	80.4	80.2	140.5	174.8	555.2	1253.1	4214.5	8424.9	12260.6	13830.6
Shanghai	17.8	50.1	59.0	155.5	198.9	791.2	3100.7	8680.3	12518.8	15793.2	16715.4
Jiangsu	48.6	90.1	106.3	199.4	253.0	1466.5	6048.8	25185.1	45049.9	78204.3	92284.0
Zhejiang	4.9	13.8	19.7	34.3	39.6	151.7	729.4	9676.4	18035.9	33263.0	41277.6
Anhui	34.6	78.1	65.5	105.9	114.6	420.7	1186.8	4463.1	9051.2	22266.3	29659.1
Fujian	10.1	38.1	35.2	62.9	82.7	240.4	1009.1	4385.0	9045.9	18533.4	22888.1
Jiangxi	16.2	66.8	92.7	143.4	173.2	404.1	1252.1	5908.1	10869.3	27422.0	37225.8
Shandong	38.9	96.8	122.4	279.8	345.9	1111.5	3218.8	14362.2	26843.0	43893.9	51038.9
Henan	29.3	101.9	123.5	232.9	274.6	766.8	2368.6	12680.9	27555.3	50212.1	60851.3
Hubei	12.7	73.3	98.9	213.8	221.4	513.4	2090.9	7606.4	16065.9	31980.5	39629.4
Hunan	12.6	51.8	74.3	143.7	170.4	394.5	1240.4	5327.9	11190.6	23524.6	30298.7
Guangdong	20.9	65.6	72.8	203.6	246.0	869.1	4365.6	17498.1	33334.4	55171.8	63920.8
Guangxi	24.4	54.7	73.5	127.0	141.9	224.7	694.3	3851.6	8628.8	16503.9	11410.7
Hainan	1.2	3.8	4.2	11.8	13.2	84.1	260.6	724.5	1652.2	2723.6	3128.6
Chongqing	13.0	38.5	65.0	118.6	136.6	285.8	741.6	3322.2	6390.6	13692.1	16983.9
Sichuan	19.9	73.1	125.0	229.5	265.1	558.3	1735.0	6832.2	12957.6	26704.0	33452.9
Guizhou	3.9	25.5	55.5	93.4	104.8	219.3	548.9	2189.8	5257.2	13653.6	16658.6
Yunnan	29.0	106.2	145.3	161.9	177.1	240.7	691.3	2430.6	6045.6	12377.8	15255.7
Xizang	0.4	2.9	5.0	9.0	10.8	29.8	144.7	521.7	1126.9	2302.1	2561.8
Shaanxi	9.7	37.0	58.2	139.9	161.1	442.3	1016.5	4734.5	10050.1	20118.2	24302.6
Gansu	26.5	93.5	103.0	147.6	160.1	284.2	572.3	2278.2	4631.8	7388.4	8818.0
Qinghai	3.5	24.0	25.3	39.6	49.7	94.4	250.7	1072.0	3009.4	5370.3	5814.0
Ningxia	2.6	9.1	20.2	46.5	51.9	92.0	174.5	876.9	2064.0	3083.8	3309.0
Xinjiang	9.7	30.0	38.5	60.8	77.5	243.0	812.0	2555.9	5908.1	9515.2	11249.9

To assess whether our measurement results are reliable, we compare them with those in previous studies. As discussed before, the depreciation rate which we adopt has two characteristics. Compared with the constant depreciation rate, the depreciation rate which we adopt is variable. The depreciation rate is variable in different provinces and is also variable in different years in the same province. Compared with the variable depreciation rate, the depreciation rate which we adopt is adjusted according to the growth rate best reflecting the economic change.

**Fig 1:**
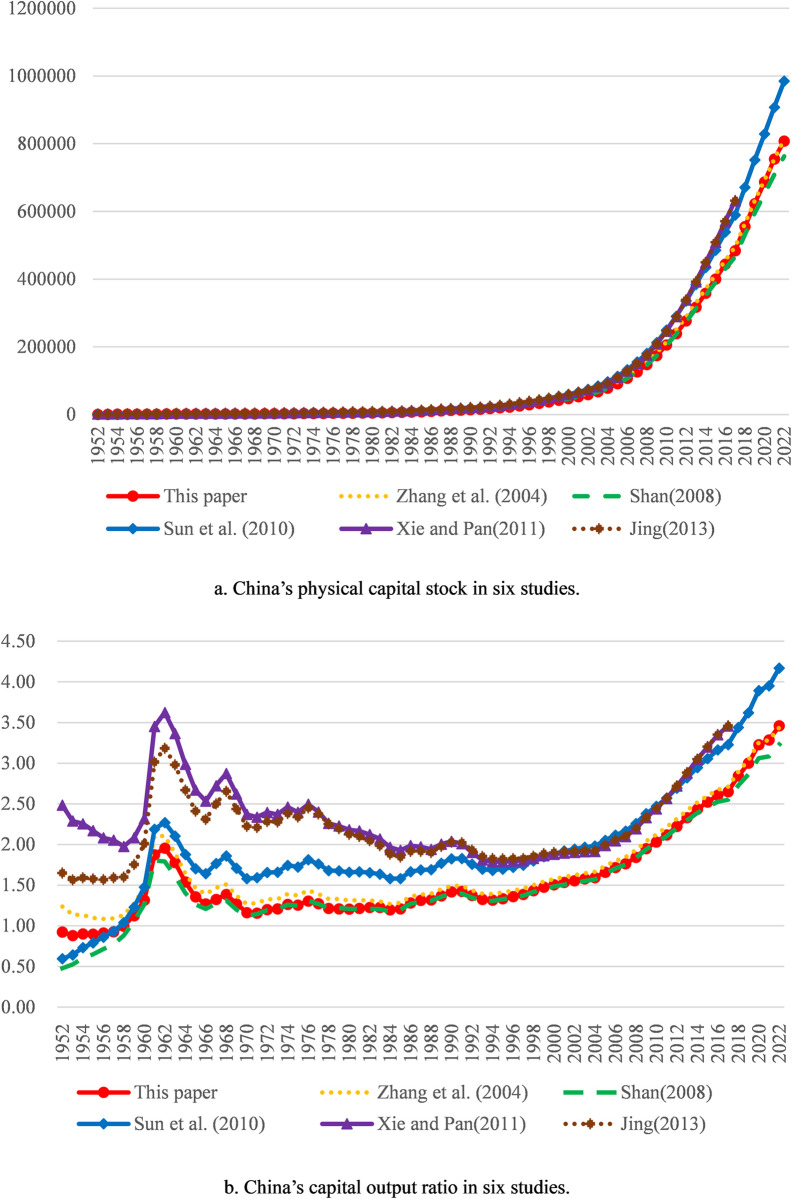
Comparative results for aggregate values of all provinces. Note: Physical capital stock trajectories are expressed in 100 million RMB at 1978 prices; Capital output ratio trajectories are expressed as physical capital stock divided by GDP.

**Fig 2:**
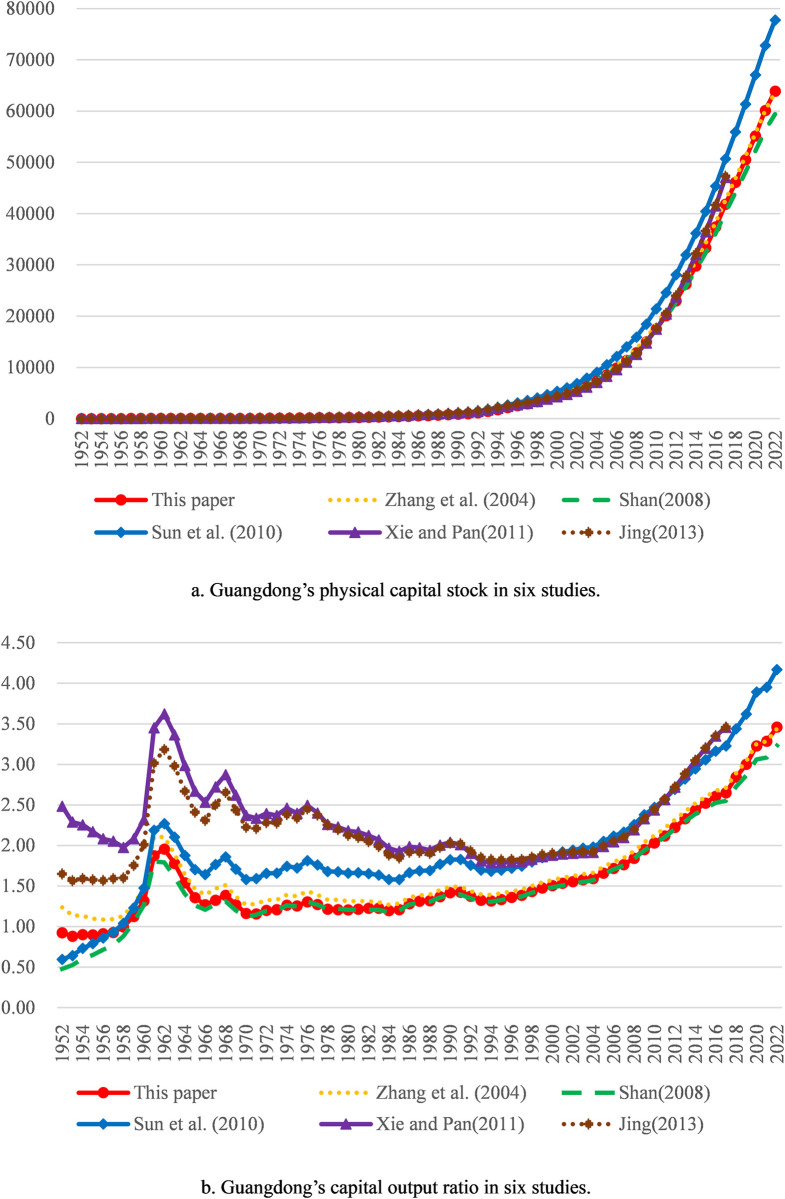
Comparative results for Guangdong. Note: The explanations of physical capital stock trajectories and capital output ratio trajectories are the same as Fig 1.

**Fig 3:**
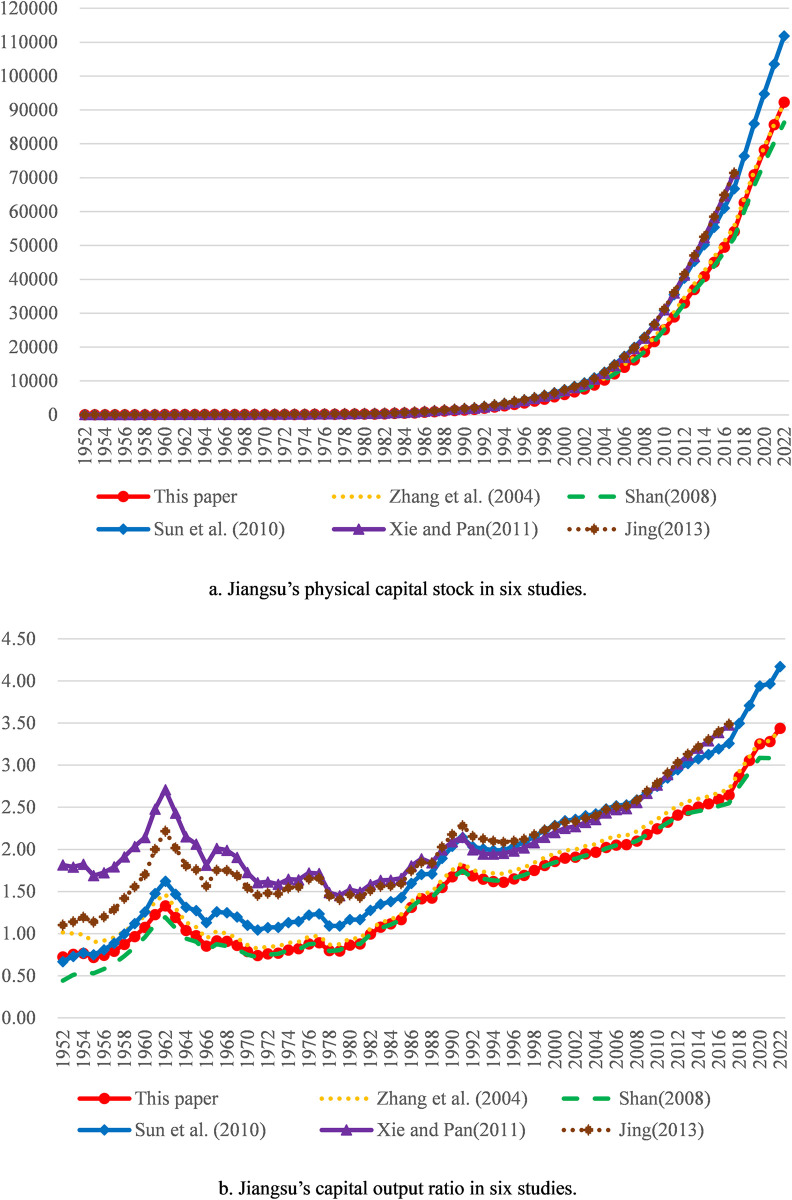
Comparative results for Jiangsu. Note: The explanations of physical capital stock trajectories and capital output ratio trajectories are the same as Fig 1.

**Fig 4:**
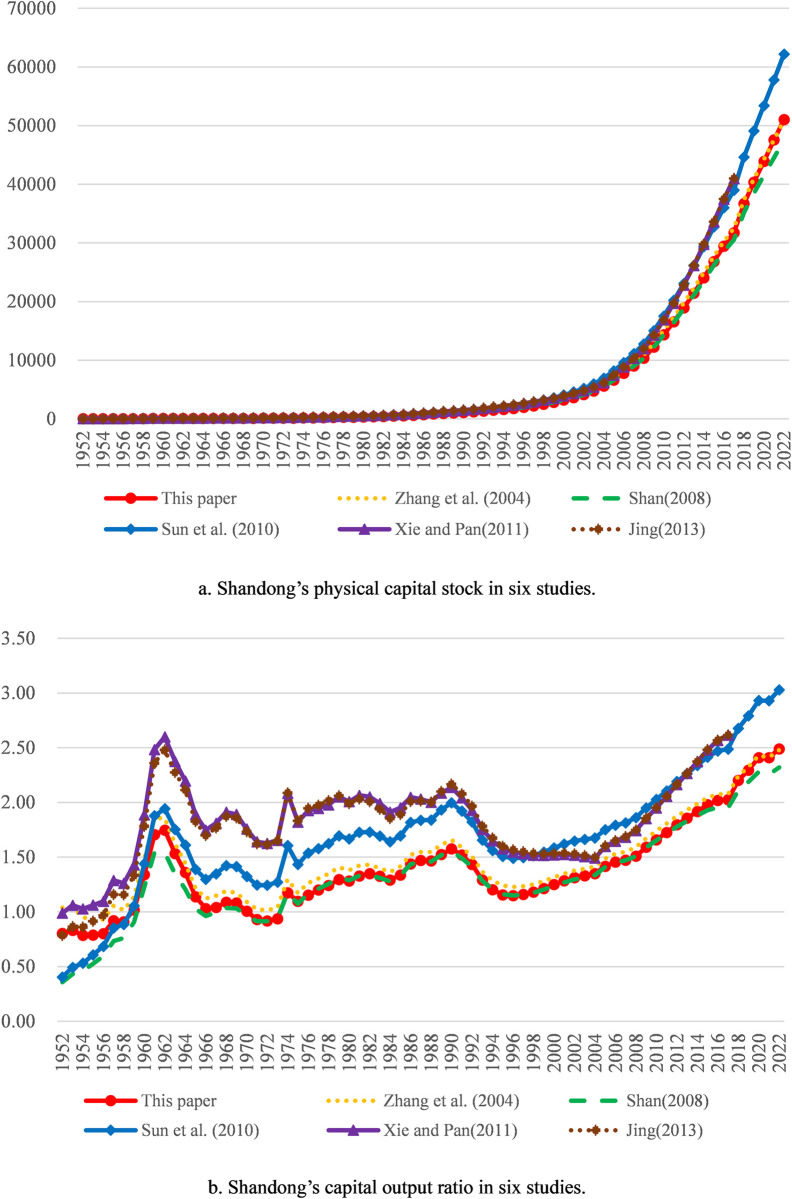
Comparative results for Shandong. Note: The explanations of physical capital stock trajectories and capital output ratio trajectories are the same as Fig 1.

The average depreciation rate adopted by us is 10.58%. Since the depreciation rate has a great impact on measurement results of physical capital stock, the difference of the depreciation rate constitutes the main reason for the difference in measurement results. Our measurement results are slightly less than those derived by the method in Zhang et al. (2004) [1] overall, mainly because the depreciation rate of the latter is relatively small. Our measurement results are slightly larger than those derived by the method in Shan (2008) [2] overall, mainly because the depreciation rate of the latter is relatively big. Our measurement results are less than those derived by the methods in Xie and Pan (2011) [22] and Jing (2013) [4] overall, mainly because the depreciation rate or the depreciation of the latter two are too small. Compared with the measurement results by the method in Sun et al. (2010) [34], ours are larger overall from 1952 to 1956 mainly because of the relatively small initial physical capital stock of the former and lesser overall from 1957 to 2022 mainly because of the relatively small depreciation rate of the former. In general, our measurement results are somewhere between those in other studies. Thus, it can be seen that our measurement results are in the satisfactory range. Besides, compared with the capital output ratios calculated according to the measurement results in the above five studies, ours is also in the satisfactory range. Comparative results for aggregate values of all provinces are shown in Fig 1; comparative results for the three typical provinces with large physical capital stock and gross regional production (Guangdong, Jiangsu and Shandong) are shown in Figs 2 to 4.

The depreciation rate is the key factor causing the differences of the measurement results in these studies. To assess whether our measurement results are advantageous or not, it is not enough just to compare the measurement results, but also to analyze the internal mechanism combined with the measurement results. Compared with the measurement results in the above five studies, the advantage of our measurement results is concentrated in the dynamic variable depreciation rate. By associating the variable depreciation rate with the economic growth rate and then constructing the dynamic variable depreciation rate according to the economic growth rate, we derive the measurement results of China’s provincial physical capital stock which can better reflect the macroeconomic environment. Therefore, our measurement results are closer to the dynamic economic reality and can better describe the size of physical capital stock at the provincial level.

It is important to emphasize that the comparison of our measurement results with those of other studies are only meaningful in particular areas of economic research. We can see that although our measurement results are different from those of other studies in numerical values, they are approximately the same in variation trend. This means that in some econometric analysis, these measurement results do not affect the robustness of physical capital, nor the robustness of other variables. However, when it comes to the research related to physical capital stock, such as capital return share and capital return rate, we have to consider the accuracy and the rationality of measurement results. In this sense, we can flexibly choose how to measure physical capital stock according to our research objectives. If our research has a general requirement for the accuracy of physical capital stock, then we can use a simple method, for example, Zhang et al. (2004) [1] and Shan (2008) [2]. If our research has a high requirement for that, then we have to use or develop a more reasonable method as in this paper.

From the above comparative analysis and mechanism analysis, our measurement results are effective and reliable so that they can reflect economic reality more objectively and meet research needs more reasonable.

According to the measurement results, China’s provincial physical capital stock shows the distinguishing characteristics in the dimensions of time and space. On the one side, the growth rate of physical capital stock at the provincial level is faster. In terms of total quantity, the average annual growth rate of all provinces is 10.91%. The provinces with top five growth rates are Zhejiang (13.82%), Tibet (13.43%), Guizhou (12.73%), Guangdong (12.24%) and Hubei (12.20%), while those with bottom five growth rates are Liaoning (6.89%), Gansu (8.64%), Heilongjiang (9.20%), Shanxi (9.23%) and Yunnan (9.35%). In terms of per capita quantity, the average annual growth of all provinces is 9.38%. The provinces with top five growth rates are Zhejiang (12.05%), Tibet (11.54%), Guizhou (11.19%), Hubei (10.99%) and Hunan (103.63), while those with bottom five growth rates are Liaoning (5.68%), Gansu (7.31%), Heilongjiang (7.58%), Yunnan (8.75%) and Shanxi (7.80%). On the whole, physical capital stock at the provincial level grow rapidly. It is worth noting that compared with the previous decade, the growth rate of physical capital stock at the provincial level has decreases from 2012 to 2022. In terms of total quantity and per capita quantity, the average annual growth rate of all provinces fell by about three-tenths (from 17.07% and 16.05% to 11.75% and 11.59% respectively). This decline has been one of the reasons for the slowdown in the economic growth rate. On the other side, the spatial distribution of physical capital stock at the provincial level is unbalanced. In terms of location quotient for physical capital stock which is expressed with the formula (*K*_*i*_/*P*_*i*_)/(∑_*i*_*K*_*i*_/∑_*i*_*P*_*i*_) where *K*, *L* and *i* denote the physical capital stock, the population and the province respectively [68–70]. The eastern region (the average 1.28) is much larger than the central region (the average 0.81) and the western region (the average 0.84). The coastal region (the average 1.23) is much larger than the inland region (the average 0.84). On the whole, physical capital stock at the provincial level unevenly distributes. Compared with the previous decade, the regional distribution of physical capital stock at the provincial level tends to be optimized from 2012 to 2022. This optimization not only comes from the market mechanism, such as cost differences and resource constraints, which drive the flows of factors and industries to the central and western regions, but also comes from the macro control, such as coordinated regional development strategy and major regional development strategy, which promote the layouts of factors and industries to the central and western regions. It can be seen from the change of location entropy of physical capital stock, the eastern region and the coastal region have decreased (the averages from 1.33 and 1.28 to 1.27 and 1.26 respectively), while the central region and the inland region basically increased (the averages from 0.79 and 0.78 to 0.93 and 0.86 respectively). The western region has been basically unchanged (the average decreased from 0.74 to 0.73).

To some extent, the temporal and spatial characteristics of China’s provincial physical capital stock also reflect the changes of China’s economic growth. Since China’s economy has transitioned to high-quality development, regional economic performance has been improved. For the relatively developed eastern provinces, the growth of physical capital stock slows down and the economic growth depends more on factors improvement such as technological progress. For the relatively underdeveloped central and western provinces, the growth of physical capital stock remains considerable and the economic growth depends more on mechanism improvement such as factor flow. In this sense, we provide a factual basis for discussing the economy shifting to high quality development.

## 6. Discussion

Physical capital stock is the basic indicator of macroeconomic empirical studies and also an important manifestation of macroeconomic activity capacity. How to measure physical capital stock on the basis of developing or improving measurement methods has important reference significance for evaluating the role of physical capital and predicting the performance of macroeconomy. The main work of this paper is to improve the measurement method and make the actual measurement. Below we summarize our findings and discuss possible policy implications and future directions.

### 6.1 Conclusions

On the basis of discussing the fundamental principle and specific application of the PIM, we establish the measurement formula in form of the variable depreciation rate for physical capital stock. Under this framework, we improve the treatment of the depreciation rate and the initial physical capital stock. For the variable depreciation rate, we determine it according to the economic growth rate, which makes it reflect the economic growth. For the initial physical capital stock, we measure it according to the capital output ratio, which makes it reflect the economic aggregate. On the basis of reviewing and identifying four core indicators, we measure China’s provincial physical capital stock from 1952 to 2022 by the PIM, further expand the time span of China’s provincial physical capital stock and provide more reliable data for China’s macroeconomic research. According to the comparative analysis with the measurement results of other studies, we believe that our measurement results are closer to the dynamic economic reality and are more in line with the needs of macroeconomic research. Through these works, taking the variable depreciation rate as the core, we construct a relatively complete measurement system including theoretical explanation and specific process. From analyzing the measurement results, we conclude the temporal and spatial characteristics of China’s provincial physical capital stock, that is, the growth rate of each province remains high and the eastern region (or the coastal region) is much higher than the central and western regions (or the inland region).

### 6.2 Policy implications

According to the temporal and spatial characteristics of China’s provincial physical capital stock, we can gain some policy implications. First, the growth rate of physical capital stock at the provincial level has slowed in spite of remaining high. Given that physical capital is an essential factor of economic growth, policymakers need to take measures to maintain an appropriate increase of the investment in fixed assets, or at least prevent a sharply fall of that, if they want to ensure a benign profile of economic growth. Expanding financial credits, offering tax incentives and increasing government investment should be taken into consideration. Second, the spatial distribution of physical capital stock at the provincial level is unbalanced and the eastern region (or the coastal region) is much higher than the central and western regions (or the inland region). China’s economy is in a stage of high-quality development and coordinated regional development. Since physical capital is an essential factor of economic growth, an important work of policymakers is to plan a rational regional allocation of physical capital stock. For promoting high-quality development, policymakers need to improve market mechanisms, including but not limited to enhancing economic legislation and curbing local protectionism, so as to eliminate artificial obstacles to cross-regional investment and achieve regional optimization of physical capital stock. For promoting coordinated regional development, in addition to improving market mechanisms, policymakers need to adjust investment policies, including but not limited to establishing special funds and providing tax incentives, so as to strengthen the multifarious investment in the central and western regions and consolidate the physical capital condition for economic growth in these regions.

### 6.3 Future directions

Our Study modifies the measurement formula in theory by establishing a measurement formula in form of the variable depreciation rate and strengthens the macroeconomic research in practice by determining the variable depreciation rate and the initial physical capital stock which reflects the economic performance. In addition, our study expands the time span of China’s provincial physical capital stock and provides important data support for discussing China’s economic growth in different stages.

However, it is necessary to point out the potential limitations of this paper. On the one hand, as we explained in Section 3, how to find a variable depreciation rate series is an important part of successfully implementing the measurement formula in form of the variable depreciation rate. For this problem, as we described in Section 4.3, our approach is to connect the rate of economic growth with the depreciation rate of physical capital and obtain the latter by referring to the former. Therefore, when considering the problem of measuring physical capital stock from the framework of this paper, to what extent the depreciation rate obtained by this approach is close to the real depreciation rate is still a difficult question to answer accurately. On the other hand, the theoretical premise of our measurement method is the assumption that the relative efficiency of a capital good matches the quasi-Geometrically Decreasing pattern, which is a modification for the assumption that the relative efficiency of a capital good matches the Geometric Decreasing pattern. Only under these two relative efficiency patterns can the quantity and the price of a capital good form one-to-one correspondence, that is, the age-efficiency function (relative efficiency function) and the age-price function of a capital good have the same trajectory. When abandoning this theoretical premise, measuring physical capital stock is another complex story. We should not only consider wealth stock and productive stock of physical capital, but also analyze age-efficiency functions and retirement functions of capital goods. Although the assumption that the relative efficiency of capital goods matches the Geometric Decreasing pattern has get empirical support [42] and has been adopted by most previous studies, there are still some studies that adopt or mention the assumption that the relative efficiency of a capital good matches the Hyperbolic Decreasing pattern and the retirement of that matches the Bell-shaped pattern [11, 12, 15, 16, 52, 71–76]. Therefore, when evaluating the problem of measuring physical capital stock from a broader framework, which relative efficiency function of a capital good can better describe the efficiency changes of that is still a question that varies from research perspective.

According to the findings and the limitations of this paper, we can continue to conduct research in three directions. First, using the measurement results of this paper, we can continue to study capital return share, capital return rate and capital rental price at the national and provincial levels and continue to analyze the role of physical capital in economic growth at these two levels and further compare these two kinds of studies with previous ones. Given that physical capital is an essential factor of economic growth, we can apply the measurement results of this paper widely in the topics involving economic growth. Second, for the potential limitation of the accuracy of the depreciation rate of physical capital in this paper, we can find other appropriate indicators to measure it, for example, find more appropriate economic indicators for correction, or use other methods to estimate it, for example, establish perfecter econometric model for estimation. Third, for the potential limitation of the goodness of the relative efficiency function of a capital good, we can draw on previous studies to measure physical capital stock when the relative efficiency of a capital good matches different pattens.

## Supporting information

S1 Appendix (DOCX)
