# Supplementary material for: The measurement of China’s provincial physical capital stock based on the improved method and indicators
Source: PLoS One. 2024 Aug 13;19(8):e0307946. doi: 10.1371/journal.pone.0307946 (PMC11321584; doi:10.1371/journal.pone.0307946)
Supplement: S1 Appendix — (DOCX) [file pone.0307946.s001.docx]

**Appendix**

**Appendix A1**

**The Investment Flow (100 million RMB)**

| **Province** | **1952** | **1953** | **1954** | **1955** | **1956** | **1957** | **1958** | **1959** | **1960** | **1961** | **1962** |
| --- | --- | --- | --- | --- | --- | --- | --- | --- | --- | --- | --- |
| Beijing | 0.79 | 5.36 | 8.19 | 5.25 | 7.71 | 7.10 | 11.80 | 17.70 | 18.40 | 8.70 | 0.70 |
| Tianjin | 1.32 | 2.03 | 2.58 | 1.78 | 2.59 | 3.07 | 7.51 | 8.22 | 9.65 | 3.25 | 2.38 |
| Hebei | 5.22 | 6.16 | 6.17 | 7.76 | 7.98 | 10.43 | 22.12 | 23.72 | 25.64 | 10.16 | 4.99 |
| Shanxi | 2.20 | 3.00 | 4.60 | 4.90 | 10.20 | 10.70 | 21.10 | 22.00 | 24.50 | 8.40 | 4.70 |
| Neimenggu | 0.90 | 1.50 | 2.40 | 1.70 | 3.30 | 3.40 | 12.20 | 13.00 | 16.80 | 3.70 | 2.80 |
| Liaoning | 8.60 | 10.80 | 14.20 | 11.10 | 12.80 | 12.70 | 26.80 | 34.10 | 39.60 | 7.60 | 5.80 |
| Jinlin | 1.90 | 3.20 | 5.40 | 5.60 | 6.50 | 4.30 | 8.40 | 10.70 | 11.10 | 5.10 | 3.90 |
| Heilongjiang | 4.20 | 6.00 | 7.50 | 7.60 | 9.00 | 8.10 | 15.80 | 19.20 | 24.10 | 8.00 | 6.30 |
| Shanghai | 2.30 | 3.90 | 3.50 | 3.80 | 4.80 | 5.60 | 12.30 | 16.70 | 18.40 | 7.90 | 4.40 |
| Jiangsu | 5.10 | 7.90 | 4.80 | 5.90 | 7.70 | 8.80 | 12.50 | 15.60 | 21.20 | 11.20 | 9.30 |
| Zhejiang | 0.50 | 0.70 | 0.70 | 1.10 | 1.20 | 0.90 | 1.30 | 1.40 | 1.80 | 1.50 | 1.50 |
| Anhui | 2.40 | 3.40 | 3.60 | 5.70 | 5.50 | 5.80 | 14.30 | 16.20 | 15.20 | 4.80 | 0.40 |
| Fujian | 0.85 | 0.99 | 1.75 | 2.52 | 6.74 | 3.11 | 7.06 | 8.85 | 10.31 | 3.07 | 2.11 |
| Jiangxi | 1.89 | 3.01 | 4.55 | 3.62 | 7.47 | 5.87 | 15.23 | 22.22 | 29.30 | 11.61 | 5.74 |
| Shandong | 5.70 | 6.90 | 8.30 | 11.20 | 14.00 | 14.40 | 15.40 | 19.40 | 22.40 | 9.20 | 8.60 |
| Henan | 4.90 | 3.55 | 4.53 | 8.14 | 10.16 | 11.17 | 22.91 | 29.27 | 32.64 | 11.07 | 8.24 |
| Hubei | 1.66 | 3.21 | 3.69 | 4.44 | 7.23 | 9.53 | 14.94 | 16.57 | 20.13 | 4.73 | 4.35 |
| Hunan | 1.24 | 1.93 | 2.41 | 1.93 | 3.28 | 3.81 | 14.06 | 16.43 | 17.98 | 4.62 | 2.73 |
| Guangdong | 2.21 | 4.51 | 3.72 | 3.44 | 5.04 | 5.42 | 15.07 | 15.41 | 15.98 | 5.36 | 2.52 |
| Guangxi | 1.99 | 2.89 | 3.76 | 3.78 | 4.66 | 3.15 | 5.96 | 9.17 | 7.78 | 3.57 | 2.90 |
| Hainan |  |  |  |  |  |  |  |  |  |  |  |
| Chongqing | 0.89 | 1.31 | 1.70 | 2.44 | 3.32 | 5.05 | 6.40 | 4.57 | 2.56 | 2.45 | 2.05 |
| Sichuan | 1.73 | 2.56 | 3.33 | 4.76 | 6.49 | 9.86 | 12.51 | 8.93 | 5.00 | 4.79 | 4.00 |
| Guizhou | 0.31 | 0.70 | 1.43 | 0.81 | 2.07 | 2.07 | 8.37 | 9.85 | 9.94 | 1.65 | 0.63 |
| Yunnan | 0.84 | 1.61 | 1.81 | 1.92 | 2.85 | 2.92 | 8.15 | 10.30 | 11.83 | 4.07 | 1.67 |
| Xizang | 0.00 | 0.03 | 0.03 | 0.05 | 0.71 | 0.24 | 0.08 | 0.29 | 0.99 | 0.32 | 0.24 |
| Shaanxi | 0.70 | 2.00 | 2.60 | 3.10 | 6.90 | 5.20 | 8.00 | 12.90 | 13.70 | 2.90 | 1.20 |
| Gansu | 4.82 | 5.98 | 6.02 | 6.37 | 8.05 | 9.54 | 10.28 | 13.53 | 14.74 | 11.35 | 10.53 |
| Qinghai | 0.33 | 0.43 | 0.74 | 1.20 | 3.70 | 2.12 | 1.72 | 6.11 | 5.37 | 1.19 | 0.60 |
| Ningxia | 0.37 | 0.39 | 0.50 | 0.51 | 0.87 | 0.83 | 1.75 | 2.32 | 2.50 | 1.24 | 0.77 |
| Xinjiang | 1.95 | 1.65 | 2.08 | 3.28 | 3.60 | 3.72 | 4.91 | 6.56 | 8.92 | 3.95 | 2.34 |
| **Province** | **1963** | **1964** | **1965** | **1966** | **1967** | **1968** | **1969** | **1970** | **1971** | **1972** | **1973** |
| Beijing | 2.30 | 6.20 | 8.30 | 9.00 | 6.30 | 5.90 | 8.70 | 7.10 | 9.80 | 9.90 | 14.40 |
| Tianjin | 3.30 | 4.59 | 3.73 | 4.92 | 2.22 | 2.72 | 4.98 | 7.89 | 8.40 | 10.22 | 15.27 |
| Hebei | 7.49 | 9.38 | 11.92 | 12.39 | 12.52 | 11.76 | 14.66 | 18.38 | 23.05 | 23.63 | 24.97 |
| Shanxi | 5.90 | 8.20 | 10.00 | 11.70 | 7.50 | 5.70 | 8.50 | 15.80 | 20.90 | 20.70 | 20.50 |
| Neimenggu | 4.20 | 5.90 | 7.70 | 7.30 | 3.50 | 4.20 | 4.20 | 7.30 | 8.10 | 7.30 | 8.40 |
| Liaoning | 14.80 | 19.10 | 20.70 | 16.40 | 12.00 | 12.30 | 14.80 | 17.40 | 21.80 | 24.70 | 29.30 |
| Jinlin | 5.20 | 7.00 | 7.50 | 6.90 | 5.40 | 5.30 | 6.50 | 8.90 | 11.60 | 11.60 | 13.40 |
| Heilongjiang | 10.00 | 12.50 | 12.10 | 10.20 | 8.10 | 7.40 | 10.40 | 12.20 | 12.10 | 14.20 | 16.40 |
| Shanghai | 5.80 | 7.90 | 8.30 | 7.90 | 5.30 | 5.20 | 8.10 | 11.50 | 12.70 | 14.60 | 17.70 |
| Jiangsu | 10.20 | 11.60 | 11.90 | 12.30 | 10.20 | 9.90 | 11.50 | 15.50 | 19.20 | 22.00 | 24.00 |
| Zhejiang | 1.50 | 1.20 | 1.30 | 1.50 | 1.50 | 1.30 | 1.40 | 1.70 | 2.00 | 2.80 | 3.20 |
| Anhui | 2.40 | 5.40 | 7.00 | 6.70 | 4.40 | 4.40 | 5.90 | 8.90 | 11.70 | 12.60 | 13.90 |
| Fujian | 2.74 | 4.63 | 4.79 | 4.82 | 1.50 | 1.06 | 1.42 | 7.24 | 10.96 | 10.80 | 9.76 |
| Jiangxi | 8.56 | 9.65 | 11.00 | 12.70 | 8.88 | 9.43 | 16.93 | 25.68 | 24.46 | 22.35 | 22.12 |
| Shandong | 7.60 | 9.00 | 14.20 | 17.50 | 15.80 | 14.40 | 19.20 | 22.50 | 22.90 | 23.30 | 24.90 |
| Henan | 8.76 | 10.36 | 12.21 | 16.53 | 11.89 | 9.06 | 15.57 | 26.05 | 25.60 | 25.81 | 29.36 |
| Hubei | 4.46 | 4.67 | 5.88 | 10.21 | 8.79 | 5.39 | 10.68 | 28.82 | 27.91 | 23.24 | 18.96 |
| Hunan | 3.91 | 8.46 | 9.39 | 11.20 | 9.41 | 7.52 | 10.81 | 18.54 | 22.12 | 18.23 | 19.00 |
| Guangdong | 4.94 | 7.74 | 7.94 | 6.68 | 4.17 | 4.25 | 9.51 | 18.96 | 22.04 | 19.38 | 24.61 |
| Guangxi | 4.01 | 4.67 | 6.44 | 8.94 | 5.45 | 6.66 | 10.22 | 10.74 | 12.41 | 12.68 | 14.98 |
| Hainan |  |  |  |  |  |  |  |  |  |  |  |
| Chongqing | 2.12 | 3.20 | 5.34 | 5.28 | 5.45 | 3.32 | 4.04 | 5.60 | 7.49 | 7.68 | 8.25 |
| Sichuan | 4.15 | 6.25 | 10.43 | 10.31 | 10.65 | 6.48 | 7.90 | 10.95 | 14.64 | 15.00 | 16.11 |
| Guizhou | 2.32 | 5.83 | 13.05 | 15.29 | 8.88 | 7.23 | 9.34 | 18.00 | 23.89 | 16.91 | 11.53 |
| Yunnan | 2.66 | 5.09 | 8.97 | 11.45 | 6.94 | 7.07 | 10.21 | 13.19 | 12.65 | 13.31 | 14.44 |
| Xizang | 0.21 | 0.32 | 0.54 | 0.79 | 0.78 | 0.21 | 0.39 | 0.57 | 0.89 | 0.91 | 0.98 |
| Shaanxi | 2.30 | 5.50 | 7.10 | 11.40 | 6.20 | 4.90 | 10.80 | 18.40 | 24.30 | 23.00 | 20.80 |
| Gansu | 9.38 | 11.73 | 12.04 | 12.31 | 11.09 | 11.85 | 13.03 | 15.05 | 16.67 | 17.31 | 19.25 |
| Qinghai | 0.65 | 0.96 | 1.49 | 2.60 | 1.72 | 1.67 | 2.70 | 3.32 | 3.07 | 6.37 | 2.92 |
| Ningxia | 0.92 | 1.72 | 2.25 | 3.00 | 3.07 | 2.01 | 2.86 | 4.41 | 5.38 | 5.24 | 5.51 |
| Xinjiang | 3.46 | 4.86 | 6.03 | 5.41 | 4.01 | 2.91 | 3.56 | 5.07 | 5.82 | 5.47 | 6.22 |
| **Province** | **1974** | **1975** | **1976** | **1977** | **1978** | **1979** | **1980** | **1981** | **1982** | **1983** | **1984** |
| Beijing | 19.00 | 16.80 | 11.60 | 14.20 | 24.84 | 29.10 | 36.43 | 40.13 | 42.29 | 56.15 | 72.51 |
| Tianjin | 19.98 | 20.80 | 17.38 | 20.18 | 18.55 | 23.91 | 25.56 | 25.00 | 32.36 | 38.27 | 48.67 |
| Hebei | 28.95 | 38.15 | 37.71 | 40.53 | 51.39 | 58.37 | 51.86 | 45.44 | 65.12 | 71.35 | 82.12 |
| Shanxi | 19.30 | 21.10 | 19.60 | 24.70 | 27.40 | 22.50 | 27.30 | 24.70 | 33.50 | 43.60 | 66.90 |
| Neimenggu | 9.80 | 10.60 | 11.70 | 12.80 | 16.60 | 17.70 | 15.80 | 15.50 | 20.90 | 29.70 | 40.90 |
| Liaoning | 32.00 | 33.20 | 30.50 | 30.60 | 33.60 | 45.40 | 42.90 | 43.10 | 63.00 | 71.60 | 92.60 |
| Jinlin | 13.70 | 15.40 | 12.40 | 11.70 | 18.20 | 19.50 | 20.80 | 21.00 | 27.70 | 29.30 | 40.90 |
| Heilongjiang | 18.00 | 20.10 | 21.80 | 23.50 | 26.90 | 28.60 | 38.00 | 46.60 | 62.10 | 70.90 | 87.00 |
| Shanghai | 23.90 | 34.20 | 25.70 | 19.70 | 31.70 | 39.10 | 45.60 | 55.10 | 70.50 | 74.50 | 90.70 |
| Jiangsu | 22.20 | 25.90 | 28.40 | 29.70 | 40.40 | 46.30 | 58.30 | 56.80 | 95.70 | 105.50 | 131.40 |
| Zhejiang | 3.50 | 3.90 | 4.00 | 4.50 | 5.60 | 9.10 | 11.50 | 13.00 | 13.60 | 15.90 | 22.00 |
| Anhui | 14.60 | 14.80 | 15.50 | 15.50 | 15.90 | 16.10 | 18.20 | 15.60 | 31.90 | 44.30 | 65.50 |
| Fujian | 8.66 | 7.35 | 5.31 | 5.02 | 13.25 | 16.35 | 19.70 | 19.64 | 23.36 | 28.71 | 33.51 |
| Jiangxi | 19.14 | 18.88 | 16.45 | 17.86 | 29.70 | 33.90 | 31.30 | 28.60 | 32.30 | 38.70 | 44.80 |
| Shandong | 23.90 | 43.00 | 46.10 | 55.70 | 62.30 | 65.50 | 71.30 | 82.00 | 102.30 | 121.10 | 153.90 |
| Henan | 33.18 | 35.85 | 28.47 | 38.57 | 40.66 | 46.37 | 57.70 | 55.20 | 66.10 | 88.14 | 101.87 |
| Hubei | 21.22 | 31.62 | 34.78 | 40.35 | 31.40 | 29.49 | 33.19 | 31.31 | 45.60 | 53.01 | 74.00 |
| Hunan | 19.32 | 21.31 | 19.33 | 17.83 | 27.68 | 29.81 | 32.76 | 27.95 | 34.07 | 43.89 | 42.15 |
| Guangdong | 29.74 | 37.76 | 36.49 | 34.06 | 37.93 | 41.81 | 57.15 | 73.39 | 94.64 | 96.80 | 133.04 |
| Guangxi | 14.06 | 18.00 | 18.00 | 19.36 | 20.89 | 20.57 | 26.76 | 26.97 | 19.11 | 23.30 | 34.42 |
| Hainan |  |  |  |  | 2.20 | 2.20 | 2.30 | 3.40 | 6.90 | 6.40 | 10.40 |
| Chongqing | 8.90 | 9.93 | 11.61 | 19.23 | 23.01 | 28.09 | 30.89 | 26.46 | 30.88 | 35.44 | 44.49 |
| Sichuan | 17.39 | 19.41 | 22.69 | 37.57 | 44.96 | 54.89 | 60.36 | 51.70 | 60.34 | 69.25 | 86.93 |
| Guizhou | 8.78 | 11.21 | 9.40 | 11.27 | 17.98 | 18.13 | 17.53 | 17.34 | 19.62 | 21.58 | 30.05 |
| Yunnan | 14.21 | 15.45 | 12.55 | 16.52 | 20.55 | 27.64 | 28.70 | 26.09 | 29.01 | 29.63 | 41.97 |
| Xizang | 1.05 | 1.09 | 1.09 | 1.31 | 1.85 | 2.08 | 1.81 | 1.22 | 1.56 | 1.99 | 4.79 |
| Shaanxi | 18.70 | 19.60 | 16.40 | 19.10 | 23.20 | 26.00 | 26.40 | 27.40 | 33.50 | 39.90 | 46.20 |
| Gansu | 21.93 | 23.96 | 23.01 | 22.76 | 28.16 | 27.42 | 22.18 | 19.79 | 21.87 | 26.84 | 29.65 |
| Qinghai | 3.72 | 3.58 | 3.95 | 5.91 | 8.90 | 11.15 | 7.65 | 6.58 | 10.20 | 9.97 | 10.37 |
| Ningxia | 5.51 | 7.48 | 6.50 | 7.98 | 8.00 | 8.80 | 7.50 | 5.50 | 7.80 | 9.00 | 10.70 |
| Xinjiang | 7.08 | 7.53 | 7.11 | 9.48 | 13.25 | 14.53 | 18.14 | 19.35 | 25.50 | 30.30 | 36.54 |
| **Province** | **1985** | **1986** | **1987** | **1988** | **1989** | **1990** | **1991** | **1992** | **1993** | **1994** | **1995** |
| Beijing | 102.72 | 115.96 | 148.59 | 177.68 | 151.94 | 195.02 | 208.78 | 289.01 | 445.53 | 703.76 | 912.03 |
| Tianjin | 67.60 | 75.89 | 81.63 | 96.85 | 95.43 | 98.03 | 144.19 | 185.12 | 246.16 | 345.46 | 417.95 |
| Hebei | 116.38 | 127.62 | 137.63 | 195.17 | 180.90 | 204.02 | 254.27 | 390.02 | 530.37 | 704.87 | 955.21 |
| Shanxi | 88.90 | 94.00 | 103.00 | 104.40 | 104.70 | 117.20 | 144.40 | 175.50 | 242.60 | 290.90 | 314.10 |
| Neimenggu | 50.90 | 47.60 | 53.30 | 72.10 | 70.70 | 70.80 | 100.70 | 149.20 | 219.40 | 250.20 | 273.20 |
| Liaoning | 130.90 | 158.60 | 210.20 | 260.30 | 241.90 | 262.40 | 323.70 | 405.20 | 731.60 | 888.00 | 885.00 |
| Jinlin | 62.40 | 63.40 | 76.70 | 92.90 | 81.70 | 94.00 | 117.40 | 152.60 | 252.90 | 320.50 | 358.10 |
| Heilongjiang | 109.90 | 121.60 | 138.60 | 159.60 | 157.00 | 169.30 | 196.20 | 246.20 | 335.00 | 435.70 | 558.80 |
| Shanghai | 116.70 | 144.40 | 183.80 | 236.90 | 232.20 | 248.50 | 276.10 | 382.00 | 624.30 | 996.50 | 1380.90 |
| Jiangsu | 193.30 | 240.40 | 303.50 | 390.50 | 336.20 | 374.10 | 462.00 | 747.30 | 1201.40 | 1435.00 | 1756.90 |
| Zhejiang | 26.90 | 30.50 | 39.20 | 50.40 | 57.50 | 74.20 | 90.50 | 108.40 | 137.70 | 198.30 | 259.30 |
| Anhui | 86.30 | 103.00 | 114.60 | 127.10 | 123.30 | 138.20 | 150.90 | 200.30 | 325.20 | 456.30 | 621.20 |
| Fujian | 45.16 | 61.19 | 73.76 | 84.36 | 90.45 | 108.02 | 143.21 | 198.45 | 348.16 | 549.29 | 707.12 |
| Jiangxi | 52.10 | 61.60 | 65.80 | 71.70 | 79.10 | 78.90 | 86.60 | 136.90 | 222.50 | 282.80 | 325.60 |
| Shandong | 195.40 | 230.50 | 293.40 | 335.10 | 333.30 | 412.60 | 555.80 | 758.30 | 1023.20 | 1225.50 | 1473.80 |
| Henan | 138.19 | 158.84 | 174.20 | 222.33 | 209.62 | 225.73 | 279.06 | 346.67 | 485.98 | 670.84 | 877.41 |
| Hubei | 96.71 | 104.74 | 130.98 | 149.87 | 115.50 | 147.13 | 176.32 | 235.63 | 377.95 | 571.68 | 778.25 |
| Hunan | 58.14 | 75.57 | 91.79 | 114.73 | 83.19 | 122.82 | 160.49 | 228.29 | 330.54 | 422.61 | 534.12 |
| Guangdong | 163.84 | 182.15 | 197.01 | 286.00 | 266.68 | 336.61 | 396.49 | 683.66 | 1110.69 | 1375.09 | 1819.17 |
| Guangxi | 42.22 | 55.21 | 62.72 | 75.63 | 70.81 | 72.60 | 99.38 | 150.11 | 278.07 | 382.59 | 423.37 |
| Hainan | 16.40 | 18.60 | 20.90 | 22.90 | 29.10 | 42.10 | 50.90 | 105.10 | 145.40 | 199.30 | 193.60 |
| Chongqing | 53.01 | 57.10 | 65.67 | 75.50 | 77.38 | 97.92 | 119.37 | 153.58 | 233.99 | 297.33 | 381.59 |
| Sichuan | 103.58 | 111.56 | 128.31 | 147.51 | 151.19 | 191.31 | 233.22 | 300.06 | 457.17 | 580.93 | 745.56 |
| Guizhou | 40.06 | 45.24 | 50.20 | 56.96 | 54.13 | 64.34 | 74.23 | 92.25 | 122.02 | 150.63 | 182.70 |
| Yunnan | 50.07 | 55.66 | 59.75 | 77.51 | 79.90 | 89.25 | 150.37 | 208.41 | 288.95 | 337.09 | 399.91 |
| Xizang | 7.49 | 5.35 | 5.30 | 5.81 | 6.69 | 7.61 | 10.57 | 13.33 | 12.94 | 17.87 | 29.72 |
| Shaanxi | 63.90 | 72.00 | 89.60 | 102.70 | 106.60 | 120.80 | 140.60 | 165.20 | 247.40 | 300.30 | 358.80 |
| Gansu | 39.17 | 45.83 | 52.19 | 53.64 | 59.78 | 67.19 | 76.47 | 85.73 | 98.42 | 116.57 | 146.35 |
| Qinghai | 15.59 | 15.30 | 18.76 | 21.34 | 16.23 | 20.44 | 23.91 | 29.04 | 41.03 | 47.76 | 57.48 |
| Ningxia | 17.10 | 19.40 | 22.00 | 22.90 | 23.00 | 25.50 | 31.70 | 38.40 | 52.70 | 62.00 | 70.10 |
| Xinjiang | 46.78 | 51.94 | 55.78 | 75.69 | 86.73 | 91.50 | 124.77 | 183.46 | 264.90 | 376.95 | 377.91 |
| **Province** | **1996** | **1997** | **1998** | **1999** | **2000** | **2001** | **2002** | **2003** | **2004** | **2005** | **2006** |
| Beijing | 949.61 | 1040.05 | 1249.36 | 1264.54 | 1400.36 | 1602.06 | 1951.88 | 2437.88 | 2844.29 | 3204.65 | 3551.17 |
| Tianjin | 491.90 | 556.43 | 640.15 | 631.92 | 695.10 | 805.34 | 926.65 | 1180.54 | 1446.49 | 1753.92 | 2122.35 |
| Hebei | 1240.62 | 1486.07 | 1636.10 | 1790.36 | 1846.29 | 1947.60 | 2058.08 | 2512.48 | 3279.73 | 4239.12 | 5094.34 |
| Shanxi | 351.10 | 434.10 | 562.00 | 605.40 | 657.60 | 743.00 | 876.70 | 1113.20 | 1479.50 | 1897.40 | 2345.60 |
| Neimenggu | 276.00 | 319.00 | 353.40 | 390.00 | 439.40 | 510.00 | 729.40 | 1228.30 | 1817.70 | 2685.20 | 3353.90 |
| Liaoning | 920.70 | 976.50 | 1069.60 | 1115.80 | 1293.90 | 1444.20 | 1627.60 | 2102.10 | 3025.10 | 3706.00 | 4755.80 |
| Jinlin | 412.80 | 386.40 | 442.30 | 525.00 | 627.80 | 699.70 | 824.20 | 998.10 | 1291.90 | 1802.40 | 2804.30 |
| Heilongjiang | 653.60 | 724.40 | 865.30 | 859.10 | 922.40 | 1046.80 | 1152.90 | 1261.30 | 1509.90 | 1788.70 | 2232.00 |
| Shanghai | 1711.50 | 1795.80 | 1804.80 | 1801.60 | 1933.00 | 2100.00 | 2366.10 | 2643.10 | 3239.00 | 3743.40 | 4272.90 |
| Jiangsu | 2062.20 | 2296.00 | 2642.30 | 2842.70 | 3225.40 | 3543.20 | 3994.20 | 5480.80 | 6972.70 | 8739.70 | 10021.40 |
| Zhejiang | 310.60 | 385.30 | 494.10 | 627.50 | 816.80 | 976.30 | 1187.80 | 1284.60 | 1506.90 | 6269.20 | 7065.70 |
| Anhui | 713.80 | 783.60 | 828.20 | 845.90 | 936.40 | 1022.50 | 1146.10 | 1379.00 | 1903.10 | 2224.30 | 2665.00 |
| Fujian | 849.23 | 960.86 | 1116.58 | 1150.19 | 1216.91 | 1269.93 | 1383.54 | 1672.63 | 2100.48 | 2654.95 | 3310.15 |
| Jiangxi | 395.90 | 477.30 | 520.80 | 552.70 | 605.50 | 696.70 | 931.80 | 1269.90 | 1633.50 | 1922.10 | 2282.40 |
| Shandong | 1765.40 | 2027.80 | 2324.00 | 2632.50 | 3159.00 | 3518.30 | 4192.60 | 5180.80 | 6896.10 | 8798.80 | 10408.80 |
| Henan | 1079.31 | 1254.89 | 1413.55 | 1469.87 | 1641.43 | 1790.76 | 2018.58 | 2431.76 | 3217.10 | 4506.75 | 6001.50 |
| Hubei | 985.03 | 1102.10 | 1238.90 | 1320.10 | 1451.85 | 1610.93 | 1699.78 | 1875.78 | 2325.87 | 2804.01 | 3555.21 |
| Hunan | 678.32 | 725.73 | 846.37 | 956.10 | 1082.00 | 1233.17 | 1380.89 | 1613.12 | 1978.31 | 2569.24 | 3125.38 |
| Guangdong | 1919.41 | 2079.15 | 2473.82 | 2870.40 | 3093.82 | 3447.52 | 4023.73 | 4986.53 | 5957.86 | 7407.54 | 8465.26 |
| Guangxi | 483.57 | 487.29 | 573.10 | 629.07 | 670.65 | 735.56 | 842.72 | 990.71 | 1296.55 | 1699.04 | 2201.15 |
| Hainan | 168.30 | 163.40 | 174.70 | 189.30 | 198.00 | 208.20 | 227.50 | 265.60 | 311.10 | 363.40 | 423.90 |
| Chongqing | 316.10 | 395.60 | 516.60 | 560.00 | 628.00 | 751.70 | 918.90 | 1220.60 | 1515.60 | 1862.70 | 2138.20 |
| Sichuan | 881.35 | 1015.48 | 1175.04 | 1218.36 | 1400.69 | 1570.89 | 1801.06 | 2145.07 | 2587.14 | 3179.92 | 3989.63 |
| Guizhou | 228.60 | 277.69 | 338.17 | 396.50 | 456.26 | 575.89 | 663.89 | 768.76 | 871.46 | 996.24 | 1152.95 |
| Yunnan | 472.82 | 560.29 | 703.15 | 738.21 | 722.27 | 770.20 | 860.67 | 1068.51 | 1352.78 | 1755.30 | 2156.84 |
| Xizang | 23.41 | 26.94 | 30.46 | 36.98 | 38.26 | 41.99 | 68.63 | 100.97 | 168.45 | 196.19 | 232.35 |
| Shaanxi | 406.90 | 464.80 | 573.50 | 646.30 | 796.20 | 887.20 | 1016.90 | 1338.70 | 1471.30 | 1913.40 | 2430.20 |
| Gansu | 188.67 | 229.49 | 258.10 | 310.90 | 373.90 | 463.13 | 534.75 | 617.62 | 756.02 | 874.52 | 1027.78 |
| Qinghai | 79.24 | 101.55 | 116.38 | 131.12 | 156.97 | 202.17 | 244.00 | 282.18 | 314.93 | 364.08 | 411.68 |
| Ningxia | 77.30 | 88.10 | 108.70 | 130.60 | 160.80 | 195.80 | 230.80 | 318.20 | 379.70 | 444.80 | 515.30 |
| Xinjiang | 422.87 | 461.41 | 543.78 | 551.72 | 648.12 | 720.12 | 856.70 | 1079.24 | 1239.50 | 1485.17 | 1719.96 |
| **Province** | **2007** | **2008** | **2009** | **2010** | **2011** | **2012** | **2013** | **2014** | **2015** | **2016** | **2017** |
| Beijing | 4082.56 | 3754.49 | 4435.00 | 5342.40 | 5953.90 | 7032.80 | 7595.40 | 7957.20 | 8155.40 | 9716.10 | 10375.30 |
| Tianjin | 2681.35 | 3746.99 | 5077.90 | 6468.50 | 8069.90 | 9314.80 | 10438.80 | 11338.00 | 10495.80 | 10113.90 | 10137.50 |
| Hebei | 6211.94 | 8397.68 | 9390.20 | 10791.60 | 13688.80 | 15087.90 | 16167.10 | 17064.70 | 17298.80 | 18595.70 | 19035.40 |
| Shanxi | 2909.61 | 3587.61 | 4856.80 | 5973.10 | 7256.10 | 7663.40 | 8693.50 | 8818.50 | 8821.60 | 8369.40 | 6700.10 |
| Neimenggu | 4356.39 | 5596.45 | 7425.20 | 8938.70 | 10837.10 | 12954.30 | 15287.10 | 13453.90 | 13844.40 | 12489.50 | 10391.90 |
| Liaoning | 6031.30 | 9981.43 | 8906.40 | 11024.60 | 13474.60 | 15049.60 | 16479.80 | 16927.40 | 12098.90 | 9171.70 | 9639.00 |
| Jinlin | 4003.18 | 5608.30 | 6280.50 | 7618.10 | 8355.50 | 9235.00 | 9751.50 | 10372.70 | 11001.20 | 10185.70 | 10013.90 |
| Heilongjiang | 2877.84 | 3655.29 | 4995.00 | 5410.50 | 6599.30 | 7824.10 | 9153.50 | 9288.00 | 9664.10 | 9151.40 | 9651.40 |
| Shanghai | 5041.40 | 5419.43 | 6447.50 | 6380.30 | 6801.80 | 7012.50 | 7617.10 | 8022.50 | 8999.90 | 10631.40 | 11507.20 |
| Jiangsu | 11594.62 | 14418.47 | 17138.00 | 20709.10 | 24522.20 | 26415.50 | 27711.10 | 28796.20 | 29940.80 | 32254.60 | 36416.80 |
| Zhejiang | 8201.28 | 9369.14 | 10220.10 | 12101.30 | 13822.90 | 14607.60 | 16139.70 | 17000.90 | 18213.30 | 20677.80 | 21861.50 |
| Anhui | 3346.30 | 4290.19 | 4820.50 | 6061.10 | 7594.40 | 8680.90 | 9739.10 | 10723.80 | 11106.50 | 12345.10 | 13569.20 |
| Fujian | 4344.88 | 5601.36 | 6438.30 | 7341.60 | 9060.50 | 10270.20 | 11678.60 | 13038.00 | 14140.30 | 15576.10 | 17637.90 |
| Jiangxi | 2688.33 | 3203.51 | 4082.60 | 4740.30 | 5785.80 | 6301.10 | 6774.20 | 6876.90 | 7706.00 | 9029.50 | 9738.40 |
| Shandong | 11784.57 | 14141.48 | 17734.40 | 20800.60 | 24281.20 | 26808.90 | 29249.50 | 31647.10 | 33229.40 | 33548.70 | 34704.00 |
| Henan | 8043.35 | 10435.38 | 12996.10 | 15704.10 | 18819.20 | 21667.80 | 24376.00 | 26655.80 | 27722.50 | 29595.10 | 30415.30 |
| Hubei | 4371.14 | 5368.99 | 6612.90 | 8200.40 | 10597.80 | 12064.80 | 13701.90 | 15442.90 | 16757.40 | 18515.80 | 20586.50 |
| Hunan | 3945.45 | 5480.69 | 6666.80 | 8568.80 | 10487.30 | 11990.70 | 13573.90 | 15139.70 | 15085.00 | 16477.40 | 17159.50 |
| Guangdong | 9920.99 | 11405.38 | 14025.10 | 16812.70 | 19432.80 | 22033.80 | 24997.70 | 27930.80 | 29250.40 | 33279.70 | 38390.90 |
| Guangxi | 2831.62 | 3559.92 | 5533.20 | 7785.50 | 9745.70 | 10547.40 | 9725.60 | 10463.10 | 11264.70 | 12114.10 | 9034.50 |
| Hainan | 495.50 | 741.07 | 895.90 | 1179.40 | 1460.50 | 1947.90 | 2233.50 | 2549.30 | 2325.10 | 2519.10 | 2808.50 |
| Chongqing | 2587.86 | 3615.03 | 3633.20 | 4379.30 | 5511.20 | 6041.20 | 6581.00 | 7380.90 | 8042.50 | 9114.90 | 9907.30 |
| Sichuan | 5005.45 | 6579.56 | 7464.20 | 8911.10 | 10691.30 | 12096.20 | 13081.70 | 13990.60 | 14415.30 | 15800.50 | 17689.30 |
| Guizhou | 1361.79 | 1689.06 | 2046.80 | 2510.40 | 3147.50 | 4067.30 | 5141.80 | 5928.80 | 6913.90 | 7975.50 | 9085.80 |
| Yunnan | 2616.77 | 2616.77 | 3502.40 | 5213.10 | 6678.20 | 7949.50 | 9311.10 | 10918.80 | 12080.10 | 13321.10 | 14825.50 |
| Xizang | 271.67 | 312.86 | 379.40 | 561.40 | 547.50 | 709.10 | 898.40 | 1050.90 | 1027.00 | 1151.90 | 1372.90 |
| Shaanxi | 3152.28 | 4569.16 | 5270.30 | 6851.50 | 8190.20 | 9700.10 | 10779.50 | 11783.70 | 11662.80 | 12584.80 | 14144.10 |
| Gansu | 1221.96 | 1733.54 | 1788.30 | 2177.90 | 2685.80 | 3128.70 | 3649.10 | 4116.70 | 4412.30 | 4894.00 | 3557.10 |
| Qinghai | 480.43 | 583.20 | 791.80 | 1057.90 | 1369.90 | 1895.70 | 2418.30 | 2953.50 | 3303.60 | 3551.90 | 3918.30 |
| Ningxia | 621.82 | 858.64 | 1185.40 | 1490.00 | 1653.20 | 1967.20 | 2223.00 | 2987.70 | 3516.90 | 3757.10 | 3835.70 |
| Xinjiang | 2004.99 | 2227.63 | 2473.20 | 3233.00 | 3948.90 | 5477.60 | 6943.70 | 8301.40 | 8755.70 | 8655.10 | 10695.30 |
| **Province** | **2018** | **2019** | **2020** | **2021** | **2022** |  |  |  |  |  |  |
| Beijing | 7910.03 | 7712.28 | 7881.95 | 8268.16 | 8565.82 |  |  |  |  |  |  |
| Tianjin | 10656.72 | 12052.75 | 12414.33 | 13010.22 | 11722.21 |  |  |  |  |  |  |
| Hebei | 35411.21 | 37712.94 | 38919.75 | 40087.34 | 43254.24 |  |  |  |  |  |  |
| Shanxi | 6384.81 | 6978.60 | 7718.33 | 8389.82 | 8884.82 |  |  |  |  |  |  |
| Neimenggu | 10047.46 | 10720.64 | 10559.83 | 11594.70 | 13635.37 |  |  |  |  |  |  |
| Liaoning | 6923.74 | 6944.51 | 7125.07 | 7310.32 | 7573.49 |  |  |  |  |  |  |
| Jinlin | 13496.44 | 11310.02 | 12248.75 | 13596.11 | 13269.81 |  |  |  |  |  |  |
| Heilongjiang | 10761.28 | 11439.24 | 11851.05 | 12609.52 | 12685.17 |  |  |  |  |  |  |
| Shanghai | 7623.42 | 8012.22 | 8837.48 | 9544.47 | 9449.03 |  |  |  |  |  |  |
| Jiangsu | 56207.24 | 59073.80 | 59251.03 | 62687.58 | 65069.71 |  |  |  |  |  |  |
| Zhejiang | 33946.42 | 37341.06 | 39357.47 | 43608.08 | 47576.42 |  |  |  |  |  |  |
| Anhui | 32729.56 | 35740.68 | 37563.46 | 41094.42 | 44792.92 |  |  |  |  |  |  |
| Fujian | 29454.17 | 31191.97 | 31067.20 | 32931.24 | 35401.08 |  |  |  |  |  |  |
| Jiangxi | 24536.77 | 26794.15 | 28991.27 | 32122.33 | 34884.85 |  |  |  |  |  |  |
| Shandong | 57466.01 | 52753.80 | 54652.93 | 57932.11 | 61465.97 |  |  |  |  |  |  |
| Henan | 48101.15 | 51949.24 | 54183.06 | 56621.30 | 60414.92 |  |  |  |  |  |  |
| Hubei | 35833.46 | 39667.64 | 32210.13 | 38780.99 | 44598.14 |  |  |  |  |  |  |
| Hunan | 35155.12 | 38705.79 | 41647.43 | 44979.22 | 47947.85 |  |  |  |  |  |  |
| Guangdong | 41802.20 | 46442.25 | 49786.09 | 52922.61 | 51546.62 |  |  |  |  |  |  |
| Guangxi | 22713.00 | 24893.45 | 25938.98 | 27910.34 | 27938.25 |  |  |  |  |  |  |
| Hainan | 3713.85 | 3372.18 | 3641.95 | 4013.43 | 3844.86 |  |  |  |  |  |  |
| Chongqing | 18764.59 | 19815.41 | 20588.21 | 21844.09 | 21997.00 |  |  |  |  |  |  |
| Sichuan | 35156.11 | 38179.54 | 39248.57 | 41564.23 | 44058.09 |  |  |  |  |  |  |
| Guizhou | 17953.52 | 18115.10 | 18694.78 | 18115.24 | 17191.37 |  |  |  |  |  |  |
| Yunnan | 21132.58 | 22928.84 | 24694.37 | 25682.14 | 27608.30 |  |  |  |  |  |  |
| Xizang | 2169.21 | 2121.49 | 2236.05 | 1918.53 | 1573.19 |  |  |  |  |  |  |
| Shaanxi | 26296.62 | 26954.03 | 28059.15 | 27217.37 | 29421.98 |  |  |  |  |  |  |
| Gansu | 5600.52 | 5970.15 | 6435.82 | 7150.20 | 7872.37 |  |  |  |  |  |  |
| Qinghai | 4167.10 | 4375.46 | 3841.65 | 3730.24 | 3446.75 |  |  |  |  |  |  |
| Ningxia | 3049.83 | 2735.70 | 2845.13 | 2907.72 | 3204.31 |  |  |  |  |  |  |
| Xinjiang | 9042.65 | 9268.71 | 10770.24 | 12385.78 | 13327.10 |  |  |  |  |  |  |

**Appendix A2**

**The Price Index (1978=100)**

| **Province** | **1952** | **1953** | **1954** | **1955** | **1956** | **1957** | **1958** | **1959** | **1960** | **1961** | **1962** |
| --- | --- | --- | --- | --- | --- | --- | --- | --- | --- | --- | --- |
| Beijing | 131.34 | 131.16 | 131.16 | 131.37 | 132.14 | 130.43 | 132.17 | 130.87 | 131.44 | 131.11 | 136.75 |
| Tianjin | 90.34 | 98.02 | 99.78 | 99.88 | 99.68 | 101.48 | 100.06 | 100.06 | 101.36 | 113.82 | 113.94 |
| Hebei | 89.83 | 91.47 | 91.38 | 88.44 | 114.63 | 83.76 | 82.40 | 88.19 | 219.20 | 92.76 | 101.03 |
| Shanxi | 98.84 | 99.99 | 97.35 | 94.60 | 93.10 | 93.38 | 94.87 | 97.26 | 97.06 | 100.24 | 101.76 |
| Neimenggu | 98.17 | 101.43 | 98.19 | 102.27 | 98.53 | 100.80 | 99.42 | 99.78 | 100.01 | 98.77 | 98.87 |
| Liaoning | 34.96 | 35.07 | 33.98 | 30.72 | 29.70 | 31.31 | 28.17 | 27.56 | 28.27 | 32.22 | 33.19 |
| Jinlin | 92.78 | 90.22 | 92.10 | 88.21 | 79.57 | 92.58 | 94.14 | 93.70 | 96.94 | 77.51 | 98.72 |
| Heilongjiang | 103.88 | 111.16 | 114.01 | 107.84 | 99.55 | 97.63 | 104.99 | 105.08 | 106.91 | 135.33 | 117.60 |
| Shanghai | 145.73 | 136.91 | 131.38 | 125.47 | 116.98 | 173.09 | 121.17 | 115.37 | 115.77 | 116.14 | 120.22 |
| Jiangsu | 74.88 | 77.64 | 77.62 | 76.87 | 79.17 | 83.36 | 101.07 | 104.35 | 105.27 | 97.83 | 101.08 |
| Zhejiang | 62.63 | 82.41 | 77.46 | 115.89 | 95.26 | 60.68 | 43.24 | 42.03 | 51.55 | 81.84 | 98.69 |
| Anhui | 58.42 | 67.34 | 67.09 | 69.79 | 75.12 | 72.17 | 81.80 | 96.22 | 100.92 | 106.69 | 87.71 |
| Fujian | 99.49 | 98.45 | 97.26 | 93.55 | 90.74 | 89.18 | 89.42 | 96.95 | 96.67 | 95.04 | 101.72 |
| Jiangxi | 59.66 | 106.05 | 228.98 | 179.08 | 222.74 | 64.94 | 116.60 | 137.77 | 120.22 | 176.44 | 110.10 |
| Shandong | 113.56 | 116.60 | 118.11 | 119.90 | 116.17 | 117.15 | 92.78 | 93.02 | 99.64 | 113.35 | 119.73 |
| Henan | 106.13 | 114.94 | 107.12 | 116.61 | 108.67 | 108.16 | 109.86 | 112.37 | 111.28 | 130.60 | 117.11 |
| Hubei | 73.98 | 79.69 | 77.09 | 80.30 | 82.28 | 83.93 | 97.95 | 100.04 | 93.09 | 115.75 | 115.53 |
| Hunan | 123.17 | 120.27 | 118.92 | 112.70 | 107.35 | 109.47 | 104.05 | 107.41 | 110.89 | 123.36 | 128.52 |
| Guangdong | 90.86 | 91.77 | 91.49 | 91.77 | 91.40 | 93.50 | 94.06 | 95.19 | 96.24 | 107.88 | 108.42 |
| Guangxi | 93.74 | 65.70 | 66.14 | 56.31 | 72.61 | 68.41 | 66.17 | 71.73 | 66.32 | 72.86 | 73.13 |
| Hainan |  |  |  |  |  |  |  |  |  |  |  |
| Chongqing | 36.14 | 79.71 | 69.43 | 91.74 | 93.06 | 104.41 | 108.85 | 39.54 | 27.57 | 2943.30 | 399.85 |
| Sichuan | 36.14 | 79.71 | 69.43 | 91.74 | 93.06 | 104.41 | 108.85 | 39.54 | 27.57 | 2943.30 | 399.85 |
| Guizhou | 102.26 | 108.46 | 111.62 | 108.97 | 104.28 | 105.31 | 105.82 | 112.39 | 159.97 | 152.63 | 154.97 |
| Yunnan | 13.82 | 17.71 | 20.86 | 22.92 | 27.29 | 28.44 | 29.62 | 39.00 | 49.93 | 32.46 | 29.79 |
| Xizang | 71.73 | 71.73 | 71.75 | 71.74 | 71.74 | 71.75 | 71.76 | 71.77 | 71.77 | 71.77 | 71.78 |
| Shaanxi | 123.68 | 124.51 | 149.44 | 125.39 | 121.62 | 122.04 | 123.98 | 134.67 | 126.57 | 170.68 | 224.12 |
| Gansu | 136.62 | 109.92 | 103.17 | 95.74 | 57.53 | 78.38 | 52.19 | 61.86 | 66.50 | 289.57 | 449.51 |
| Qinghai | 71.73 | 71.73 | 71.75 | 71.74 | 71.74 | 71.75 | 71.76 | 71.77 | 71.77 | 71.77 | 71.78 |
| Ningxia | 81.21 | 80.83 | 80.81 | 81.94 | 83.93 | 89.30 | 90.54 | 92.04 | 94.67 | 111.26 | 107.44 |
| Xinjiang | 115.66 | 105.23 | 107.56 | 111.74 | 107.79 | 100.25 | 104.05 | 96.26 | 96.95 | 101.42 | 100.57 |
| **Province** | **1963** | **1964** | **1965** | **1966** | **1967** | **1968** | **1969** | **1970** | **1971** | **1972** | **1973** |
| Beijing | 99.92 | 99.87 | 100.44 | 100.29 | 99.87 | 100.13 | 99.83 | 100.21 | 99.51 | 101.44 | 99.23 |
| Tianjin | 110.52 | 105.10 | 101.32 | 100.61 | 100.01 | 99.91 | 100.51 | 99.60 | 99.50 | 99.50 | 99.90 |
| Hebei | 98.10 | 97.48 | 97.31 | 95.88 | 95.93 | 89.47 | 92.18 | 90.30 | 89.36 | 89.30 | 89.53 |
| Shanxi | 108.15 | 105.26 | 100.98 | 99.71 | 100.02 | 95.73 | 93.92 | 94.22 | 97.75 | 96.14 | 96.56 |
| Neimenggu | 99.74 | 100.08 | 99.63 | 99.63 | 100.57 | 100.57 | 99.96 | 100.26 | 100.32 | 99.45 | 100.12 |
| Liaoning | 32.06 | 59.87 | 52.14 | 53.30 | 55.50 | 56.76 | 53.91 | 52.17 | 54.41 | 91.79 | 92.48 |
| Jinlin | 98.88 | 103.48 | 96.71 | 97.15 | 99.39 | 100.19 | 101.47 | 101.97 | 100.15 | 100.48 | 100.36 |
| Heilongjiang | 115.36 | 122.49 | 118.90 | 119.22 | 118.47 | 117.92 | 109.18 | 111.84 | 103.77 | 103.91 | 103.47 |
| Shanghai | 118.51 | 116.46 | 113.10 | 111.21 | 109.71 | 108.17 | 106.64 | 103.20 | 101.94 | 99.57 | 98.86 |
| Jiangsu | 97.32 | 98.23 | 98.72 | 96.27 | 94.13 | 94.51 | 95.29 | 95.19 | 96.15 | 96.89 | 97.27 |
| Zhejiang | 86.83 | 60.71 | 59.52 | 63.91 | 68.83 | 62.71 | 47.96 | 49.58 | 53.89 | 60.26 | 71.04 |
| Anhui | 91.13 | 86.76 | 85.23 | 91.93 | 87.64 | 86.02 | 90.72 | 91.95 | 93.34 | 95.01 | 99.27 |
| Fujian | 111.55 | 104.78 | 100.97 | 99.26 | 99.85 | 99.75 | 93.74 | 93.82 | 94.76 | 96.03 | 96.17 |
| Jiangxi | 183.97 | 157.61 | 63.52 | 118.10 | 96.75 | 101.12 | 169.37 | 121.24 | 101.84 | 115.76 | 123.54 |
| Shandong | 114.36 | 100.34 | 116.61 | 103.73 | 102.77 | 103.72 | 104.63 | 106.00 | 96.62 | 93.87 | 93.95 |
| Henan | 105.41 | 103.41 | 97.95 | 95.76 | 96.75 | 97.78 | 96.16 | 95.42 | 96.87 | 97.96 | 99.68 |
| Hubei | 111.41 | 119.47 | 98.69 | 95.85 | 96.32 | 98.12 | 94.36 | 82.61 | 89.68 | 90.56 | 96.03 |
| Hunan | 129.03 | 122.93 | 112.95 | 109.88 | 107.85 | 112.23 | 106.69 | 103.27 | 98.88 | 96.67 | 98.97 |
| Guangdong | 106.36 | 102.96 | 101.52 | 101.11 | 101.42 | 101.21 | 100.91 | 100.20 | 99.80 | 99.50 | 99.50 |
| Guangxi | 76.29 | 79.27 | 85.38 | 83.11 | 87.83 | 87.24 | 81.73 | 78.38 | 74.15 | 78.53 | 88.52 |
| Hainan |  |  |  |  |  |  |  |  |  |  |  |
| Chongqing | 90.88 | 82.28 | 60.11 | 39.88 | 61.31 | 95.67 | 38.92 | 34.92 | 55.84 | 55.93 | 55.78 |
| Sichuan | 90.88 | 82.28 | 60.11 | 39.88 | 61.31 | 95.67 | 38.92 | 34.92 | 55.84 | 55.93 | 55.78 |
| Guizhou | 135.06 | 137.48 | 129.36 | 133.18 | 137.38 | 131.59 | 131.38 | 133.19 | 112.02 | 100.72 | 99.27 |
| Yunnan | 31.36 | 36.48 | 46.18 | 53.68 | 48.35 | 31.00 | 50.81 | 62.00 | 65.26 | 72.51 | 79.73 |
| Xizang | 71.76 | 71.76 | 71.77 | 71.77 | 71.76 | 71.77 | 71.77 | 71.77 | 71.76 | 71.77 | 71.77 |
| Shaanxi | 168.69 | 152.00 | 116.17 | 124.19 | 114.48 | 157.06 | 118.20 | 93.62 | 95.25 | 94.80 | 91.59 |
| Gansu | 235.50 | 146.41 | 80.13 | 87.10 | 88.77 | 101.02 | 96.41 | 90.25 | 93.86 | 109.13 | 115.55 |
| Qinghai | 71.76 | 71.76 | 71.77 | 71.77 | 71.76 | 71.77 | 71.77 | 71.77 | 71.76 | 71.77 | 71.77 |
| Ningxia | 96.15 | 91.98 | 90.16 | 87.90 | 89.87 | 90.10 | 91.80 | 92.17 | 91.33 | 91.51 | 91.66 |
| Xinjiang | 96.76 | 100.47 | 100.13 | 99.25 | 96.57 | 94.27 | 93.60 | 93.27 | 102.80 | 108.04 | 97.37 |
| **Province** | **1974** | **1975** | **1976** | **1977** | **1978** | **1979** | **1980** | **1981** | **1982** | **1983** | **1984** |
| Beijing | 99.71 | 99.74 | 370.23 | 99.24 | 100.00 | 97.54 | 102.19 | 136.11 | 133.93 | 114.36 | 105.71 |
| Tianjin | 99.60 | 99.70 | 99.70 | 100.00 | 100.00 | 101.10 | 106.66 | 108.26 | 108.80 | 109.35 | 111.31 |
| Hebei | 89.24 | 89.44 | 89.12 | 89.68 | 100.00 | 99.29 | 104.40 | 106.74 | 108.56 | 108.33 | 126.07 |
| Shanxi | 96.41 | 97.77 | 98.40 | 99.52 | 100.00 | 102.52 | 105.60 | 108.69 | 111.34 | 114.19 | 121.85 |
| Neimenggu | 99.41 | 99.84 | 99.78 | 100.01 | 100.00 | 100.02 | 99.87 | 99.57 | 99.57 | 99.90 | 108.63 |
| Liaoning | 93.66 | 110.54 | 103.24 | 98.34 | 100.00 | 105.47 | 105.13 | 108.10 | 116.85 | 118.52 | 120.05 |
| Jinlin | 100.29 | 99.63 | 99.31 | 99.31 | 100.00 | 100.45 | 100.66 | 107.80 | 110.19 | 111.30 | 122.30 |
| Heilongjiang | 103.94 | 103.93 | 103.91 | 103.81 | 100.00 | 101.30 | 102.04 | 103.47 | 104.73 | 130.23 | 120.42 |
| Shanghai | 96.94 | 97.08 | 97.66 | 100.49 | 100.00 | 103.04 | 107.78 | 105.54 | 105.58 | 105.05 | 108.94 |
| Jiangsu | 96.98 | 97.84 | 96.24 | 97.23 | 100.00 | 95.32 | 105.51 | 98.91 | 96.49 | 94.04 | 99.08 |
| Zhejiang | 76.82 | 89.54 | 81.62 | 83.27 | 100.00 | 157.15 | 147.60 | 166.36 | 147.72 | 165.14 | 162.58 |
| Anhui | 97.23 | 136.50 | 96.73 | 97.79 | 100.00 | 106.08 | 113.29 | 112.20 | 112.25 | 124.60 | 128.01 |
| Fujian | 96.13 | 97.52 | 97.86 | 99.56 | 100.00 | 102.25 | 105.36 | 108.90 | 111.39 | 114.12 | 119.24 |
| Jiangxi | 114.81 | 140.10 | 105.99 | 118.02 | 100.00 | 104.44 | 97.10 | 110.86 | 95.22 | 93.78 | 98.55 |
| Shandong | 94.97 | 92.74 | 97.61 | 97.60 | 100.00 | 103.26 | 107.22 | 116.84 | 118.36 | 126.76 | 126.72 |
| Henan | 102.87 | 103.11 | 88.15 | 105.86 | 100.00 | 109.55 | 110.38 | 110.80 | 111.22 | 113.12 | 124.99 |
| Hubei | 97.01 | 103.20 | 79.52 | 95.26 | 100.00 | 102.70 | 116.18 | 120.19 | 118.37 | 118.33 | 126.00 |
| Hunan | 105.71 | 100.69 | 103.91 | 100.68 | 100.00 | 101.41 | 105.23 | 106.63 | 106.19 | 107.47 | 112.30 |
| Guangdong | 99.40 | 99.40 | 99.50 | 99.60 | 100.00 | 106.50 | 120.41 | 126.33 | 123.14 | 126.01 | 142.77 |
| Guangxi | 89.85 | 98.96 | 104.47 | 101.18 | 100.00 | 106.66 | 114.23 | 128.74 | 135.48 | 126.94 | 138.84 |
| Hainan |  |  |  |  | 100.00 | 106.50 | 120.41 | 126.33 | 123.14 | 126.01 | 142.77 |
| Chongqing | 60.88 | 47.55 | 81.51 | 112.56 | 100.00 | 119.00 | 137.16 | 125.51 | 125.51 | 125.26 | 131.77 |
| Sichuan | 60.88 | 47.55 | 81.51 | 112.56 | 100.00 | 119.00 | 137.16 | 125.51 | 125.51 | 125.26 | 131.77 |
| Guizhou | 100.13 | 101.14 | 101.20 | 99.54 | 100.00 | 101.85 | 106.81 | 106.19 | 107.85 | 98.20 | 110.91 |
| Yunnan | 83.44 | 79.44 | 69.69 | 81.99 | 100.00 | 104.17 | 110.84 | 117.83 | 132.47 | 145.65 | 169.36 |
| Xizang | 136.27 | 100.00 | 100.00 | 100.00 | 100.00 | 99.99 | 100.01 | 102.66 | 102.73 | 125.91 | 114.36 |
| Shaanxi | 93.15 | 95.16 | 94.01 | 97.15 | 100.00 | 99.88 | 98.46 | 99.41 | 117.78 | 145.21 | 110.40 |
| Gansu | 123.85 | 123.73 | 122.48 | 124.28 | 100.00 | 102.71 | 120.65 | 135.04 | 136.09 | 141.15 | 118.20 |
| Qinghai | 136.27 | 100.00 | 100.00 | 100.00 | 100.00 | 99.99 | 100.01 | 102.66 | 102.73 | 125.91 | 114.36 |
| Ningxia | 91.85 | 92.10 | 92.40 | 99.40 | 100.00 | 100.92 | 106.80 | 108.58 | 111.79 | 112.83 | 116.70 |
| Xinjiang | 92.62 | 107.70 | 94.01 | 94.42 | 100.00 | 94.45 | 114.98 | 115.17 | 118.65 | 121.86 | 128.04 |
| **Province** | **1985** | **1986** | **1987** | **1988** | **1989** | **1990** | **1991** | **1992** | **1993** | **1994** | **1995** |
| Beijing | 103.99 | 89.34 | 87.26 | 89.80 | 75.88 | 85.58 | 91.83 | 103.03 | 130.44 | 151.57 | 172.64 |
| Tianjin | 126.79 | 135.92 | 145.29 | 171.01 | 189.12 | 196.43 | 220.98 | 263.85 | 324.28 | 362.87 | 390.44 |
| Hebei | 137.86 | 139.59 | 135.62 | 164.94 | 158.76 | 172.82 | 184.58 | 238.66 | 297.84 | 327.63 | 350.23 |
| Shanxi | 122.20 | 128.57 | 140.74 | 164.53 | 184.57 | 197.15 | 212.53 | 248.23 | 309.79 | 335.50 | 358.32 |
| Neimenggu | 113.70 | 120.78 | 125.44 | 134.47 | 153.35 | 147.20 | 144.56 | 179.98 | 196.72 | 209.90 | 218.08 |
| Liaoning | 130.56 | 141.68 | 157.41 | 176.67 | 175.05 | 185.38 | 200.58 | 242.50 | 330.77 | 388.33 | 407.35 |
| Jinlin | 131.31 | 138.61 | 142.36 | 156.60 | 184.09 | 187.24 | 189.13 | 213.41 | 245.62 | 298.45 | 319.41 |
| Heilongjiang | 127.88 | 141.84 | 154.34 | 175.25 | 203.23 | 214.86 | 230.97 | 262.16 | 335.56 | 365.76 | 389.53 |
| Shanghai | 119.50 | 129.25 | 139.46 | 157.68 | 174.63 | 182.15 | 194.60 | 214.71 | 282.13 | 306.95 | 316.47 |
| Jiangsu | 110.31 | 106.14 | 117.15 | 129.45 | 123.46 | 125.10 | 130.73 | 146.54 | 203.40 | 233.10 | 250.35 |
| Zhejiang | 129.48 | 128.56 | 141.82 | 174.32 | 219.27 | 267.81 | 272.80 | 245.05 | 333.27 | 374.93 | 401.92 |
| Anhui | 132.47 | 149.87 | 158.00 | 183.76 | 184.64 | 187.20 | 214.90 | 257.45 | 316.67 | 380.32 | 405.04 |
| Fujian | 138.36 | 155.93 | 171.26 | 213.32 | 247.71 | 300.82 | 326.70 | 375.37 | 503.37 | 540.12 | 566.05 |
| Jiangxi | 99.76 | 89.87 | 112.07 | 152.62 | 116.02 | 118.82 | 131.17 | 144.42 | 187.46 | 214.83 | 230.30 |
| Shandong | 133.22 | 143.63 | 152.41 | 171.30 | 184.06 | 215.88 | 242.65 | 289.73 | 353.76 | 409.30 | 436.31 |
| Henan | 140.83 | 145.70 | 166.62 | 156.14 | 162.30 | 174.43 | 190.82 | 228.60 | 289.64 | 307.02 | 325.14 |
| Hubei | 136.36 | 149.85 | 159.28 | 179.81 | 159.08 | 169.10 | 183.13 | 214.27 | 272.98 | 294.54 | 309.27 |
| Hunan | 118.14 | 128.54 | 140.24 | 167.16 | 176.69 | 189.59 | 204.94 | 238.55 | 308.93 | 350.63 | 383.94 |
| Guangdong | 154.50 | 155.17 | 158.18 | 192.16 | 169.35 | 184.76 | 188.42 | 231.23 | 266.43 | 273.74 | 306.12 |
| Guangxi | 148.14 | 158.44 | 171.22 | 264.24 | 254.79 | 264.47 | 274.52 | 323.66 | 424.64 | 476.87 | 493.08 |
| Hainan | 154.50 | 155.17 | 158.18 | 192.16 | 169.35 | 184.76 | 211.93 | 314.82 | 413.04 | 463.85 | 479.62 |
| Chongqing | 141.65 | 152.70 | 160.79 | 176.71 | 198.45 | 208.97 | 225.89 | 257.29 | 342.71 | 373.21 | 388.89 |
| Sichuan | 141.65 | 152.70 | 160.79 | 176.71 | 198.45 | 208.97 | 225.89 | 257.29 | 342.71 | 368.07 | 372.49 |
| Guizhou | 114.72 | 148.03 | 156.88 | 167.14 | 191.13 | 202.50 | 219.58 | 253.37 | 319.18 | 330.55 | 355.67 |
| Yunnan | 192.46 | 202.41 | 201.76 | 226.80 | 243.00 | 259.53 | 290.93 | 342.14 | 463.25 | 499.39 | 519.36 |
| Xizang | 128.42 | 132.62 | 133.69 | 143.56 | 134.15 | 140.18 | 152.38 | 165.03 | 119.37 | 128.69 | 127.10 |
| Shaanxi | 105.46 | 88.87 | 121.00 | 129.74 | 147.50 | 174.30 | 194.31 | 205.13 | 265.65 | 296.73 | 320.17 |
| Gansu | 111.01 | 110.99 | 116.07 | 126.02 | 135.69 | 143.29 | 156.18 | 183.36 | 231.40 | 260.55 | 285.05 |
| Qinghai | 128.42 | 132.62 | 133.69 | 143.56 | 134.15 | 134.20 | 160.37 | 184.58 | 232.39 | 251.91 | 265.26 |
| Ningxia | 125.59 | 131.68 | 142.00 | 166.82 | 196.65 | 204.81 | 226.11 | 265.23 | 326.23 | 367.33 | 401.49 |
| Xinjiang | 143.81 | 154.14 | 175.69 | 196.50 | 224.60 | 185.65 | 213.13 | 249.36 | 315.44 | 354.24 | 376.20 |
| **Province** | **1996** | **1997** | **1998** | **1999** | **2000** | **2001** | **2002** | **2003** | **2004** | **2005** | **2006** |
| Beijing | 186.79 | 191.84 | 193.37 | 193.18 | 195.11 | 196.28 | 197.06 | 201.40 | 210.06 | 211.53 | 212.38 |
| Tianjin | 400.21 | 403.41 | 398.97 | 395.78 | 395.38 | 394.20 | 392.22 | 402.42 | 431.80 | 436.98 | 440.04 |
| Hebei | 363.89 | 369.35 | 361.23 | 359.06 | 363.01 | 362.65 | 360.83 | 369.13 | 394.97 | 402.48 | 409.32 |
| Shanxi | 376.23 | 381.88 | 377.30 | 376.16 | 382.93 | 389.44 | 391.39 | 402.74 | 423.68 | 436.40 | 442.94 |
| Neimenggu | 229.64 | 228.95 | 232.85 | 237.27 | 241.78 | 243.71 | 246.15 | 252.55 | 265.18 | 274.99 | 284.06 |
| Liaoning | 416.32 | 425.89 | 425.04 | 425.04 | 429.72 | 431.43 | 434.45 | 445.75 | 467.15 | 480.23 | 490.31 |
| Jinlin | 337.48 | 352.33 | 355.15 | 362.97 | 370.23 | 374.30 | 378.79 | 382.96 | 398.66 | 406.63 | 415.58 |
| Heilongjiang | 402.78 | 413.65 | 416.96 | 415.71 | 421.95 | 422.37 | 423.21 | 432.95 | 452.86 | 462.83 | 472.55 |
| Shanghai | 338.62 | 340.31 | 334.87 | 328.51 | 328.51 | 330.81 | 331.80 | 339.76 | 362.53 | 365.43 | 365.79 |
| Jiangsu | 258.36 | 256.29 | 253.22 | 248.91 | 251.90 | 253.91 | 257.22 | 268.28 | 293.23 | 295.86 | 299.41 |
| Zhejiang | 407.15 | 405.11 | 395.39 | 388.27 | 389.44 | 391.00 | 392.56 | 406.30 | 430.27 | 431.56 | 438.04 |
| Anhui | 418.81 | 424.25 | 424.25 | 421.28 | 428.02 | 425.88 | 430.57 | 445.64 | 472.82 | 477.55 | 486.62 |
| Fujian | 592.65 | 599.17 | 587.19 | 578.38 | 579.54 | 576.64 | 574.91 | 582.96 | 602.78 | 607.00 | 619.14 |
| Jiangxi | 243.65 | 247.07 | 252.25 | 248.72 | 252.20 | 249.43 | 249.43 | 262.15 | 281.55 | 282.96 | 292.01 |
| Shandong | 449.84 | 451.64 | 448.02 | 446.23 | 456.94 | 463.34 | 468.44 | 482.02 | 517.69 | 532.70 | 542.29 |
| Henan | 337.82 | 347.61 | 343.09 | 336.23 | 345.98 | 347.37 | 342.85 | 355.88 | 391.82 | 397.31 | 403.66 |
| Hubei | 321.64 | 328.39 | 330.04 | 328.39 | 333.97 | 334.30 | 333.63 | 344.64 | 365.32 | 373.36 | 380.08 |
| Hunan | 402.76 | 410.01 | 421.08 | 423.18 | 432.91 | 438.54 | 440.30 | 452.63 | 477.52 | 494.71 | 510.05 |
| Guangdong | 315.42 | 332.69 | 334.04 | 329.03 | 336.15 | 336.82 | 335.81 | 343.20 | 365.16 | 371.00 | 373.60 |
| Guangxi | 510.84 | 512.37 | 511.86 | 491.89 | 498.78 | 508.76 | 510.28 | 519.47 | 543.36 | 550.97 | 557.58 |
| Hainan | 496.89 | 498.38 | 497.88 | 478.46 | 487.07 | 488.53 | 479.74 | 495.09 | 522.82 | 529.09 | 534.38 |
| Chongqing | 420.39 | 427.53 | 421.98 | 424.09 | 434.69 | 438.17 | 441.23 | 454.03 | 477.18 | 488.16 | 496.46 |
| Sichuan | 390.37 | 398.96 | 388.98 | 390.93 | 394.45 | 400.36 | 402.36 | 411.22 | 439.18 | 456.31 | 469.54 |
| Guizhou | 374.88 | 380.13 | 380.13 | 377.85 | 386.16 | 387.71 | 388.48 | 396.25 | 415.67 | 421.49 | 426.12 |
| Yunnan | 541.69 | 570.95 | 581.22 | 585.29 | 594.66 | 600.60 | 600.60 | 613.82 | 662.92 | 693.42 | 705.90 |
| Xizang | 111.61 | 115.19 | 112.76 | 125.36 | 145.08 | 251.94 | 254.03 | 259.18 | 363.97 | 366.88 | 367.61 |
| Shaanxi | 345.14 | 363.43 | 369.98 | 374.42 | 387.90 | 397.98 | 405.94 | 412.44 | 430.58 | 446.51 | 458.12 |
| Gansu | 299.01 | 307.09 | 308.01 | 311.09 | 318.87 | 325.24 | 325.89 | 331.43 | 349.66 | 357.36 | 372.01 |
| Qinghai | 274.55 | 282.78 | 278.54 | 278.82 | 283.28 | 284.13 | 293.22 | 299.09 | 307.46 | 313.92 | 321.45 |
| Ningxia | 431.21 | 440.69 | 449.95 | 448.60 | 468.78 | 475.82 | 479.15 | 490.17 | 514.18 | 524.98 | 531.81 |
| Xinjiang | 397.27 | 409.98 | 418.18 | 414.00 | 428.90 | 439.63 | 440.51 | 455.48 | 475.98 | 489.31 | 500.07 |
| **Province** | **2007** | **2008** | **2009** | **2010** | **2011** | **2012** | **2013** | **2014** | **2015** | **2016** | **2017** |
| Beijing | 218.32 | 235.31 | 228.48 | 234.20 | 247.55 | 250.76 | 250.51 | 250.51 | 244.50 | 243.77 | 255.22 |
| Tianjin | 451.48 | 493.02 | 481.18 | 493.70 | 521.84 | 521.84 | 519.23 | 521.82 | 521.30 | 518.17 | 540.45 |
| Hebei | 424.87 | 465.74 | 449.44 | 466.07 | 491.71 | 493.18 | 492.69 | 493.67 | 483.80 | 480.90 | 513.12 |
| Shanxi | 461.10 | 522.29 | 512.37 | 531.32 | 560.55 | 567.27 | 570.11 | 567.83 | 557.61 | 557.61 | 592.74 |
| Neimenggu | 294.86 | 318.80 | 314.02 | 330.98 | 351.83 | 357.46 | 356.03 | 355.31 | 348.21 | 346.47 | 358.25 |
| Liaoning | 511.39 | 557.98 | 541.24 | 559.10 | 596.01 | 601.97 | 601.97 | 600.16 | 587.56 | 582.86 | 606.17 |
| Jinlin | 431.78 | 463.17 | 460.40 | 471.45 | 497.85 | 499.84 | 499.84 | 500.84 | 488.82 | 482.46 | 505.14 |
| Heilongjiang | 493.81 | 538.11 | 525.19 | 552.50 | 593.94 | 598.69 | 599.29 | 599.29 | 593.30 | 589.74 | 609.79 |
| Shanghai | 378.59 | 408.54 | 396.28 | 411.34 | 438.08 | 435.45 | 436.32 | 438.50 | 425.35 | 423.65 | 452.03 |
| Jiangsu | 314.09 | 345.62 | 337.67 | 354.89 | 379.02 | 373.72 | 375.59 | 379.72 | 365.29 | 360.91 | 388.33 |
| Zhejiang | 457.31 | 499.98 | 483.48 | 506.20 | 544.16 | 539.81 | 539.81 | 543.05 | 528.93 | 526.29 | 556.81 |
| Anhui | 512.90 | 561.27 | 538.82 | 567.91 | 613.91 | 620.05 | 621.29 | 623.16 | 603.84 | 599.01 | 643.34 |
| Fujian | 655.67 | 694.42 | 680.53 | 702.98 | 746.57 | 748.81 | 749.56 | 752.56 | 739.76 | 739.76 | 781.19 |
| Jiangxi | 307.78 | 339.82 | 326.57 | 342.24 | 370.99 | 374.70 | 376.20 | 376.58 | 364.53 | 364.53 | 386.76 |
| Shandong | 563.98 | 607.30 | 588.47 | 609.66 | 651.11 | 656.32 | 658.95 | 660.92 | 645.72 | 639.91 | 677.02 |
| Henan | 422.23 | 460.07 | 443.50 | 459.03 | 492.99 | 497.92 | 497.43 | 497.43 | 485.49 | 481.60 | 517.24 |
| Hubei | 395.66 | 432.74 | 427.54 | 447.64 | 480.32 | 488.96 | 491.41 | 496.32 | 493.34 | 493.84 | 522.97 |
| Hunan | 539.63 | 592.78 | 591.00 | 614.64 | 658.90 | 670.10 | 678.81 | 688.99 | 691.75 | 694.52 | 734.10 |
| Guangdong | 382.57 | 415.51 | 401.80 | 413.85 | 436.61 | 443.16 | 449.36 | 456.10 | 451.54 | 452.90 | 476.90 |
| Guangxi | 570.40 | 615.18 | 602.26 | 620.33 | 658.79 | 662.74 | 663.41 | 674.02 | 665.93 | 662.60 | 691.76 |
| Hainan | 566.98 | 642.27 | 627.50 | 660.13 | 702.38 | 716.43 | 711.41 | 715.68 | 711.39 | 712.10 | 741.30 |
| Chongqing | 523.76 | 577.24 | 564.54 | 576.40 | 610.40 | 621.39 | 624.50 | 626.37 | 615.10 | 608.33 | 640.57 |
| Sichuan | 491.61 | 553.16 | 543.75 | 557.35 | 586.33 | 592.19 | 594.56 | 597.53 | 584.99 | 583.82 | 628.77 |
| Guizhou | 441.04 | 480.07 | 482.47 | 495.50 | 522.25 | 530.09 | 534.86 | 540.74 | 532.09 | 524.64 | 556.64 |
| Yunnan | 735.54 | 789.61 | 774.60 | 795.52 | 832.11 | 843.76 | 853.04 | 861.57 | 853.82 | 854.67 | 896.55 |
| Xizang | 373.86 | 388.44 | 386.50 | 390.36 | 404.81 | 416.55 | 429.04 | 438.48 | 444.62 | 453.96 | 460.31 |
| Shaanxi | 476.45 | 521.85 | 518.20 | 536.86 | 568.53 | 583.31 | 594.98 | 601.52 | 594.30 | 593.71 | 625.18 |
| Gansu | 382.42 | 407.93 | 414.05 | 428.54 | 448.68 | 458.10 | 459.94 | 460.40 | 449.81 | 443.96 | 470.15 |
| Qinghai | 334.95 | 370.09 | 373.42 | 387.61 | 412.81 | 421.89 | 428.22 | 432.07 | 424.29 | 422.60 | 448.37 |
| Ningxia | 548.82 | 598.05 | 599.25 | 624.42 | 671.25 | 681.32 | 679.96 | 685.40 | 668.26 | 665.59 | 704.86 |
| Xinjiang | 522.08 | 580.50 | 568.89 | 595.05 | 637.30 | 641.13 | 644.33 | 646.27 | 635.28 | 634.64 | 656.86 |
| **Province** | **2018** | **2019** | **2020** | **2021** | **2022** |  |  |  |  |  |  |
| Beijing | 264.92 | 270.49 | 273.19 | 277.83 | 282.84 |  |  |  |  |  |  |
| Tianjin | 564.78 | 574.38 | 580.12 | 588.82 | 600.60 |  |  |  |  |  |  |
| Hebei | 538.77 | 554.94 | 562.71 | 573.40 | 587.73 |  |  |  |  |  |  |
| Shanxi | 619.41 | 644.19 | 649.99 | 667.53 | 692.23 |  |  |  |  |  |  |
| Neimenggu | 371.14 | 377.45 | 379.34 | 393.76 | 408.72 |  |  |  |  |  |  |
| Liaoning | 627.39 | 646.83 | 653.95 | 666.37 | 683.70 |  |  |  |  |  |  |
| Jinlin | 528.37 | 542.11 | 545.91 | 555.73 | 572.96 |  |  |  |  |  |  |
| Heilongjiang | 629.91 | 634.95 | 644.47 | 654.78 | 671.15 |  |  |  |  |  |  |
| Shanghai | 477.35 | 484.03 | 488.38 | 494.73 | 503.14 |  |  |  |  |  |  |
| Jiangsu | 411.63 | 416.99 | 424.49 | 434.25 | 446.85 |  |  |  |  |  |  |
| Zhejiang | 588.55 | 600.91 | 608.12 | 621.50 | 641.39 |  |  |  |  |  |  |
| Anhui | 680.65 | 696.30 | 707.44 | 718.76 | 738.17 |  |  |  |  |  |  |
| Fujian | 819.47 | 831.76 | 842.57 | 851.84 | 874.84 |  |  |  |  |  |  |
| Jiangxi | 411.52 | 421.39 | 428.14 | 433.27 | 444.54 |  |  |  |  |  |  |
| Shandong | 718.32 | 738.44 | 753.21 | 763.75 | 781.32 |  |  |  |  |  |  |
| Henan | 545.17 | 562.62 | 567.68 | 576.20 | 591.75 |  |  |  |  |  |  |
| Hubei | 557.49 | 579.79 | 592.54 | 599.65 | 616.44 |  |  |  |  |  |  |
| Hunan | 769.34 | 782.42 | 792.59 | 805.27 | 831.04 |  |  |  |  |  |  |
| Guangdong | 506.47 | 527.74 | 531.96 | 539.41 | 552.90 |  |  |  |  |  |  |
| Guangxi | 722.89 | 740.24 | 750.60 | 758.86 | 775.55 |  |  |  |  |  |  |
| Hainan | 787.26 | 813.23 | 826.25 | 836.99 | 854.56 |  |  |  |  |  |  |
| Chongqing | 672.60 | 702.87 | 718.33 | 728.39 | 746.60 |  |  |  |  |  |  |
| Sichuan | 669.01 | 679.72 | 698.07 | 707.84 | 728.37 |  |  |  |  |  |  |
| Guizhou | 585.59 | 599.06 | 608.64 | 615.94 | 634.42 |  |  |  |  |  |  |
| Yunnan | 940.48 | 962.12 | 985.21 | 999.00 | 1029.97 |  |  |  |  |  |  |
| Xizang | 467.22 | 476.56 | 486.09 | 493.39 | 506.71 |  |  |  |  |  |  |
| Shaanxi | 658.94 | 676.07 | 688.91 | 699.94 | 721.63 |  |  |  |  |  |  |
| Gansu | 491.78 | 504.57 | 511.13 | 521.35 | 540.64 |  |  |  |  |  |  |
| Qinghai | 467.65 | 479.35 | 490.85 | 498.21 | 514.15 |  |  |  |  |  |  |
| Ningxia | 729.53 | 744.12 | 748.58 | 763.56 | 781.88 |  |  |  |  |  |  |
| Xinjiang | 681.16 | 700.23 | 704.43 | 718.52 | 738.64 |  |  |  |  |  |  |

**Appendix A3**

**The Variable Depreciation Rate (%)**

| **Province** | **1952** | **1953** | **1954** | **1955** | **1956** | **1957** | **1958** | **1959** | **1960** | **1961** | **1962** |
| --- | --- | --- | --- | --- | --- | --- | --- | --- | --- | --- | --- |
| Beijing | 9.71 | 23.66 | 10.70 | 11.68 | 12.02 | 13.05 | 11.54 | 15.18 | 13.45 | 5.45 | 7.29 |
| Tianjin | 10.81 | 12.67 | 9.14 | 10.45 | 12.81 | 11.66 | 14.26 | 13.44 | 10.96 | 6.01 | 8.13 |
| Hebei | 12.18 | 9.10 | 10.43 | 11.26 | 10.52 | 10.86 | 13.30 | 12.50 | 10.35 | 5.88 | 8.60 |
| Shanxi | 11.30 | 9.88 | 10.27 | 10.93 | 10.22 | 11.16 | 11.78 | 10.18 | 10.17 | 5.45 | 9.82 |
| Neimenggu | 11.26 | 10.87 | 11.83 | 9.07 | 14.57 | 11.36 | 13.71 | 13.26 | 10.04 | 6.16 | 9.17 |
| Liaoning | 9.48 | 12.40 | 10.72 | 10.92 | 13.41 | 11.08 | 15.96 | 13.32 | 11.91 | 3.49 | 8.60 |
| Jinlin | 10.41 | 10.04 | 10.38 | 10.60 | 11.90 | 10.22 | 14.07 | 12.70 | 11.69 | 6.57 | 9.44 |
| Heilongjiang | 11.00 | 10.23 | 10.83 | 10.95 | 10.82 | 11.08 | 15.57 | 12.79 | 11.56 | 5.32 | 9.54 |
| Shanghai | 11.38 | 13.21 | 10.02 | 10.16 | 12.82 | 11.01 | 14.53 | 14.90 | 14.11 | 5.89 | 6.78 |
| Jiangsu | 9.48 | 9.87 | 9.68 | 11.46 | 10.31 | 10.14 | 10.46 | 10.50 | 11.00 | 9.00 | 9.00 |
| Zhejiang | 11.13 | 10.18 | 10.07 | 11.17 | 10.95 | 11.21 | 12.77 | 11.38 | 10.26 | 7.68 | 10.02 |
| Anhui | 9.61 | 9.27 | 10.01 | 13.38 | 8.48 | 12.72 | 11.29 | 9.87 | 9.86 | 6.88 | 10.55 |
| Fujian | 12.03 | 10.47 | 10.16 | 11.31 | 12.95 | 10.87 | 11.74 | 12.70 | 10.76 | 6.92 | 9.61 |
| Jiangxi | 12.06 | 9.28 | 9.87 | 10.87 | 9.84 | 13.14 | 11.79 | 11.21 | 11.06 | 8.77 | 8.78 |
| Shandong | 9.72 | 9.37 | 11.23 | 11.27 | 11.44 | 9.68 | 12.90 | 10.96 | 9.09 | 7.68 | 9.47 |
| Henan | 10.14 | 9.88 | 10.27 | 10.93 | 10.22 | 11.16 | 11.78 | 10.18 | 10.17 | 5.45 | 9.82 |
| Hubei | 11.01 | 10.62 | 7.87 | 13.34 | 13.53 | 10.95 | 13.45 | 10.00 | 10.56 | 6.71 | 9.87 |
| Hunan | 10.40 | 10.03 | 9.46 | 12.33 | 10.69 | 11.85 | 13.03 | 11.55 | 10.44 | 6.06 | 10.10 |
| Guangdong | 11.26 | 10.72 | 10.75 | 10.37 | 10.98 | 11.20 | 11.84 | 10.78 | 10.23 | 7.66 | 10.93 |
| Guangxi | 11.10 | 10.07 | 11.20 | 11.24 | 11.21 | 11.54 | 12.44 | 11.54 | 10.39 | 8.58 | 9.81 |
| Hainan | 11.26 | 10.72 | 10.75 | 10.37 | 10.98 | 11.20 | 11.84 | 10.78 | 10.23 | 7.66 | 10.93 |
| Chongqing | 10.49 | 10.31 | 10.82 | 10.45 | 11.60 | 10.33 | 12.88 | 10.15 | 11.88 | 6.08 | 9.74 |
| Sichuan | 10.93 | 10.41 | 11.17 | 10.95 | 11.64 | 11.22 | 12.27 | 9.56 | 8.56 | 7.66 | 9.77 |
| Guizhou | 10.66 | 11.73 | 10.37 | 11.12 | 12.28 | 11.02 | 13.04 | 11.09 | 10.09 | 5.83 | 8.47 |
| Yunnan | 10.73 | 11.34 | 11.35 | 11.19 | 12.11 | 10.04 | 11.44 | 11.60 | 10.15 | 8.52 | 9.58 |
| Xizang | 9.75 | 9.42 | 9.94 | 10.51 | 11.60 | 9.61 | 11.95 | 10.63 | 16.15 | 9.58 | 9.87 |
| Shaanxi | 10.56 | 11.64 | 11.15 | 10.48 | 13.17 | 9.61 | 14.11 | 11.36 | 11.05 | 6.31 | 8.61 |
| Gansu | 10.02 | 9.67 | 11.45 | 11.38 | 12.24 | 9.82 | 12.71 | 10.75 | 7.59 | 5.83 | 10.37 |
| Qinghai | 10.47 | 9.33 | 13.46 | 12.98 | 13.75 | 9.78 | 13.21 | 15.61 | 11.76 | 5.95 | 8.31 |
| Ningxia | 11.82 | 9.44 | 12.72 | 10.76 | 12.46 | 9.59 | 15.61 | 14.92 | 11.23 | 7.12 | 8.60 |
| Xinjiang | 9.48 | 9.85 | 11.41 | 11.33 | 11.67 | 10.07 | 14.28 | 12.46 | 12.64 | 9.14 | 7.26 |
| **Province** | **1963** | **1964** | **1965** | **1966** | **1967** | **1968** | **1969** | **1970** | **1971** | **1972** | **1973** |
| Beijing | 10.25 | 11.43 | 11.96 | 11.54 | 8.33 | 10.16 | 14.72 | 15.90 | 11.58 | 8.67 | 11.43 |
| Tianjin | 10.59 | 11.55 | 12.55 | 10.97 | 8.30 | 11.19 | 12.91 | 12.64 | 11.29 | 10.75 | 11.38 |
| Hebei | 9.09 | 12.72 | 12.71 | 11.66 | 9.30 | 10.46 | 12.34 | 12.63 | 10.85 | 9.99 | 11.51 |
| Shanxi | 9.55 | 13.47 | 12.90 | 12.70 | 10.16 | 9.18 | 11.41 | 12.62 | 10.82 | 10.85 | 11.01 |
| Neimenggu | 11.78 | 11.25 | 11.19 | 11.42 | 7.79 | 10.24 | 10.33 | 13.35 | 10.47 | 11.16 | 11.85 |
| Liaoning | 10.55 | 11.66 | 12.70 | 11.74 | 7.81 | 10.24 | 13.78 | 13.51 | 11.45 | 10.65 | 12.02 |
| Jinlin | 11.39 | 10.48 | 11.60 | 11.22 | 8.83 | 9.95 | 11.23 | 13.78 | 11.24 | 9.17 | 12.01 |
| Heilongjiang | 11.28 | 11.41 | 11.84 | 12.20 | 9.81 | 9.95 | 11.36 | 11.74 | 10.52 | 10.13 | 11.20 |
| Shanghai | 10.97 | 11.61 | 12.68 | 11.71 | 8.73 | 11.91 | 12.01 | 11.99 | 10.94 | 10.53 | 11.62 |
| Jiangsu | 11.14 | 11.61 | 10.88 | 12.28 | 8.74 | 10.58 | 11.07 | 12.33 | 11.79 | 10.86 | 11.41 |
| Zhejiang | 10.74 | 11.34 | 11.25 | 10.48 | 9.29 | 9.81 | 11.94 | 11.78 | 10.75 | 12.32 | 10.75 |
| Anhui | 10.01 | 11.61 | 12.15 | 10.96 | 9.71 | 10.25 | 10.04 | 13.31 | 11.10 | 10.79 | 11.01 |
| Fujian | 10.02 | 11.66 | 11.32 | 11.61 | 8.64 | 8.94 | 12.62 | 11.72 | 11.88 | 11.23 | 10.24 |
| Jiangxi | 9.82 | 10.61 | 11.82 | 11.00 | 9.11 | 10.72 | 11.82 | 11.46 | 11.21 | 10.78 | 10.37 |
| Shandong | 10.81 | 11.05 | 12.61 | 12.16 | 9.91 | 10.13 | 11.16 | 12.42 | 12.07 | 11.23 | 11.10 |
| Henan | 9.55 | 13.47 | 12.90 | 12.70 | 10.16 | 9.18 | 11.41 | 12.62 | 10.82 | 10.85 | 11.01 |
| Hubei | 10.70 | 11.09 | 12.15 | 10.63 | 9.01 | 9.33 | 10.68 | 13.78 | 11.85 | 10.46 | 11.65 |
| Hunan | 9.19 | 11.88 | 11.57 | 11.75 | 9.69 | 10.08 | 11.49 | 12.65 | 10.88 | 11.18 | 11.26 |
| Guangdong | 11.16 | 11.35 | 11.39 | 10.66 | 9.68 | 9.09 | 12.03 | 11.74 | 10.51 | 10.72 | 11.16 |
| Guangxi | 10.25 | 10.52 | 11.20 | 10.27 | 9.51 | 8.96 | 13.39 | 12.31 | 12.18 | 12.01 | 11.03 |
| Hainan | 11.16 | 11.35 | 11.39 | 10.66 | 9.68 | 9.09 | 12.03 | 11.74 | 10.51 | 10.72 | 11.16 |
| Chongqing | 11.21 | 11.42 | 11.71 | 10.91 | 8.54 | 8.38 | 11.63 | 13.01 | 11.59 | 10.22 | 10.80 |
| Sichuan | 10.94 | 11.16 | 11.67 | 11.74 | 9.62 | 8.67 | 11.98 | 11.84 | 10.72 | 9.74 | 11.19 |
| Guizhou | 10.55 | 11.72 | 12.34 | 9.93 | 9.17 | 9.09 | 10.12 | 14.81 | 13.18 | 9.75 | 10.19 |
| Yunnan | 10.08 | 11.40 | 11.97 | 11.38 | 8.77 | 7.39 | 14.05 | 12.29 | 11.14 | 11.74 | 11.67 |
| Xizang | 10.21 | 10.96 | 11.92 | 11.12 | 10.42 | 9.48 | 10.10 | 11.22 | 11.24 | 10.98 | 11.44 |
| Shaanxi | 10.62 | 11.11 | 13.77 | 11.45 | 8.71 | 7.38 | 15.59 | 13.49 | 12.40 | 10.56 | 10.75 |
| Gansu | 12.62 | 12.42 | 12.92 | 10.06 | 7.95 | 11.70 | 12.39 | 13.24 | 11.19 | 10.92 | 11.20 |
| Qinghai | 10.60 | 11.14 | 11.50 | 11.07 | 10.41 | 9.41 | 11.70 | 12.89 | 10.90 | 12.07 | 11.55 |
| Ningxia | 11.73 | 10.88 | 12.00 | 12.15 | 9.10 | 9.80 | 13.00 | 12.81 | 12.05 | 11.33 | 10.84 |
| Xinjiang | 10.87 | 11.26 | 11.68 | 11.64 | 7.82 | 9.11 | 10.46 | 12.65 | 11.17 | 9.60 | 10.81 |
| **Province** | **1974** | **1975** | **1976** | **1977** | **1978** | **1979** | **1980** | **1981** | **1982** | **1983** | **1984** |
| Beijing | 11.62 | 12.10 | 10.83 | 10.97 | 11.36 | 10.67 | 10.60 | 9.11 | 10.01 | 11.12 | 11.54 |
| Tianjin | 11.73 | 11.02 | 9.72 | 10.59 | 12.58 | 10.71 | 10.41 | 9.67 | 9.68 | 10.24 | 11.75 |
| Hebei | 11.48 | 11.77 | 11.03 | 12.09 | 11.83 | 10.28 | 9.67 | 9.27 | 10.47 | 10.59 | 11.20 |
| Shanxi | 10.71 | 11.26 | 10.34 | 11.37 | 11.46 | 10.56 | 10.99 | 9.99 | 9.68 | 11.91 | 10.73 |
| Neimenggu | 10.00 | 11.87 | 10.36 | 10.99 | 11.07 | 10.68 | 9.51 | 10.29 | 11.20 | 10.40 | 11.39 |
| Liaoning | 11.44 | 11.50 | 10.84 | 10.23 | 11.39 | 10.14 | 10.32 | 9.00 | 9.78 | 10.78 | 11.47 |
| Jinlin | 10.74 | 12.05 | 9.65 | 11.01 | 11.63 | 10.22 | 10.03 | 9.78 | 10.04 | 11.69 | 10.99 |
| Heilongjiang | 11.26 | 11.42 | 10.55 | 11.13 | 11.43 | 9.93 | 10.41 | 9.57 | 9.92 | 10.27 | 10.84 |
| Shanghai | 11.78 | 11.00 | 10.60 | 11.29 | 11.98 | 10.42 | 10.23 | 9.76 | 9.98 | 10.19 | 10.89 |
| Jiangsu | 10.58 | 11.24 | 10.55 | 10.91 | 13.01 | 10.93 | 9.85 | 10.32 | 10.26 | 10.67 | 11.35 |
| Zhejiang | 10.35 | 10.08 | 10.92 | 11.93 | 12.70 | 11.11 | 11.10 | 10.38 | 10.43 | 10.21 | 12.01 |
| Anhui | 10.50 | 10.92 | 11.47 | 9.97 | 10.18 | 10.70 | 9.59 | 11.05 | 10.22 | 10.29 | 11.84 |
| Fujian | 10.90 | 10.85 | 10.42 | 12.02 | 12.22 | 10.21 | 11.31 | 10.81 | 10.21 | 10.01 | 11.59 |
| Jiangxi | 10.00 | 11.70 | 9.57 | 11.65 | 11.69 | 11.35 | 9.78 | 9.76 | 10.21 | 10.08 | 11.31 |
| Shandong | 8.55 | 14.04 | 11.06 | 11.50 | 11.32 | 10.33 | 10.64 | 9.78 | 10.42 | 10.85 | 11.54 |
| Henan | 10.71 | 11.26 | 10.34 | 11.37 | 11.46 | 10.56 | 10.99 | 9.99 | 9.68 | 11.91 | 10.73 |
| Hubei | 9.59 | 11.88 | 9.81 | 12.03 | 11.71 | 11.33 | 10.02 | 9.86 | 10.48 | 9.98 | 11.92 |
| Hunan | 9.57 | 11.76 | 10.37 | 11.30 | 12.05 | 10.61 | 9.89 | 9.75 | 10.22 | 10.34 | 10.65 |
| Guangdong | 11.26 | 11.82 | 10.99 | 11.05 | 10.25 | 10.54 | 11.12 | 10.12 | 10.50 | 10.13 | 11.34 |
| Guangxi | 11.11 | 11.33 | 10.57 | 10.76 | 11.50 | 9.97 | 10.43 | 10.01 | 10.55 | 9.70 | 10.37 |
| Hainan | 11.26 | 11.82 | 10.99 | 11.05 | 10.25 | 10.54 | 9.66 | 9.36 | 10.60 | 11.89 | 10.25 |
| Chongqing | 10.63 | 11.90 | 9.82 | 12.49 | 12.10 | 10.81 | 10.14 | 9.80 | 10.14 | 10.43 | 11.35 |
| Sichuan | 10.40 | 12.54 | 11.52 | 12.05 | 12.17 | 10.72 | 10.35 | 9.60 | 10.38 | 10.53 | 10.96 |
| Guizhou | 9.23 | 11.79 | 9.64 | 13.52 | 12.91 | 10.82 | 9.80 | 9.86 | 10.90 | 10.70 | 11.80 |
| Yunnan | 10.01 | 11.13 | 9.30 | 11.77 | 12.67 | 9.94 | 10.24 | 9.99 | 10.87 | 10.25 | 11.21 |
| Xizang | 11.19 | 11.14 | 11.09 | 11.01 | 10.98 | 10.46 | 11.74 | 11.34 | 9.25 | 8.78 | 12.41 |
| Shaanxi | 10.90 | 11.11 | 10.14 | 11.62 | 11.42 | 10.43 | 10.12 | 9.64 | 10.19 | 10.13 | 11.58 |
| Gansu | 12.12 | 12.49 | 10.47 | 10.31 | 11.68 | 9.75 | 10.31 | 8.28 | 10.17 | 10.95 | 11.14 |
| Qinghai | 10.92 | 11.45 | 10.47 | 11.08 | 11.88 | 8.59 | 11.25 | 9.02 | 10.47 | 10.49 | 11.11 |
| Ningxia | 12.94 | 11.18 | 9.92 | 10.99 | 11.17 | 10.31 | 10.18 | 9.38 | 10.17 | 11.01 | 11.11 |
| Xinjiang | 9.69 | 12.54 | 11.18 | 12.20 | 11.28 | 10.97 | 10.12 | 10.07 | 10.27 | 10.80 | 11.17 |
| **Province** | **1974** | **1975** | **1976** | **1977** | **1978** | **1979** | **1980** | **1981** | **1982** | **1983** | **1984** |
| Beijing | 11.62 | 12.10 | 10.83 | 10.97 | 11.36 | 10.67 | 10.60 | 9.11 | 10.01 | 11.12 | 11.54 |
| Tianjin | 11.73 | 11.02 | 9.72 | 10.59 | 12.58 | 10.71 | 10.41 | 9.67 | 9.68 | 10.24 | 11.75 |
| Hebei | 11.48 | 11.77 | 11.03 | 12.09 | 11.83 | 10.28 | 9.67 | 9.27 | 10.47 | 10.59 | 11.20 |
| Shanxi | 10.71 | 11.26 | 10.34 | 11.37 | 11.46 | 10.56 | 10.99 | 9.99 | 9.68 | 11.91 | 10.73 |
| Neimenggu | 10.00 | 11.87 | 10.36 | 10.99 | 11.07 | 10.68 | 9.51 | 10.29 | 11.20 | 10.40 | 11.39 |
| Liaoning | 11.44 | 11.50 | 10.84 | 10.23 | 11.39 | 10.14 | 10.32 | 9.00 | 9.78 | 10.78 | 11.47 |
| Jinlin | 10.74 | 12.05 | 9.65 | 11.01 | 11.63 | 10.22 | 10.03 | 9.78 | 10.04 | 11.69 | 10.99 |
| Heilongjiang | 11.26 | 11.42 | 10.55 | 11.13 | 11.43 | 9.93 | 10.41 | 9.57 | 9.92 | 10.27 | 10.84 |
| Shanghai | 11.78 | 11.00 | 10.60 | 11.29 | 11.98 | 10.42 | 10.23 | 9.76 | 9.98 | 10.19 | 10.89 |
| Jiangsu | 10.58 | 11.24 | 10.55 | 10.91 | 13.01 | 10.93 | 9.85 | 10.32 | 10.26 | 10.67 | 11.35 |
| Zhejiang | 10.35 | 10.08 | 10.92 | 11.93 | 12.70 | 11.11 | 11.10 | 10.38 | 10.43 | 10.21 | 12.01 |
| Anhui | 10.50 | 10.92 | 11.47 | 9.97 | 10.18 | 10.70 | 9.59 | 11.05 | 10.22 | 10.29 | 11.84 |
| Fujian | 10.90 | 10.85 | 10.42 | 12.02 | 12.22 | 10.21 | 11.31 | 10.81 | 10.21 | 10.01 | 11.59 |
| Jiangxi | 10.00 | 11.70 | 9.57 | 11.65 | 11.69 | 11.35 | 9.78 | 9.76 | 10.21 | 10.08 | 11.31 |
| Shandong | 8.55 | 14.04 | 11.06 | 11.50 | 11.32 | 10.33 | 10.64 | 9.78 | 10.42 | 10.85 | 11.54 |
| Henan | 10.71 | 11.26 | 10.34 | 11.37 | 11.46 | 10.56 | 10.99 | 9.99 | 9.68 | 11.91 | 10.73 |
| Hubei | 9.59 | 11.88 | 9.81 | 12.03 | 11.71 | 11.33 | 10.02 | 9.86 | 10.48 | 9.98 | 11.92 |
| Hunan | 9.57 | 11.76 | 10.37 | 11.30 | 12.05 | 10.61 | 9.89 | 9.75 | 10.22 | 10.34 | 10.65 |
| Guangdong | 11.26 | 11.82 | 10.99 | 11.05 | 10.25 | 10.54 | 11.12 | 10.12 | 10.50 | 10.13 | 11.34 |
| Guangxi | 11.11 | 11.33 | 10.57 | 10.76 | 11.50 | 9.97 | 10.43 | 10.01 | 10.55 | 9.70 | 10.37 |
| Hainan | 11.26 | 11.82 | 10.99 | 11.05 | 10.25 | 10.54 | 9.66 | 9.36 | 10.60 | 11.89 | 10.25 |
| Chongqing | 10.63 | 11.90 | 9.82 | 12.49 | 12.10 | 10.81 | 10.14 | 9.80 | 10.14 | 10.43 | 11.35 |
| Sichuan | 10.40 | 12.54 | 11.52 | 12.05 | 12.17 | 10.72 | 10.35 | 9.60 | 10.38 | 10.53 | 10.96 |
| Guizhou | 9.23 | 11.79 | 9.64 | 13.52 | 12.91 | 10.82 | 9.80 | 9.86 | 10.90 | 10.70 | 11.80 |
| Yunnan | 10.01 | 11.13 | 9.30 | 11.77 | 12.67 | 9.94 | 10.24 | 9.99 | 10.87 | 10.25 | 11.21 |
| Xizang | 11.19 | 11.14 | 11.09 | 11.01 | 10.98 | 10.46 | 11.74 | 11.34 | 9.25 | 8.78 | 12.41 |
| Shaanxi | 10.90 | 11.11 | 10.14 | 11.62 | 11.42 | 10.43 | 10.12 | 9.64 | 10.19 | 10.13 | 11.58 |
| Gansu | 12.12 | 12.49 | 10.47 | 10.31 | 11.68 | 9.75 | 10.31 | 8.28 | 10.17 | 10.95 | 11.14 |
| Qinghai | 10.92 | 11.45 | 10.47 | 11.08 | 11.88 | 8.59 | 11.25 | 9.02 | 10.47 | 10.49 | 11.11 |
| Ningxia | 12.94 | 11.18 | 9.92 | 10.99 | 11.17 | 10.31 | 10.18 | 9.38 | 10.17 | 11.01 | 11.11 |
| Xinjiang | 9.69 | 12.54 | 11.18 | 12.20 | 11.28 | 10.97 | 10.12 | 10.07 | 10.27 | 10.80 | 11.17 |
| **Province** | **1985** | **1986** | **1987** | **1988** | **1989** | **1990** | **1991** | **1992** | **1993** | **1994** | **1995** |
| Beijing | 10.64 | 10.47 | 10.67 | 11.04 | 9.88 | 10.03 | 10.61 | 10.82 | 10.90 | 11.04 | 10.43 |
| Tianjin | 10.85 | 10.22 | 10.45 | 10.26 | 9.57 | 10.06 | 10.18 | 10.87 | 10.88 | 11.10 | 10.74 |
| Hebei | 11.06 | 10.15 | 10.90 | 11.12 | 10.06 | 10.10 | 10.73 | 11.30 | 11.50 | 11.17 | 10.63 |
| Shanxi | 11.18 | 10.09 | 11.27 | 10.71 | 10.16 | 9.96 | 10.28 | 11.09 | 11.29 | 11.05 | 10.73 |
| Neimenggu | 11.59 | 10.24 | 10.61 | 10.71 | 9.69 | 10.29 | 10.35 | 10.79 | 10.84 | 10.76 | 10.23 |
| Liaoning | 11.15 | 10.50 | 11.17 | 10.92 | 9.73 | 9.56 | 10.19 | 10.91 | 11.19 | 10.76 | 9.91 |
| Jinlin | 10.43 | 10.39 | 11.70 | 11.39 | 9.13 | 9.84 | 10.17 | 10.92 | 10.95 | 10.60 | 10.19 |
| Heilongjiang | 10.34 | 9.97 | 10.56 | 10.57 | 10.08 | 10.10 | 10.25 | 10.29 | 10.36 | 10.45 | 10.14 |
| Shanghai | 11.16 | 10.07 | 10.44 | 10.74 | 9.72 | 9.85 | 10.31 | 11.21 | 11.21 | 11.12 | 10.68 |
| Jiangsu | 11.60 | 10.73 | 11.10 | 11.80 | 9.67 | 10.01 | 10.44 | 12.41 | 11.73 | 11.34 | 10.79 |
| Zhejiang | 12.09 | 10.92 | 10.92 | 10.86 | 9.33 | 9.89 | 11.48 | 11.68 | 11.97 | 11.73 | 10.94 |
| Anhui | 11.39 | 10.81 | 10.14 | 10.22 | 9.96 | 9.78 | 9.42 | 11.43 | 11.60 | 11.12 | 10.68 |
| Fujian | 11.63 | 10.21 | 11.12 | 11.21 | 10.24 | 10.29 | 11.09 | 11.82 | 12.04 | 11.76 | 10.71 |
| Jiangxi | 11.32 | 10.32 | 10.53 | 10.89 | 10.06 | 9.96 | 10.43 | 11.21 | 11.06 | 10.50 | 9.88 |
| Shandong | 10.94 | 10.28 | 11.14 | 11.01 | 9.83 | 10.05 | 11.13 | 11.44 | 11.80 | 11.31 | 10.65 |
| Henan | 11.18 | 10.09 | 11.27 | 10.71 | 10.16 | 9.96 | 10.28 | 11.09 | 11.29 | 11.05 | 10.73 |
| Hubei | 11.48 | 10.19 | 10.54 | 10.48 | 9.89 | 10.01 | 10.25 | 11.13 | 10.98 | 11.04 | 10.56 |
| Hunan | 11.01 | 10.48 | 10.64 | 10.53 | 9.79 | 9.90 | 10.39 | 10.80 | 10.91 | 10.70 | 10.25 |
| Guangdong | 11.68 | 10.99 | 11.79 | 11.37 | 10.18 | 10.73 | 11.47 | 12.02 | 12.08 | 11.70 | 10.81 |
| Guangxi | 10.90 | 10.29 | 10.63 | 10.12 | 9.79 | 10.23 | 10.92 | 11.60 | 11.56 | 11.20 | 10.37 |
| Hainan | 11.56 | 10.91 | 10.57 | 10.90 | 10.45 | 10.09 | 10.69 | 11.22 | 14.12 | 11.80 | 10.36 |
| Chongqing | 10.61 | 10.51 | 10.17 | 10.65 | 9.91 | 10.21 | 10.51 | 11.37 | 11.23 | 10.99 | 10.44 |
| Sichuan | 11.00 | 10.19 | 10.57 | 10.45 | 9.75 | 10.46 | 10.53 | 10.97 | 10.99 | 10.77 | 10.30 |
| Guizhou | 10.55 | 10.20 | 10.81 | 10.57 | 9.89 | 9.94 | 10.54 | 10.47 | 10.69 | 10.45 | 9.96 |
| Yunnan | 11.12 | 10.06 | 10.97 | 11.40 | 10.03 | 10.42 | 10.25 | 10.78 | 10.77 | 10.87 | 10.40 |
| Xizang | 11.39 | 8.56 | 9.62 | 10.08 | 10.31 | 10.44 | 9.57 | 10.36 | 11.25 | 11.26 | 11.06 |
| Shaanxi | 11.51 | 10.55 | 10.72 | 11.95 | 9.76 | 9.84 | 10.32 | 10.49 | 10.88 | 10.47 | 10.26 |
| Gansu | 11.14 | 10.80 | 10.60 | 11.14 | 10.35 | 10.08 | 10.25 | 10.67 | 10.82 | 10.72 | 10.26 |
| Qinghai | 10.88 | 10.48 | 10.24 | 10.47 | 9.53 | 9.87 | 10.04 | 10.39 | 10.61 | 10.42 | 10.01 |
| Ningxia | 11.66 | 10.50 | 10.49 | 10.96 | 10.24 | 9.87 | 10.05 | 10.52 | 10.68 | 10.38 | 10.17 |
| Xinjiang | 11.56 | 10.88 | 10.72 | 10.68 | 10.06 | 10.75 | 11.11 | 11.02 | 10.67 | 10.86 | 10.13 |
| **Province** | **1996** | **1997** | **1998** | **1999** | **2000** | **2001** | **2002** | **2003** | **2004** | **2005** | **2006** |
| Beijing | 10.13 | 10.57 | 10.39 | 10.59 | 10.72 | 10.73 | 10.64 | 10.62 | 11.01 | 10.82 | 10.84 |
| Tianjin | 10.69 | 10.79 | 10.37 | 10.49 | 10.61 | 10.76 | 10.77 | 11.04 | 11.19 | 11.13 | 11.02 |
| Hebei | 10.60 | 10.83 | 10.52 | 10.40 | 10.47 | 10.40 | 10.43 | 10.69 | 10.88 | 10.99 | 10.90 |
| Shanxi | 10.65 | 10.60 | 10.32 | 10.29 | 10.47 | 10.43 | 10.42 | 10.59 | 10.96 | 11.08 | 11.01 |
| Neimenggu | 10.70 | 10.64 | 10.52 | 10.36 | 10.61 | 10.62 | 10.83 | 11.38 | 11.71 | 12.14 | 11.52 |
| Liaoning | 10.08 | 10.44 | 10.26 | 10.30 | 10.40 | 10.43 | 10.50 | 10.68 | 10.86 | 10.87 | 10.95 |
| Jinlin | 10.60 | 10.45 | 10.35 | 10.30 | 10.43 | 10.47 | 10.42 | 10.54 | 10.80 | 10.85 | 11.08 |
| Heilongjiang | 10.25 | 10.56 | 10.26 | 10.22 | 10.32 | 10.47 | 10.50 | 10.54 | 10.74 | 10.79 | 10.76 |
| Shanghai | 10.56 | 10.86 | 10.48 | 10.54 | 10.63 | 10.60 | 10.62 | 10.77 | 11.02 | 10.74 | 10.75 |
| Jiangsu | 10.47 | 10.77 | 10.56 | 10.50 | 10.59 | 10.56 | 10.66 | 10.91 | 11.08 | 11.11 | 11.07 |
| Zhejiang | 10.52 | 10.68 | 10.47 | 10.49 | 10.63 | 10.61 | 10.76 | 11.03 | 11.05 | 10.93 | 10.96 |
| Anhui | 10.52 | 10.74 | 10.26 | 10.40 | 10.34 | 10.42 | 10.43 | 10.45 | 10.92 | 10.79 | 10.84 |
| Fujian | 10.58 | 10.99 | 10.53 | 10.48 | 10.44 | 10.40 | 10.50 | 10.68 | 10.76 | 10.79 | 11.06 |
| Jiangxi | 10.41 | 10.81 | 10.13 | 10.25 | 10.30 | 10.41 | 10.53 | 10.84 | 10.91 | 10.93 | 10.78 |
| Shandong | 10.46 | 10.68 | 10.53 | 10.49 | 10.55 | 10.54 | 10.66 | 10.89 | 11.14 | 11.19 | 11.06 |
| Henan | 10.65 | 10.60 | 10.32 | 10.29 | 10.47 | 10.43 | 10.42 | 10.59 | 10.96 | 11.08 | 11.01 |
| Hubei | 10.40 | 10.76 | 10.30 | 10.25 | 10.37 | 10.42 | 10.39 | 10.48 | 10.69 | 10.85 | 10.88 |
| Hunan | 10.46 | 10.62 | 10.29 | 10.32 | 10.41 | 10.43 | 10.37 | 10.47 | 10.79 | 10.79 | 10.77 |
| Guangdong | 10.37 | 10.69 | 10.53 | 10.50 | 10.68 | 10.60 | 10.74 | 11.04 | 11.08 | 11.04 | 11.04 |
| Guangxi | 10.05 | 10.34 | 10.45 | 10.28 | 10.29 | 10.36 | 10.54 | 10.54 | 10.76 | 10.97 | 10.93 |
| Hainan | 9.58 | 9.98 | 10.10 | 10.33 | 10.36 | 10.43 | 10.38 | 10.47 | 10.62 | 10.69 | 10.55 |
| Chongqing | 10.36 | 10.66 | 10.27 | 10.23 | 10.36 | 10.43 | 10.51 | 10.68 | 10.80 | 10.78 | 10.77 |
| Sichuan | 10.30 | 10.61 | 10.42 | 10.12 | 10.36 | 10.43 | 10.51 | 10.66 | 10.85 | 10.90 | 10.89 |
| Guizhou | 10.12 | 10.45 | 10.29 | 10.36 | 10.35 | 10.41 | 10.38 | 10.53 | 10.71 | 10.79 | 10.71 |
| Yunnan | 10.35 | 10.52 | 10.24 | 10.20 | 10.25 | 10.19 | 10.37 | 10.38 | 10.70 | 10.51 | 10.74 |
| Xizang | 10.57 | 10.75 | 10.68 | 10.74 | 10.56 | 10.84 | 10.79 | 10.73 | 10.79 | 10.85 | 10.89 |
| Shaanxi | 10.33 | 10.63 | 10.62 | 10.53 | 10.56 | 10.52 | 10.60 | 10.71 | 10.88 | 10.90 | 10.84 |
| Gansu | 10.44 | 10.46 | 10.42 | 10.39 | 10.49 | 10.52 | 10.47 | 10.59 | 10.72 | 10.82 | 10.70 |
| Qinghai | 10.10 | 10.45 | 10.33 | 10.29 | 10.41 | 10.73 | 10.70 | 10.72 | 10.81 | 10.86 | 10.77 |
| Ningxia | 10.33 | 10.33 | 10.32 | 10.40 | 10.54 | 10.55 | 10.50 | 10.81 | 10.69 | 10.72 | 10.83 |
| Xinjiang | 9.86 | 10.38 | 10.18 | 10.21 | 10.38 | 10.39 | 10.28 | 10.65 | 10.71 | 10.72 | 10.64 |
| **Province** | **2007** | **2008** | **2009** | **2010** | **2011** | **2012** | **2013** | **2014** | **2015** | **2016** | **2017** |
| Beijing | 10.87 | 10.45 | 10.47 | 10.45 | 10.03 | 9.94 | 9.87 | 9.74 | 9.70 | 9.57 | 9.49 |
| Tianjin | 11.08 | 11.27 | 11.15 | 11.22 | 10.91 | 10.58 | 10.38 | 10.02 | 9.95 | 9.81 | 9.17 |
| Hebei | 10.82 | 10.57 | 10.45 | 10.66 | 10.37 | 10.14 | 9.93 | 9.66 | 9.69 | 9.57 | 9.48 |
| Shanxi | 11.01 | 10.79 | 10.55 | 10.69 | 10.43 | 10.19 | 10.01 | 9.91 | 9.84 | 9.70 | 9.60 |
| Neimenggu | 11.50 | 11.35 | 11.19 | 10.96 | 10.68 | 10.34 | 10.01 | 9.79 | 9.78 | 9.61 | 9.21 |
| Liaoning | 11.00 | 10.90 | 10.78 | 10.87 | 10.46 | 10.13 | 9.98 | 9.58 | 9.29 | 8.62 | 9.23 |
| Jinlin | 11.18 | 11.21 | 10.84 | 10.83 | 10.63 | 10.39 | 9.94 | 9.66 | 9.63 | 9.58 | 9.34 |
| Heilongjiang | 10.73 | 10.76 | 10.60 | 10.71 | 10.47 | 10.18 | 9.91 | 9.56 | 9.57 | 9.50 | 9.46 |
| Shanghai | 10.98 | 10.53 | 10.25 | 10.45 | 10.04 | 9.92 | 9.87 | 9.71 | 9.70 | 9.58 | 9.51 |
| Jiangsu | 11.05 | 10.81 | 10.71 | 10.71 | 10.33 | 10.19 | 10.07 | 9.88 | 9.86 | 9.67 | 9.54 |
| Zhejiang | 11.03 | 10.57 | 10.33 | 10.63 | 10.12 | 9.97 | 9.93 | 9.77 | 9.81 | 9.65 | 9.60 |
| Anhui | 10.94 | 10.85 | 10.76 | 10.92 | 10.60 | 10.40 | 10.16 | 9.94 | 9.88 | 9.76 | 9.67 |
| Fujian | 11.08 | 10.89 | 10.70 | 10.84 | 10.47 | 10.33 | 10.22 | 10.01 | 9.91 | 9.73 | 9.63 |
| Jiangxi | 10.84 | 10.84 | 10.78 | 10.85 | 10.49 | 10.29 | 10.13 | 9.99 | 9.93 | 9.79 | 9.70 |
| Shandong | 10.98 | 10.79 | 10.69 | 10.67 | 10.32 | 10.16 | 10.07 | 9.88 | 9.81 | 9.65 | 9.56 |
| Henan | 11.01 | 10.79 | 10.55 | 10.69 | 10.43 | 10.19 | 10.01 | 9.91 | 9.84 | 9.70 | 9.60 |
| Hubei | 11.00 | 10.93 | 10.83 | 10.94 | 10.63 | 10.32 | 10.13 | 9.99 | 9.90 | 9.70 | 9.60 |
| Hunan | 11.00 | 10.86 | 10.85 | 10.92 | 10.53 | 10.32 | 10.13 | 9.97 | 9.86 | 9.69 | 9.62 |
| Guangdong | 11.03 | 10.57 | 10.42 | 10.68 | 10.23 | 9.99 | 9.96 | 9.79 | 9.81 | 9.64 | 9.57 |
| Guangxi | 11.07 | 10.86 | 10.87 | 10.87 | 10.47 | 10.32 | 10.14 | 9.86 | 9.82 | 9.62 | 9.53 |
| Hainan | 10.79 | 11.08 | 10.43 | 10.60 | 10.86 | 10.39 | 10.02 | 10.01 | 9.86 | 9.67 | 9.57 |
| Chongqing | 11.12 | 11.03 | 10.98 | 11.19 | 10.91 | 10.56 | 10.36 | 10.11 | 10.12 | 9.97 | 9.75 |
| Sichuan | 10.97 | 10.50 | 10.94 | 10.97 | 10.76 | 10.46 | 10.12 | 9.86 | 9.80 | 9.67 | 9.63 |
| Guizhou | 10.92 | 10.58 | 10.60 | 10.72 | 10.76 | 10.56 | 10.38 | 10.10 | 10.09 | 9.95 | 9.84 |
| Yunnan | 10.79 | 10.67 | 10.68 | 10.67 | 10.62 | 10.50 | 10.34 | 9.82 | 9.88 | 9.76 | 9.77 |
| Xizang | 10.95 | 10.57 | 10.71 | 10.67 | 10.52 | 10.37 | 10.34 | 10.10 | 10.12 | 9.91 | 9.82 |
| Shaanxi | 11.01 | 11.17 | 10.84 | 10.92 | 10.64 | 10.49 | 10.22 | 9.99 | 9.80 | 9.65 | 9.62 |
| Gansu | 10.76 | 10.57 | 10.48 | 10.61 | 10.49 | 10.46 | 10.20 | 9.91 | 9.82 | 9.65 | 9.17 |
| Qinghai | 10.79 | 10.85 | 10.46 | 10.99 | 10.60 | 10.42 | 10.20 | 9.94 | 9.83 | 9.69 | 9.55 |
| Ningxia | 10.81 | 10.80 | 10.65 | 10.80 | 10.45 | 10.34 | 10.09 | 9.81 | 9.81 | 9.70 | 9.60 |
| Xinjiang | 10.75 | 10.67 | 10.24 | 10.48 | 10.44 | 10.39 | 10.22 | 10.02 | 9.89 | 9.65 | 9.58 |
| **Province** | **2018** | **2019** | **2020** | **2021** | **2022** |  |  |  |  |  |  |
| Beijing | 9.47 | 9.32 | 8.68 | 9.25 | 8.48 |  |  |  |  |  |  |
| Tianjin | 9.17 | 9.19 | 8.71 | 9.07 | 8.50 |  |  |  |  |  |  |
| Hebei | 9.47 | 9.39 | 8.95 | 9.06 | 8.78 |  |  |  |  |  |  |
| Shanxi | 9.57 | 9.41 | 8.69 | 9.04 | 8.83 |  |  |  |  |  |  |
| Neimenggu | 9.34 | 9.23 | 8.58 | 9.04 | 8.82 |  |  |  |  |  |  |
| Liaoning | 9.38 | 9.26 | 8.62 | 8.99 | 8.61 |  |  |  |  |  |  |
| Jinlin | 9.26 | 9.01 | 8.80 | 9.07 | 8.22 |  |  |  |  |  |  |
| Heilongjiang | 9.28 | 9.13 | 8.66 | 9.02 | 8.67 |  |  |  |  |  |  |
| Shanghai | 9.47 | 9.31 | 8.73 | 9.22 | 8.39 |  |  |  |  |  |  |
| Jiangsu | 9.48 | 9.32 | 8.93 | 9.26 | 8.68 |  |  |  |  |  |  |
| Zhejiang | 9.52 | 9.39 | 8.92 | 9.25 | 8.71 |  |  |  |  |  |  |
| Anhui | 9.61 | 9.46 | 8.95 | 9.23 | 8.75 |  |  |  |  |  |  |
| Fujian | 9.64 | 9.47 | 8.89 | 9.21 | 8.86 |  |  |  |  |  |  |
| Jiangxi | 9.68 | 9.51 | 8.94 | 9.28 | 8.86 |  |  |  |  |  |  |
| Shandong | 9.45 | 9.26 | 8.92 | 9.23 | 8.79 |  |  |  |  |  |  |
| Henan | 9.57 | 9.41 | 8.69 | 9.04 | 8.71 |  |  |  |  |  |  |
| Hubei | 9.59 | 9.46 | 8.07 | 9.68 | 8.83 |  |  |  |  |  |  |
| Hunan | 9.59 | 9.47 | 8.94 | 9.18 | 8.84 |  |  |  |  |  |  |
| Guangdong | 9.49 | 9.33 | 8.79 | 9.21 | 8.59 |  |  |  |  |  |  |
| Guangxi | 9.49 | 9.31 | 8.93 | 9.16 | 8.69 |  |  |  |  |  |  |
| Hainan | 9.39 | 9.29 | 8.91 | 9.52 | 8.43 |  |  |  |  |  |  |
| Chongqing | 9.41 | 9.34 | 8.95 | 9.23 | 8.66 |  |  |  |  |  |  |
| Sichuan | 9.61 | 9.46 | 8.94 | 9.22 | 8.69 |  |  |  |  |  |  |
| Guizhou | 9.72 | 9.55 | 9.01 | 9.22 | 8.52 |  |  |  |  |  |  |
| Yunnan | 9.70 | 9.52 | 8.96 | 9.14 | 8.83 |  |  |  |  |  |  |
| Xizang | 9.72 | 9.52 | 9.33 | 9.08 | 8.51 |  |  |  |  |  |  |
| Shaanxi | 9.64 | 9.31 | 8.78 | 9.06 | 8.83 |  |  |  |  |  |  |
| Gansu | 9.44 | 9.33 | 8.95 | 9.10 | 8.84 |  |  |  |  |  |  |
| Qinghai | 9.53 | 9.34 | 8.71 | 8.98 | 8.63 |  |  |  |  |  |  |
| Ningxia | 9.51 | 9.36 | 8.95 | 9.08 | 8.80 |  |  |  |  |  |  |
| Xinjiang | 9.42 | 9.33 | 8.90 | 9.11 | 8.72 |  |  |  |  |  |  |

**Appendix A4**

**The Initial Physical Capital Stock (100 million RMB)**

| **Beijing** | 10.04 | **Shanghai** | 17.82 | **Hubei** | 12.72 | **Yunnan** | 28.96 |
| --- | --- | --- | --- | --- | --- | --- | --- |
| **Tianjin** | 17.60 | **Jiangsu** | 48.62 | **Hunan** | 12.55 | **Xizang** | 0.41 |
| **Hebei** | 45.70 | **Zhejiang** | 4.93 | **Guangdong** | 20.87 | **Shaanxi** | 9.72 |
| **Shanxi** | 23.43 | **Anhui** | 34.58 | **Guangxi** | 24.38 | **Gansu** | 26.54 |
| **Neimenggu** | 30.99 | **Fujian** | 10.13 | **Hainan** | 1.21 | **Qinghai** | 3.48 |
| **Liaoning** | 144.05 | **Jiangxi** | 16.21 | **Chongqing** | 13.01 | **Ningxia** | 2.57 |
| **Jinlin** | 32.99 | **Shandong** | 38.89 | **Sichuan** | 19.91 | **Xinjiang** | 9.67 |
| **Heilongjiang** | 30.19 | **Henan** | 29.30 | **Guizhou** | 3.93 |  |  |

**Appendix A5**

**Appendix A5: The Physical Capital Stock**

| **Province** | **1952** | **1953** | **1954** | **1955** | **1956** | **1957** | **1958** | **1959** | **1960** | **1961** | **1962** |
| --- | --- | --- | --- | --- | --- | --- | --- | --- | --- | --- | --- |
| Beijing | 10.04 | 13.15 | 16.28 | 18.54 | 22.20 | 24.98 | 30.65 | 40.64 | 48.47 | 48.58 | 46.45 |
| Tianjin | 17.60 | 17.77 | 18.10 | 18.23 | 18.92 | 19.53 | 24.75 | 29.44 | 35.00 | 34.02 | 34.06 |
| Hebei | 45.70 | 46.86 | 49.35 | 52.98 | 53.97 | 60.75 | 81.00 | 97.12 | 96.68 | 97.63 | 96.82 |
| Shanxi | 23.43 | 23.78 | 26.16 | 28.65 | 36.48 | 44.21 | 61.51 | 76.89 | 94.30 | 93.09 | 92.63 |
| Neimenggu | 30.99 | 28.98 | 28.28 | 26.59 | 27.53 | 26.89 | 36.11 | 44.19 | 55.13 | 53.34 | 52.88 |
| Liaoning | 144.05 | 161.20 | 183.00 | 199.53 | 220.83 | 231.79 | 301.25 | 376.91 | 466.76 | 434.74 | 437.03 |
| Jinlin | 32.99 | 33.10 | 35.64 | 38.29 | 42.40 | 42.00 | 46.63 | 51.49 | 56.40 | 56.38 | 56.63 |
| Heilongjiang | 30.19 | 32.27 | 35.55 | 38.75 | 43.55 | 47.13 | 56.96 | 66.36 | 80.42 | 77.03 | 78.29 |
| Shanghai | 17.82 | 18.64 | 18.84 | 19.98 | 22.05 | 22.46 | 30.14 | 40.24 | 50.14 | 49.86 | 50.58 |
| Jiangsu | 48.62 | 54.19 | 55.02 | 57.37 | 60.52 | 64.84 | 70.63 | 78.20 | 90.13 | 91.66 | 92.62 |
| Zhejiang | 4.93 | 5.23 | 5.60 | 5.99 | 6.58 | 7.34 | 9.52 | 11.64 | 13.81 | 14.22 | 14.65 |
| Anhui | 34.58 | 36.31 | 38.31 | 42.64 | 44.26 | 48.54 | 59.85 | 69.93 | 78.09 | 74.89 | 70.19 |
| Fujian | 10.13 | 9.91 | 10.68 | 12.28 | 18.32 | 19.44 | 25.22 | 31.39 | 38.06 | 37.20 | 36.70 |
| Jiangxi | 16.21 | 17.10 | 17.50 | 17.80 | 19.21 | 26.36 | 35.95 | 47.84 | 66.85 | 66.04 | 65.46 |
| Shandong | 38.89 | 41.03 | 44.21 | 48.59 | 55.16 | 61.14 | 71.82 | 83.42 | 96.76 | 96.07 | 95.88 |
| Henan | 29.30 | 29.42 | 30.74 | 34.56 | 40.13 | 46.36 | 62.04 | 80.78 | 101.88 | 100.00 | 101.58 |
| Hubei | 12.72 | 15.35 | 18.50 | 22.58 | 28.35 | 35.87 | 47.19 | 57.41 | 73.29 | 69.64 | 68.73 |
| Hunan | 12.55 | 12.85 | 13.59 | 14.02 | 15.34 | 17.18 | 28.66 | 40.22 | 51.79 | 50.13 | 49.22 |
| Guangdong | 20.87 | 23.43 | 24.99 | 26.05 | 28.86 | 31.49 | 43.98 | 54.96 | 65.64 | 63.90 | 61.33 |
| Guangxi | 24.38 | 26.07 | 29.13 | 32.58 | 35.34 | 35.98 | 40.84 | 48.54 | 54.67 | 53.89 | 53.24 |
| Hainan | 1.21 | 1.36 | 1.45 | 1.51 | 1.67 | 1.83 | 2.55 | 3.19 | 3.81 | 3.71 | 3.56 |
| Chongqing | 13.01 | 13.29 | 14.37 | 15.47 | 17.43 | 20.24 | 24.03 | 32.49 | 38.48 | 33.99 | 32.43 |
| Sichuan | 19.91 | 20.94 | 23.56 | 26.12 | 30.23 | 36.16 | 43.59 | 60.83 | 73.14 | 67.05 | 62.91 |
| Guizhou | 3.93 | 4.16 | 4.95 | 5.18 | 6.59 | 7.75 | 14.80 | 21.64 | 25.45 | 23.96 | 22.97 |
| Yunnan | 28.96 | 34.94 | 39.66 | 43.53 | 49.11 | 53.43 | 75.57 | 93.34 | 106.20 | 107.96 | 104.36 |
| Xizang | 0.41 | 0.42 | 0.42 | 0.45 | 1.39 | 1.56 | 1.52 | 1.75 | 2.94 | 2.91 | 2.97 |
| Shaanxi | 9.72 | 10.30 | 10.84 | 12.11 | 16.51 | 18.60 | 23.26 | 29.56 | 37.03 | 34.63 | 32.98 |
| Gansu | 26.54 | 29.32 | 32.32 | 35.27 | 45.25 | 51.88 | 66.48 | 79.90 | 93.48 | 90.30 | 87.38 |
| Qinghai | 3.48 | 3.72 | 4.40 | 5.48 | 9.93 | 11.52 | 12.79 | 19.61 | 24.03 | 22.86 | 22.34 |
| Ningxia | 2.57 | 2.75 | 3.11 | 3.34 | 4.01 | 4.44 | 5.95 | 7.54 | 9.06 | 9.15 | 9.22 |
| Xinjiang | 9.67 | 10.33 | 11.24 | 12.90 | 14.77 | 16.76 | 19.79 | 23.78 | 30.02 | 30.12 | 29.69 |
| **Province** | **1963** | **1964** | **1965** | **1966** | **1967** | **1968** | **1969** | **1970** | **1971** | **1972** | **1973** |
| Beijing | 45.36 | 46.92 | 49.82 | 52.84 | 53.05 | 54.53 | 57.70 | 56.29 | 57.19 | 60.33 | 69.61 |
| Tianjin | 34.28 | 35.02 | 34.65 | 35.20 | 33.55 | 33.49 | 34.70 | 38.14 | 41.76 | 47.32 | 57.52 |
| Hebei | 96.13 | 97.01 | 96.93 | 97.53 | 99.21 | 103.13 | 108.24 | 115.24 | 126.48 | 139.22 | 153.20 |
| Shanxi | 88.99 | 88.29 | 86.30 | 86.90 | 83.37 | 80.85 | 82.48 | 89.84 | 99.88 | 110.60 | 119.83 |
| Neimenggu | 52.25 | 51.99 | 53.87 | 55.17 | 52.35 | 52.45 | 51.28 | 53.26 | 54.23 | 55.89 | 58.04 |
| Liaoning | 445.61 | 430.52 | 420.04 | 397.47 | 372.44 | 365.01 | 355.08 | 339.52 | 333.74 | 322.43 | 319.77 |
| Jinlin | 56.54 | 56.87 | 58.66 | 58.96 | 57.78 | 57.97 | 58.60 | 60.75 | 63.96 | 68.32 | 75.40 |
| Heilongjiang | 79.49 | 80.73 | 81.70 | 80.58 | 77.59 | 76.25 | 78.19 | 80.21 | 82.45 | 87.45 | 94.44 |
| Shanghai | 52.05 | 53.12 | 54.29 | 54.51 | 52.96 | 53.14 | 54.41 | 59.02 | 64.40 | 72.02 | 82.34 |
| Jiangsu | 94.77 | 96.02 | 96.92 | 99.16 | 97.82 | 99.74 | 101.25 | 106.33 | 113.19 | 122.55 | 133.91 |
| Zhejiang | 14.91 | 15.28 | 15.73 | 16.31 | 16.78 | 17.30 | 18.52 | 19.73 | 21.12 | 23.50 | 25.11 |
| Anhui | 65.42 | 65.09 | 65.75 | 65.04 | 62.94 | 61.94 | 62.09 | 65.54 | 69.35 | 74.92 | 80.84 |
| Fujian | 35.63 | 36.48 | 36.97 | 37.64 | 34.77 | 32.83 | 31.41 | 35.17 | 42.61 | 48.80 | 53.47 |
| Jiangxi | 64.36 | 64.16 | 74.67 | 76.59 | 77.35 | 79.62 | 81.08 | 92.67 | 106.07 | 113.48 | 119.15 |
| Shandong | 93.45 | 92.31 | 94.29 | 99.28 | 102.57 | 106.29 | 113.87 | 122.39 | 130.89 | 139.91 | 150.70 |
| Henan | 99.92 | 100.40 | 99.34 | 103.79 | 102.90 | 101.71 | 108.56 | 123.47 | 134.32 | 146.13 | 159.73 |
| Hubei | 65.95 | 62.81 | 61.80 | 64.94 | 67.16 | 66.61 | 71.71 | 98.95 | 116.43 | 128.29 | 134.61 |
| Hunan | 47.27 | 49.81 | 52.21 | 56.36 | 58.46 | 59.50 | 63.63 | 74.27 | 87.24 | 96.60 | 105.00 |
| Guangdong | 59.27 | 60.18 | 61.17 | 60.81 | 58.44 | 56.98 | 61.23 | 72.78 | 86.32 | 96.73 | 111.09 |
| Guangxi | 53.27 | 53.70 | 55.60 | 60.13 | 60.16 | 62.07 | 69.01 | 73.48 | 81.17 | 87.42 | 93.84 |
| Hainan | 3.44 | 3.49 | 3.55 | 3.53 | 3.39 | 3.30 | 3.55 | 4.22 | 5.01 | 5.61 | 6.44 |
| Chongqing | 31.61 | 31.96 | 37.19 | 46.06 | 49.93 | 49.13 | 55.41 | 65.01 | 69.97 | 75.59 | 82.64 |
| Sichuan | 61.33 | 62.21 | 72.62 | 90.00 | 96.81 | 94.27 | 106.39 | 125.01 | 136.42 | 148.62 | 163.03 |
| Guizhou | 22.74 | 24.59 | 31.79 | 39.35 | 41.91 | 43.56 | 46.71 | 55.49 | 68.60 | 76.35 | 80.52 |
| Yunnan | 102.84 | 106.43 | 113.73 | 121.44 | 121.98 | 134.08 | 144.26 | 145.27 | 146.80 | 148.79 | 149.44 |
| Xizang | 2.97 | 3.11 | 3.52 | 4.20 | 4.82 | 4.61 | 4.72 | 5.04 | 5.71 | 6.34 | 7.01 |
| Shaanxi | 31.51 | 31.78 | 34.36 | 38.81 | 39.78 | 39.44 | 45.66 | 58.20 | 75.86 | 90.72 | 103.85 |
| Gansu | 82.29 | 79.92 | 85.02 | 88.17 | 91.79 | 96.23 | 98.49 | 102.96 | 107.09 | 110.97 | 115.50 |
| Qinghai | 21.39 | 20.46 | 20.26 | 21.55 | 21.56 | 21.64 | 23.37 | 25.26 | 26.28 | 32.29 | 32.46 |
| Ningxia | 9.38 | 10.15 | 11.54 | 13.57 | 15.34 | 16.17 | 17.70 | 20.19 | 23.49 | 26.39 | 29.41 |
| Xinjiang | 31.11 | 32.57 | 34.92 | 36.30 | 36.22 | 36.47 | 36.95 | 38.52 | 39.31 | 39.98 | 42.53 |
| **Province** | **1974** | **1975** | **1976** | **1977** | **1978** | **1979** | **1980** | **1981** | **1982** | **1983** | **1984** |
| Beijing | 80.70 | 88.17 | 80.63 | 86.21 | 101.59 | 119.88 | 142.74 | 157.09 | 174.35 | 206.01 | 251.70 |
| Tianjin | 71.03 | 83.56 | 91.78 | 103.04 | 110.67 | 120.40 | 131.47 | 140.89 | 157.00 | 176.81 | 202.42 |
| Hebei | 168.00 | 191.38 | 211.16 | 233.07 | 256.29 | 284.76 | 305.15 | 318.20 | 348.68 | 378.02 | 403.14 |
| Shanxi | 126.66 | 134.67 | 139.42 | 149.82 | 160.19 | 163.78 | 172.34 | 176.13 | 188.62 | 208.55 | 238.61 |
| Neimenggu | 61.02 | 65.54 | 69.48 | 75.08 | 83.43 | 91.89 | 97.89 | 104.15 | 114.42 | 131.34 | 155.32 |
| Liaoning | 315.49 | 309.43 | 303.40 | 301.62 | 304.37 | 312.76 | 321.85 | 328.51 | 352.87 | 378.76 | 415.07 |
| Jinlin | 80.01 | 86.87 | 88.89 | 92.09 | 100.16 | 107.92 | 117.56 | 125.25 | 138.13 | 150.59 | 166.44 |
| Heilongjiang | 101.18 | 109.13 | 117.64 | 127.86 | 140.53 | 152.70 | 174.77 | 201.62 | 241.62 | 272.10 | 316.39 |
| Shanghai | 97.42 | 121.17 | 134.16 | 139.54 | 155.49 | 174.81 | 198.90 | 230.76 | 275.01 | 318.47 | 369.28 |
| Jiangsu | 141.53 | 153.02 | 165.33 | 178.43 | 199.36 | 221.99 | 252.98 | 285.50 | 355.22 | 430.95 | 517.57 |
| Zhejiang | 26.96 | 28.53 | 30.55 | 32.62 | 34.33 | 35.76 | 39.58 | 43.00 | 47.74 | 52.39 | 60.58 |
| Anhui | 86.95 | 88.67 | 95.01 | 99.96 | 105.89 | 110.29 | 114.56 | 117.48 | 132.91 | 154.89 | 190.11 |
| Fujian | 57.00 | 58.33 | 57.42 | 56.48 | 62.95 | 71.25 | 82.67 | 91.35 | 102.45 | 117.15 | 133.52 |
| Jiangxi | 123.47 | 124.60 | 125.53 | 128.66 | 143.37 | 159.07 | 173.25 | 182.10 | 198.25 | 219.28 | 242.64 |
| Shandong | 159.14 | 191.90 | 212.17 | 245.78 | 279.81 | 311.59 | 345.90 | 379.26 | 428.60 | 479.47 | 548.92 |
| Henan | 174.40 | 190.48 | 201.32 | 216.95 | 232.94 | 248.58 | 274.60 | 294.25 | 324.28 | 370.82 | 408.14 |
| Hubei | 140.81 | 157.94 | 182.91 | 207.33 | 213.79 | 217.47 | 221.40 | 225.27 | 241.59 | 261.06 | 293.74 |
| Hunan | 111.45 | 121.95 | 126.21 | 130.83 | 143.72 | 155.80 | 170.40 | 179.77 | 194.32 | 215.31 | 230.58 |
| Guangdong | 128.61 | 152.11 | 170.80 | 186.23 | 203.58 | 221.97 | 246.04 | 276.78 | 325.63 | 368.27 | 424.14 |
| Guangxi | 99.14 | 106.31 | 111.50 | 118.85 | 126.96 | 131.64 | 141.94 | 148.08 | 147.36 | 150.17 | 160.40 |
| Hainan | 7.46 | 8.82 | 9.91 | 10.80 | 11.81 | 12.66 | 13.24 | 14.65 | 18.88 | 21.96 | 26.63 |
| Chongqing | 88.33 | 99.84 | 102.21 | 109.26 | 118.62 | 127.88 | 136.58 | 143.82 | 154.33 | 166.97 | 183.32 |
| Sichuan | 173.35 | 196.14 | 199.39 | 209.79 | 229.47 | 247.67 | 265.13 | 278.88 | 300.18 | 324.32 | 356.13 |
| Guizhou | 81.09 | 84.69 | 84.00 | 87.22 | 93.41 | 99.15 | 104.84 | 110.89 | 118.16 | 127.25 | 140.72 |
| Yunnan | 149.02 | 153.55 | 154.47 | 160.25 | 161.94 | 167.95 | 177.15 | 181.14 | 184.94 | 185.18 | 190.98 |
| Xizang | 6.98 | 7.29 | 7.56 | 8.04 | 9.00 | 10.09 | 10.85 | 10.76 | 11.06 | 11.62 | 14.79 |
| Shaanxi | 112.75 | 121.07 | 125.06 | 132.04 | 139.90 | 149.95 | 161.13 | 172.39 | 184.21 | 192.92 | 215.22 |
| Gansu | 120.27 | 125.06 | 128.23 | 133.12 | 147.55 | 157.02 | 160.09 | 158.24 | 161.21 | 163.84 | 170.98 |
| Qinghai | 31.44 | 31.59 | 31.92 | 34.49 | 39.57 | 46.02 | 49.72 | 50.54 | 55.91 | 57.97 | 60.96 |
| Ningxia | 32.22 | 36.17 | 39.16 | 43.31 | 46.55 | 50.07 | 51.93 | 51.71 | 53.84 | 56.34 | 59.31 |
| Xinjiang | 45.58 | 48.15 | 49.68 | 54.16 | 60.80 | 69.33 | 77.50 | 86.46 | 99.25 | 113.92 | 130.15 |
| **Province** | **1985** | **1986** | **1987** | **1988** | **1989** | **1990** | **1991** | **1992** | **1993** | **1994** | **1995** |
| Beijing | 321.44 | 417.03 | 543.66 | 683.50 | 808.28 | 956.34 | 1087.74 | 1252.80 | 1458.77 | 1764.06 | 2097.66 |
| Tianjin | 231.96 | 262.63 | 291.96 | 318.08 | 335.90 | 353.65 | 383.34 | 414.46 | 445.33 | 492.08 | 544.49 |
| Hebei | 442.40 | 484.88 | 537.16 | 596.96 | 644.53 | 697.74 | 765.03 | 846.33 | 928.77 | 1037.13 | 1194.03 |
| Shanxi | 285.76 | 326.94 | 367.13 | 389.19 | 404.25 | 422.63 | 448.49 | 473.07 | 498.92 | 529.31 | 558.49 |
| Neimenggu | 182.40 | 200.67 | 222.62 | 252.62 | 271.67 | 293.44 | 332.92 | 381.36 | 451.74 | 521.99 | 591.09 |
| Liaoning | 467.72 | 527.50 | 605.64 | 685.31 | 748.67 | 817.34 | 900.55 | 975.83 | 1090.53 | 1197.18 | 1285.61 |
| Jinlin | 195.66 | 221.00 | 251.92 | 281.78 | 294.08 | 317.44 | 348.29 | 384.36 | 445.34 | 503.98 | 562.70 |
| Heilongjiang | 368.05 | 415.73 | 464.08 | 506.13 | 529.87 | 555.24 | 584.11 | 618.15 | 654.36 | 705.68 | 775.37 |
| Shanghai | 426.72 | 490.80 | 573.17 | 663.57 | 725.26 | 791.17 | 855.13 | 944.93 | 1060.27 | 1266.05 | 1561.56 |
| Jiangsu | 634.08 | 787.02 | 961.60 | 1156.56 | 1292.45 | 1466.53 | 1673.11 | 2008.44 | 2349.92 | 2689.90 | 3086.52 |
| Zhejiang | 74.07 | 88.84 | 106.78 | 124.03 | 136.78 | 151.72 | 169.89 | 194.62 | 213.21 | 240.57 | 276.87 |
| Anhui | 232.76 | 274.98 | 317.78 | 354.72 | 385.26 | 420.70 | 449.76 | 485.18 | 532.41 | 590.64 | 678.30 |
| Fujian | 150.68 | 172.40 | 197.86 | 215.40 | 227.78 | 240.35 | 259.46 | 283.57 | 319.21 | 382.48 | 462.41 |
| Jiangxi | 267.41 | 305.69 | 332.84 | 344.77 | 375.42 | 404.06 | 429.84 | 479.82 | 544.72 | 616.13 | 692.84 |
| Shandong | 632.27 | 723.58 | 841.70 | 943.54 | 1020.77 | 1111.53 | 1228.92 | 1353.86 | 1488.17 | 1612.05 | 1767.48 |
| Henan | 462.49 | 519.82 | 571.91 | 649.83 | 709.40 | 766.76 | 836.65 | 902.26 | 969.99 | 1079.00 | 1229.66 |
| Hubei | 329.63 | 361.70 | 407.07 | 447.51 | 473.19 | 513.42 | 558.29 | 611.04 | 681.46 | 800.74 | 964.00 |
| Hunan | 255.24 | 285.94 | 321.43 | 355.86 | 365.47 | 394.48 | 433.73 | 484.35 | 539.02 | 600.73 | 675.59 |
| Guangdong | 482.10 | 543.19 | 608.05 | 685.22 | 764.75 | 869.10 | 986.24 | 1168.75 | 1445.15 | 1772.88 | 2159.76 |
| Guangxi | 172.26 | 188.34 | 205.59 | 212.36 | 218.66 | 224.71 | 237.92 | 258.32 | 293.84 | 340.09 | 387.85 |
| Hainan | 34.52 | 42.52 | 51.09 | 57.61 | 68.51 | 84.14 | 99.67 | 122.40 | 143.86 | 166.51 | 187.23 |
| Chongqing | 199.94 | 216.13 | 234.25 | 253.14 | 265.17 | 285.75 | 309.42 | 336.58 | 366.60 | 405.09 | 458.68 |
| Sichuan | 390.23 | 420.37 | 457.33 | 492.44 | 517.16 | 558.32 | 603.15 | 656.29 | 717.71 | 796.67 | 911.00 |
| Guizhou | 159.04 | 172.82 | 187.19 | 201.03 | 208.10 | 219.30 | 231.31 | 243.35 | 256.10 | 274.29 | 296.98 |
| Yunnan | 195.58 | 201.33 | 210.70 | 221.75 | 229.36 | 240.75 | 267.35 | 300.87 | 330.81 | 362.68 | 400.25 |
| Xizang | 18.78 | 20.68 | 22.87 | 24.72 | 27.21 | 29.84 | 33.66 | 38.52 | 45.37 | 54.15 | 71.43 |
| Shaanxi | 250.89 | 303.03 | 345.12 | 387.28 | 413.26 | 442.25 | 471.10 | 503.04 | 543.39 | 585.48 | 636.21 |
| Gansu | 187.22 | 207.66 | 230.19 | 248.36 | 264.75 | 284.23 | 304.55 | 320.09 | 328.47 | 337.65 | 352.81 |
| Qinghai | 66.32 | 70.64 | 77.27 | 84.23 | 87.50 | 94.40 | 99.99 | 105.68 | 112.36 | 119.40 | 128.62 |
| Ningxia | 66.33 | 73.33 | 81.13 | 86.35 | 88.58 | 91.95 | 96.90 | 101.63 | 107.09 | 112.53 | 118.32 |
| Xinjiang | 148.14 | 164.72 | 178.55 | 197.93 | 215.40 | 243.02 | 275.44 | 318.42 | 367.30 | 434.52 | 487.78 |
| **Province** | **1996** | **1997** | **1998** | **1999** | **2000** | **2001** | **2002** | **2003** | **2004** | **2005** | **2006** |
| Beijing | 2387.19 | 2687.60 | 3049.72 | 3387.35 | 3746.30 | 4161.02 | 4705.08 | 5414.88 | 6193.62 | 7026.89 | 7939.04 |
| Tianjin | 608.92 | 681.77 | 768.69 | 848.63 | 935.38 | 1040.45 | 1164.74 | 1332.64 | 1520.53 | 1751.71 | 2038.98 |
| Hebei | 1407.98 | 1661.02 | 1934.08 | 2229.17 | 2506.03 | 2780.79 | 3061.94 | 3423.08 | 3887.53 | 4518.00 | 5266.01 |
| Shanxi | 591.89 | 642.55 | 723.40 | 809.70 | 898.13 | 994.91 | 1115.10 | 1275.27 | 1489.40 | 1760.90 | 2095.36 |
| Neimenggu | 650.80 | 720.50 | 795.59 | 876.23 | 967.16 | 1073.83 | 1256.12 | 1606.49 | 2109.20 | 2838.75 | 3674.94 |
| Liaoning | 1379.30 | 1469.49 | 1567.80 | 1669.40 | 1798.59 | 1946.26 | 2117.82 | 2367.04 | 2761.84 | 3233.49 | 3851.96 |
| Jinlin | 627.67 | 670.78 | 725.25 | 794.83 | 882.55 | 977.40 | 1092.69 | 1239.42 | 1432.88 | 1721.40 | 2209.46 |
| Heilongjiang | 859.05 | 946.08 | 1053.75 | 1152.25 | 1253.07 | 1371.54 | 1500.40 | 1634.19 | 1795.40 | 1988.97 | 2246.64 |
| Shanghai | 1900.26 | 2227.25 | 2524.30 | 2808.17 | 3100.68 | 3405.89 | 3758.07 | 4136.91 | 4584.99 | 5104.22 | 5724.25 |
| Jiangsu | 3551.57 | 4075.71 | 4680.08 | 5328.10 | 6048.82 | 6803.90 | 7637.95 | 8866.51 | 10277.32 | 12092.19 | 14095.48 |
| Zhejiang | 322.86 | 384.01 | 467.98 | 580.60 | 729.41 | 901.57 | 1108.50 | 1305.39 | 1511.66 | 2797.29 | 4104.73 |
| Anhui | 776.32 | 879.36 | 980.12 | 1080.31 | 1186.77 | 1304.20 | 1434.45 | 1594.22 | 1830.12 | 2096.06 | 2417.48 |
| Fujian | 556.19 | 657.69 | 775.55 | 892.72 | 1009.11 | 1123.94 | 1247.70 | 1403.61 | 1602.19 | 1867.26 | 2200.37 |
| Jiangxi | 786.85 | 898.10 | 1007.50 | 1127.62 | 1252.07 | 1402.39 | 1629.94 | 1942.68 | 2312.23 | 2739.29 | 3221.63 |
| Shandong | 1971.78 | 2214.61 | 2496.91 | 2823.81 | 3218.82 | 3638.45 | 4149.88 | 4782.20 | 5593.71 | 6622.40 | 7800.82 |
| Henan | 1417.21 | 1627.33 | 1866.85 | 2111.39 | 2368.61 | 2636.23 | 2949.94 | 3325.75 | 3794.56 | 4512.88 | 5499.65 |
| Hubei | 1168.45 | 1382.51 | 1609.09 | 1845.41 | 2090.89 | 2355.98 | 2619.90 | 2891.93 | 3225.44 | 3631.67 | 4173.09 |
| Hunan | 774.74 | 870.74 | 979.26 | 1104.48 | 1240.43 | 1392.47 | 1560.81 | 1755.36 | 1985.83 | 2290.94 | 2656.44 |
| Guangdong | 2534.72 | 2896.80 | 3327.81 | 3849.64 | 4365.61 | 4922.72 | 5599.26 | 6450.93 | 7370.41 | 8550.14 | 9872.47 |
| Guangxi | 442.30 | 492.94 | 553.95 | 623.96 | 694.30 | 767.42 | 853.09 | 953.86 | 1091.97 | 1282.90 | 1536.94 |
| Hainan | 201.71 | 215.18 | 228.80 | 245.26 | 260.57 | 276.20 | 294.80 | 317.85 | 344.07 | 376.20 | 415.29 |
| Chongqing | 485.97 | 528.15 | 594.25 | 665.24 | 741.64 | 836.38 | 957.37 | 1125.58 | 1323.00 | 1561.70 | 1824.01 |
| Sichuan | 1042.98 | 1190.13 | 1365.93 | 1535.33 | 1734.99 | 1947.66 | 2192.07 | 2483.30 | 2807.74 | 3199.88 | 3700.68 |
| Guizhou | 328.39 | 368.23 | 418.72 | 480.59 | 548.94 | 640.68 | 744.87 | 861.56 | 980.52 | 1111.86 | 1262.42 |
| Yunnan | 445.91 | 497.89 | 566.48 | 634.59 | 691.32 | 748.71 | 815.69 | 905.18 | 1015.25 | 1159.75 | 1343.44 |
| Xizang | 84.51 | 98.96 | 115.34 | 132.52 | 144.65 | 146.04 | 157.23 | 179.22 | 206.26 | 237.49 | 274.93 |
| Shaanxi | 688.81 | 745.56 | 821.30 | 906.69 | 1016.50 | 1132.04 | 1263.44 | 1454.13 | 1640.07 | 1890.23 | 2214.60 |
| Gansu | 379.70 | 414.77 | 455.19 | 507.72 | 572.25 | 654.63 | 749.84 | 857.70 | 983.07 | 1122.38 | 1277.28 |
| Qinghai | 144.61 | 165.92 | 190.36 | 217.72 | 250.73 | 295.80 | 347.27 | 404.45 | 463.53 | 529.41 | 599.99 |
| Ningxia | 124.21 | 131.37 | 141.97 | 156.43 | 174.47 | 197.23 | 224.58 | 265.92 | 311.02 | 362.50 | 420.55 |
| Xinjiang | 544.83 | 603.65 | 671.02 | 736.00 | 811.95 | 891.48 | 993.34 | 1128.14 | 1268.45 | 1436.10 | 1626.15 |
| **Province** | **2007** | **2008** | **2009** | **2010** | **2011** | **2012** | **2013** | **2014** | **2015** | **2016** | **2017** |
| Beijing | 8948.51 | 9571.14 | 10512.12 | 11692.71 | 12875.72 | 14389.16 | 15990.93 | 17588.28 | 19210.81 | 21333.78 | 23357.49 |
| Tianjin | 2408.10 | 2901.30 | 3629.63 | 4535.09 | 5572.74 | 6749.91 | 8046.03 | 9383.81 | 10456.96 | 11368.74 | 12129.76 |
| Hebei | 6153.87 | 7291.20 | 8609.87 | 10025.76 | 11741.19 | 13583.32 | 15487.41 | 17406.67 | 19301.42 | 21298.66 | 22970.31 |
| Shanxi | 2495.60 | 2907.63 | 3541.87 | 4292.56 | 5128.15 | 5944.19 | 6863.21 | 7729.17 | 8545.59 | 9205.46 | 9442.67 |
| Neimenggu | 4729.20 | 5940.60 | 7631.15 | 9477.56 | 11519.06 | 13912.31 | 16767.55 | 18875.48 | 21003.20 | 22553.92 | 23287.21 |
| Liaoning | 4609.64 | 5891.27 | 6894.81 | 8123.14 | 9500.69 | 11006.80 | 12629.57 | 14189.72 | 14889.00 | 15079.10 | 15370.13 |
| Jinlin | 2891.80 | 3779.40 | 4719.71 | 5824.11 | 6871.66 | 7988.69 | 9109.36 | 10275.21 | 11533.57 | 12533.55 | 13315.32 |
| Heilongjiang | 2587.63 | 2989.24 | 3618.82 | 4214.52 | 4874.19 | 5670.61 | 6620.62 | 7514.63 | 8424.90 | 9170.22 | 9882.04 |
| Shanghai | 6440.42 | 7059.70 | 7943.63 | 8680.26 | 9325.60 | 9999.89 | 10753.83 | 11521.47 | 12518.81 | 13814.37 | 15036.69 |
| Jiangsu | 16226.95 | 18606.14 | 21670.19 | 25185.14 | 28957.23 | 33032.84 | 37044.04 | 40895.95 | 45049.91 | 49543.78 | 54129.73 |
| Zhejiang | 5448.28 | 6721.52 | 8124.98 | 9676.39 | 11188.46 | 12761.93 | 14479.32 | 16172.61 | 18035.86 | 20195.35 | 22172.45 |
| Anhui | 2807.88 | 3265.13 | 3805.39 | 4463.12 | 5212.94 | 6060.40 | 6997.46 | 8007.58 | 9051.20 | 10217.52 | 11329.08 |
| Fujian | 2619.74 | 3136.11 | 3740.77 | 4384.97 | 5123.21 | 5958.21 | 6900.81 | 7928.02 | 9045.93 | 10254.59 | 11514.31 |
| Jiangxi | 3747.65 | 4284.11 | 5069.75 | 5908.11 | 6826.51 | 7791.78 | 8790.88 | 9726.88 | 10869.26 | 12267.50 | 13583.84 |
| Shandong | 9027.80 | 10365.01 | 12260.47 | 14362.17 | 16559.15 | 18934.27 | 21449.22 | 24076.84 | 26842.96 | 29452.10 | 31735.57 |
| Henan | 6798.90 | 8318.30 | 10351.25 | 12680.86 | 15142.61 | 17914.83 | 20989.30 | 24246.88 | 27555.32 | 30988.38 | 33862.02 |
| Hubei | 4823.72 | 5533.66 | 6475.53 | 7606.36 | 8980.78 | 10493.41 | 12198.88 | 14075.10 | 16065.89 | 18224.02 | 20392.28 |
| Hunan | 3101.40 | 3684.71 | 4412.43 | 5327.85 | 6337.87 | 7460.16 | 8689.99 | 10007.41 | 11190.58 | 12459.36 | 13589.24 |
| Guangdong | 11376.28 | 12866.97 | 14997.64 | 17498.10 | 20080.28 | 22998.27 | 26263.18 | 29771.60 | 33334.41 | 37412.11 | 41855.29 |
| Guangxi | 1865.44 | 2237.64 | 2913.26 | 3851.64 | 4912.16 | 5989.20 | 6837.18 | 7696.47 | 8628.85 | 9609.63 | 9991.16 |
| Hainan | 458.85 | 524.74 | 609.36 | 724.49 | 855.60 | 1034.53 | 1240.96 | 1472.81 | 1652.23 | 1843.03 | 2043.64 |
| Chongqing | 2121.59 | 2511.87 | 2878.41 | 3322.18 | 3853.42 | 4405.32 | 4993.85 | 5655.02 | 6390.63 | 7242.08 | 8066.74 |
| Sichuan | 4315.73 | 5031.72 | 5875.93 | 6832.23 | 7906.11 | 9098.12 | 10347.02 | 11641.75 | 12957.61 | 14394.09 | 15815.25 |
| Guizhou | 1436.01 | 1631.09 | 1882.76 | 2189.84 | 2557.71 | 3049.82 | 3689.05 | 4402.64 | 5257.23 | 6246.89 | 7257.66 |
| Yunnan | 1554.91 | 1718.60 | 1987.42 | 2430.57 | 2973.83 | 3600.12 | 4313.68 | 5135.14 | 6045.57 | 7006.66 | 7976.14 |
| Xizang | 317.65 | 363.41 | 423.16 | 521.67 | 601.26 | 708.27 | 844.21 | 996.62 | 1126.92 | 1266.59 | 1439.35 |
| Shaanxi | 2636.19 | 3221.39 | 3878.57 | 4734.45 | 5658.20 | 6718.97 | 7826.04 | 8985.17 | 10050.10 | 11184.80 | 12367.75 |
| Gansu | 1460.18 | 1727.97 | 1977.24 | 2278.23 | 2635.01 | 3041.46 | 3516.83 | 4052.30 | 4631.82 | 5279.26 | 5526.33 |
| Qinghai | 678.79 | 763.16 | 892.37 | 1071.97 | 1285.98 | 1599.01 | 1997.06 | 2476.94 | 3009.43 | 3554.04 | 4083.47 |
| Ningxia | 488.31 | 579.11 | 714.39 | 876.90 | 1028.50 | 1209.74 | 1411.58 | 1705.00 | 2063.98 | 2425.96 | 2734.76 |
| Xinjiang | 1837.13 | 2023.34 | 2242.24 | 2555.90 | 2907.54 | 3458.34 | 4176.57 | 5034.23 | 5908.05 | 6687.27 | 7670.13 |
| **Province** | **2018** | **2019** | **2020** | **2021** | **2022** |  |  |  |  |  |  |
| Beijing | 24127.63 | 24693.93 | 25276.71 | 26058.30 | 26675.36 |  |  |  |  |  |  |
| Tianjin | 12904.32 | 13820.03 | 14689.55 | 15619.49 | 16154.69 |  |  |  |  |  |  |
| Hebei | 27366.28 | 31570.49 | 35521.27 | 39333.83 | 43129.95 |  |  |  |  |  |  |
| Shanxi | 9567.17 | 9734.71 | 10005.73 | 10392.95 | 10736.94 |  |  |  |  |  |  |
| Neimenggu | 23849.38 | 24462.56 | 24987.67 | 25787.80 | 26792.74 |  |  |  |  |  |  |
| Liaoning | 15054.84 | 14716.50 | 14442.83 | 14294.61 | 14117.08 |  |  |  |  |  |  |
| Jinlin | 14625.55 | 15358.00 | 16217.76 | 17237.11 | 17989.86 |  |  |  |  |  |  |
| Heilongjiang | 10656.05 | 11469.08 | 12260.57 | 13124.37 | 13830.56 |  |  |  |  |  |  |
| Shanghai | 15204.32 | 15419.74 | 15793.15 | 16343.51 | 16715.41 |  |  |  |  |  |  |
| Jiangsu | 62622.18 | 70852.15 | 78204.31 | 85657.33 | 92284.03 |  |  |  |  |  |  |
| Zhejiang | 25812.20 | 29568.67 | 33262.97 | 37312.90 | 41277.65 |  |  |  |  |  |  |
| Anhui | 15042.25 | 18729.20 | 22266.30 | 25991.18 | 29659.07 |  |  |  |  |  |  |
| Fujian | 13999.98 | 16400.04 | 18533.38 | 20751.81 | 22888.08 |  |  |  |  |  |  |
| Jiangxi | 18228.79 | 22822.00 | 27422.04 | 32384.77 | 37225.80 |  |  |  |  |  |  |
| Shandong | 36702.60 | 40378.20 | 43893.94 | 47564.34 | 51038.89 |  |  |  |  |  |  |
| Henan | 39435.12 | 44893.84 | 50212.06 | 55674.77 | 60851.29 |  |  |  |  |  |  |
| Hubei | 24862.73 | 29319.55 | 31980.50 | 35867.69 | 39629.44 |  |  |  |  |  |  |
| Hunan | 16851.72 | 20182.18 | 23524.59 | 27007.40 | 30298.75 |  |  |  |  |  |  |
| Guangdong | 46104.54 | 50529.10 | 55171.82 | 60133.33 | 63920.79 |  |  |  |  |  |  |
| Guangxi | 12181.33 | 14388.15 | 16503.87 | 18708.24 | 20597.54 |  |  |  |  |  |  |
| Hainan | 2319.87 | 2516.72 | 2723.61 | 2960.47 | 3128.64 |  |  |  |  |  |  |
| Chongqing | 10070.06 | 11941.76 | 13692.08 | 15465.81 | 16983.89 |  |  |  |  |  |  |
| Sichuan | 19547.44 | 23285.38 | 26703.97 | 30188.97 | 33452.92 |  |  |  |  |  |  |
| Guizhou | 9609.26 | 11698.73 | 13653.64 | 15364.78 | 16658.65 |  |  |  |  |  |  |
| Yunnan | 9443.79 | 10910.51 | 12377.81 | 13839.74 | 15255.66 |  |  |  |  |  |  |
| Xizang | 1762.27 | 2036.06 | 2302.13 | 2476.08 | 2561.76 |  |  |  |  |  |  |
| Shaanxi | 15168.97 | 17693.06 | 20118.17 | 22240.29 | 24302.59 |  |  |  |  |  |  |
| Gansu | 6158.37 | 6760.25 | 7388.42 | 8098.74 | 8818.01 |  |  |  |  |  |  |
| Qinghai | 4584.69 | 5060.50 | 5370.32 | 5651.24 | 5814.05 |  |  |  |  |  |  |
| Ningxia | 2890.34 | 2983.08 | 3083.82 | 3188.67 | 3309.00 |  |  |  |  |  |  |
| Xinjiang | 8263.06 | 8808.39 | 9515.16 | 10392.19 | 11249.93 |  |  |  |  |  |  |
| **Province** | **2018** | **2019** | **2020** | **2021** | **2022** |  |  |  |  |  |  |
| Beijing | 24127.63 | 24693.93 | 25276.71 | 26058.30 | 26675.36 |  |  |  |  |  |  |
| Tianjin | 12904.32 | 13820.03 | 14689.55 | 15619.49 | 16154.69 |  |  |  |  |  |  |
| Hebei | 27366.28 | 31570.49 | 35521.27 | 39333.83 | 43129.95 |  |  |  |  |  |  |
| Shanxi | 9567.17 | 9734.71 | 10005.73 | 10392.95 | 10736.94 |  |  |  |  |  |  |
| Neimenggu | 23849.38 | 24462.56 | 24987.67 | 25787.80 | 26792.74 |  |  |  |  |  |  |
| Liaoning | 15054.84 | 14716.50 | 14442.83 | 14294.61 | 14117.08 |  |  |  |  |  |  |
| Jinlin | 14625.55 | 15358.00 | 16217.76 | 17237.11 | 17989.86 |  |  |  |  |  |  |
| Heilongjiang | 10656.05 | 11469.08 | 12260.57 | 13124.37 | 13830.56 |  |  |  |  |  |  |
| Shanghai | 15204.32 | 15419.74 | 15793.15 | 16343.51 | 16715.41 |  |  |  |  |  |  |
| Jiangsu | 62622.18 | 70852.15 | 78204.31 | 85657.33 | 92284.03 |  |  |  |  |  |  |
| Zhejiang | 25812.20 | 29568.67 | 33262.97 | 37312.90 | 41277.65 |  |  |  |  |  |  |
| Anhui | 15042.25 | 18729.20 | 22266.30 | 25991.18 | 29659.07 |  |  |  |  |  |  |
| Fujian | 13999.98 | 16400.04 | 18533.38 | 20751.81 | 22888.08 |  |  |  |  |  |  |
| Jiangxi | 18228.79 | 22822.00 | 27422.04 | 32384.77 | 37225.80 |  |  |  |  |  |  |
| Shandong | 36702.60 | 40378.20 | 43893.94 | 47564.34 | 51038.89 |  |  |  |  |  |  |
| Henan | 39435.12 | 44893.84 | 50212.06 | 55674.77 | 60851.29 |  |  |  |  |  |  |
| Hubei | 24862.73 | 29319.55 | 31980.50 | 35867.69 | 39629.44 |  |  |  |  |  |  |
| Hunan | 16851.72 | 20182.18 | 23524.59 | 27007.40 | 30298.75 |  |  |  |  |  |  |
| Guangdong | 46104.54 | 50529.10 | 55171.82 | 60133.33 | 63920.79 |  |  |  |  |  |  |
| Guangxi | 12181.33 | 14388.15 | 16503.87 | 18708.24 | 20597.54 |  |  |  |  |  |  |
| Hainan | 2319.87 | 2516.72 | 2723.61 | 2960.47 | 3128.64 |  |  |  |  |  |  |
| Chongqing | 10070.06 | 11941.76 | 13692.08 | 15465.81 | 16983.89 |  |  |  |  |  |  |
| Sichuan | 19547.44 | 23285.38 | 26703.97 | 30188.97 | 33452.92 |  |  |  |  |  |  |
| Guizhou | 9609.26 | 11698.73 | 13653.64 | 15364.78 | 16658.65 |  |  |  |  |  |  |
| Yunnan | 9443.79 | 10910.51 | 12377.81 | 13839.74 | 15255.66 |  |  |  |  |  |  |
| Xizang | 1762.27 | 2036.06 | 2302.13 | 2476.08 | 2561.76 |  |  |  |  |  |  |
| Shaanxi | 15168.97 | 17693.06 | 20118.17 | 22240.29 | 24302.59 |  |  |  |  |  |  |
| Gansu | 6158.37 | 6760.25 | 7388.42 | 8098.74 | 8818.01 |  |  |  |  |  |  |
| Qinghai | 4584.69 | 5060.50 | 5370.32 | 5651.24 | 5814.05 |  |  |  |  |  |  |
| Ningxia | 2890.34 | 2983.08 | 3083.82 | 3188.67 | 3309.00 |  |  |  |  |  |  |
| Xinjiang | 8263.06 | 8808.39 | 9515.16 | 10392.19 | 11249.93 |  |  |  |  |  |  |
